# American Gut: an Open Platform for Citizen Science Microbiome Research

**DOI:** 10.1128/mSystems.00031-18

**Published:** 2018-05-15

**Authors:** Daniel McDonald, Embriette Hyde, Justine W. Debelius, James T. Morton, Antonio Gonzalez, Gail Ackermann, Alexander A. Aksenov, Bahar Behsaz, Caitriona Brennan, Yingfeng Chen, Lindsay DeRight Goldasich, Pieter C. Dorrestein, Robert R. Dunn, Ashkaan K. Fahimipour, James Gaffney, Jack A. Gilbert, Grant Gogul, Jessica L. Green, Philip Hugenholtz, Greg Humphrey, Curtis Huttenhower, Matthew A. Jackson, Stefan Janssen, Dilip V. Jeste, Lingjing Jiang, Scott T. Kelley, Dan Knights, Tomasz Kosciolek, Joshua Ladau, Jeff Leach, Clarisse Marotz, Dmitry Meleshko, Alexey V. Melnik, Jessica L. Metcalf, Hosein Mohimani, Emmanuel Montassier, Jose Navas-Molina, Tanya T. Nguyen, Shyamal Peddada, Pavel Pevzner, Katherine S. Pollard, Gholamali Rahnavard, Adam Robbins-Pianka, Naseer Sangwan, Joshua Shorenstein, Larry Smarr, Se Jin Song, Timothy Spector, Austin D. Swafford, Varykina G. Thackray, Luke R. Thompson, Anupriya Tripathi, Yoshiki Vázquez-Baeza, Alison Vrbanac, Paul Wischmeyer, Elaine Wolfe, Qiyun Zhu, The American Gut Consortium, Allison E. Mann, Rob Knight

**Affiliations:** a Department of Pediatrics, University of California San Diego, La Jolla, California, USA; b Collaborative Mass Spectrometry Innovation Center, University of California, San Diego, La Jolla, California, USA; c Skaggs School of Pharmacy and Pharmaceutical Sciences, University of California, San Diego, La Jolla, California, USA; d Department of Computer Science and Engineering, University of California, San Diego, La Jolla, California, USA; e Department of Biology, San Diego State University, San Diego, California, USA; f Department of Applied Ecology, North Carolina State University, Raleigh, North Carolina, USA; g Biology and the Built Environment Center, University of Oregon, Eugene, Oregon, USA; h Department of Surgery, University of Chicago, Chicago, Illinois, USA; i Institute for Genomic and Systems Biology, University of Chicago, Chicago, Illinois, USA; j Department of Biosciences, Argonne National Laboratory, Chicago, Illinois, USA; k Marine Biology Laboratory, University of Chicago, Chicago, Illinois, USA; l Australian Centre for Ecogenomics, School of Chemistry and Molecular Biosciences, the University of Queensland, Brisbane, QLD, Australia; m Harvard T. H. Chan School of Public Health, Boston, Massachusetts, USA; n The Broad Institute of MIT and Harvard, Cambridge, Massachusetts, USA; o Department of Twin Research and Genetic Epidemiology, King’s College London, London, United Kingdom; p Departments of Psychiatry and Neurosciences, University of California San Diego, La Jolla, California, USA; q Sam and Rose Stein Institute for Research on Aging and Center for Healthy Aging, University of California San Diego, La Jolla, California, USA; r Department of Computer Science and Engineering, University of Minnesota, Minneapolis, Minnesota, USA; s Biotechnology Institute, University of Minnesota, Minneapolis, Minnesota, USA; t The Gladstone Institutes, University of California, San Francisco, California, USA; u Human Food Project, Terlingua, Texas, USA; v St. Petersburg State University, Center for Algorithmic Biotechnology, Saint Petersburg, Russia; w Department of Animal Science, Colorado State University, Fort Collins, Colorado, USA; x Department of Computational Biology, Carnegie Mellon University, Pittsburgh, Pennsylvania, USA; y Université de Nantes, Microbiotas Hosts Antibiotics and Bacterial Resistances (MiHAR), Nantes, France; z Department of Biostatistics, University of Pittsburgh, Pittsburgh, Pennsylvania, USA; aa Center for Microbiome Innovation, University of California, San Diego, La Jolla, California, USA; bb Department of Computer Science, University of Colorado Boulder, Boulder, Colorado, USA; cc California Institute for Telecommunications and Information Technology (Calit2), University of California San Diego, La Jolla, California, USA; dd Department of Obstetrics, Gynecology and Reproductive Sciences, University of California San Diego, La Jolla, California, USA; ee Ocean Chemistry and Ecosystems Division, Atlantic Oceanographic and Meteorological Laboratory, National Oceanic and Atmospheric Administration, stationed at Southwest Fisheries Science Center, La Jolla, California, USA; ff Department of Biological Sciences and Northern Gulf Institute, University of Southern Mississippi, Hattiesburg, Mississippi, USA; gg Department of Anesthesiology and Surgery, Duke University School of Medicine, Durham, North Carolina, USA; hh Duke Clinical Research Institute, Duke University School of Medicine, Durham, North Carolina, USA; University of Pennsylvania

**Keywords:** citizen science, microbiome

## Abstract

Although much work has linked the human microbiome to specific phenotypes and lifestyle variables, data from different projects have been challenging to integrate and the extent of microbial and molecular diversity in human stool remains unknown. Using standardized protocols from the Earth Microbiome Project and sample contributions from over 10,000 citizen-scientists, together with an open research network, we compare human microbiome specimens primarily from the United States, United Kingdom, and Australia to one another and to environmental samples. Our results show an unexpected range of beta-diversity in human stool microbiomes compared to environmental samples; demonstrate the utility of procedures for removing the effects of overgrowth during room-temperature shipping for revealing phenotype correlations; uncover new molecules and kinds of molecular communities in the human stool metabolome; and examine emergent associations among the microbiome, metabolome, and the diversity of plants that are consumed (rather than relying on reductive categorical variables such as veganism, which have little or no explanatory power). We also demonstrate the utility of the living data resource and cross-cohort comparison to confirm existing associations between the microbiome and psychiatric illness and to reveal the extent of microbiome change within one individual during surgery, providing a paradigm for open microbiome research and education.

**IMPORTANCE** We show that a citizen science, self-selected cohort shipping samples through the mail at room temperature recaptures many known microbiome results from clinically collected cohorts and reveals new ones. Of particular interest is integrating *n* = 1 study data with the population data, showing that the extent of microbiome change after events such as surgery can exceed differences between distinct environmental biomes, and the effect of diverse plants in the diet, which we confirm with untargeted metabolomics on hundreds of samples.

## Abstract (precis)

We show that a citizen science, self-selected cohort shipping samples through the mail at room temperature recaptures many known microbiome results from clinically collected cohorts and reveals new ones. Of particular interest is integrating *n* = 1 study data with the population data, showing that the extent of microbiome change after events such as surgery can exceed differences between distinct environmental biomes, and the effect of diverse plants in the diet, which we confirm with untargeted metabolomics on hundreds of samples.

## INTRODUCTION

The human microbiome plays a fundamental role in human health and disease. While many studies link microbiome composition to phenotypes, we lack understanding of the boundaries of bacterial diversity within the human population and the relative importance of lifestyle, health conditions, and diet to underpin precision medicine or to educate the broader community about this key aspect of human health.

**FIG 1:**
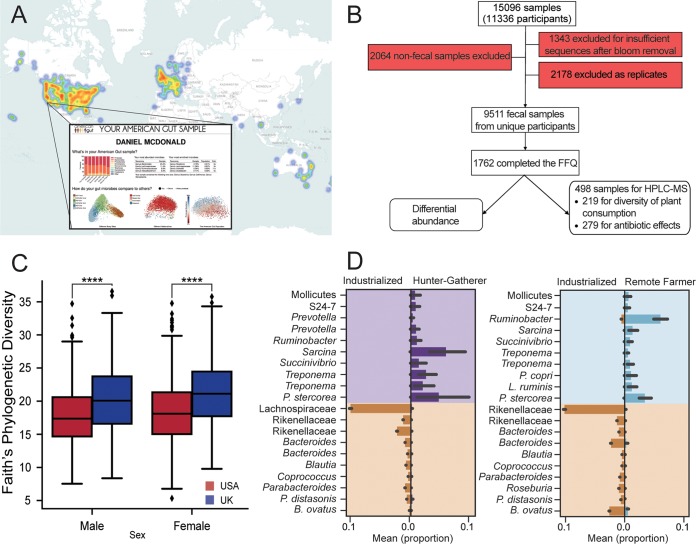
Population characteristics. (A) Participants across the world have sent in samples to American Gut, although the primary geographic regions of participation are in North America and the United Kingdom; the report that a participant receives is depicted. (B) The primary sample breakdown for subsequent analyses. Red denotes the reasons that samples were removed. (C) Between the two largest populations, the United States (*n* = 6,634) and the United Kingdom (*n* = 2,071), we observe a significant difference in alpha-diversity. (D) In a meta-analysis, the largely industrialized population that makes up American Gut exhibits significant differential abundances compared to nonindustrialized populations.

We launched the American Gut Project (AGP; http://americangut.org) in November 2012 as a collaboration between the Earth Microbiome Project (EMP) (1) and the Human Food Project (HFP; http://humanfoodproject.com/) to discover the kinds of microbes and microbiomes “in the wild” via a self-selected citizen-scientist cohort. The EMP characterizes global microbial taxonomic and functional diversity, and the HFP focuses on understanding microbial diversity across human populations. As of May 2017, the AGP included microbial sequence data from 15,096 samples from 11,336 human participants, totaling over 467 million (48,599 unique) 16S rRNA V4 gene fragments (abbreviated 16S). Our project informs citizen-scientist participants about their own microbiomes by providing a standard report (Fig. 1A) and deposits all deidentified data into the public domain on an ongoing basis without access restrictions (see Table S1 in the supplemental material). This reference database characterizes the diversity of the industrialized human gut microbiome on an unprecedented scale; reveals novel relationships with health, lifestyle, and dietary factors; and establishes the AGP resource and infrastructure as a living platform for discovery.

10.1128/mSystems.00031-18.6 TABLE S1  Summary of sample numbers and type in the other American Gut studies, sample distributions by country and territory, sample distributions by U.S. state, U.S. participant demographics, and per-sequencing-round sample accessions in EBI. Download TABLE S1, XLSX file, 0.6 MB. Copyright © 2018 McDonald et al. 2018 McDonald et al. This content is distributed under the terms of the Creative Commons Attribution 4.0 International license.

## RESULTS

### Cohort characteristics.

AGP participants primarily reside in the United States (*n* = 7,860). However, interest in the AGP rapidly expanded beyond the United States to the United Kingdom (*n* = 2,518) and Australia (*n* = 321), with 42 other countries or territories also represented (Fig. 1A; see also Table S1 in the supplemental material). Participants in the United States inhabit urban (*n* = 7,317), rural (*n* = 29), and mixed (*n* = 98) communities (2010 U.S. Census data based on participant ZIP codes) and span greater ranges of age, race, and ethnicity than other large-scale microbiome projects (2–6).

Using a survey modified from references 7 and 8, participants reported general health status, disease history, and lifestyle data (Table S2 and Text S1). In accordance with our institutional review board (IRB), all survey questions were optional (median per-question response, 70.9% [Table S2]). Additionally, 14.8% of participants completed a validated picture-based food frequency questionnaire (FFQ) (VioScreen; http://www.viocare.com/vioscreen.html), and responses correlated well with primary survey diet responses (Table S2).

10.1128/mSystems.00031-18.1 TEXT S1  Additional detail on the effect size comparisons, multicohort replication, projects using the American Gut Project infrastructure, and the American Gut Survey presented to participants. Download TEXT S1, DOCX file, 0.03 MB. Copyright © 2018 McDonald et al. 2018 McDonald et al. This content is distributed under the terms of the Creative Commons Attribution 4.0 International license.

10.1128/mSystems.00031-18.7 TABLE S2  American Gut data dictionary, proportion of responses per American Gut survey question that are represented as a single question (multiselection responses were omitted as these are stored in the metadata as per response type), informal dietary questions and correlations with the food frequency questionnaire, effect size results without bloom sOTUs, and variable mapping with reference 2. Download TABLE S2, XLSX file, 0.1 MB. Copyright © 2018 McDonald et al. 2018 McDonald et al. This content is distributed under the terms of the Creative Commons Attribution 4.0 International license.

We focused our primary investigative efforts on a “healthy adult” subset (*n* = 3,942) of individuals aged 20 to 69 years with body mass indexes (BMIs) ranging between 18.5 and 30 kg/m^2^; no self-reported history of inflammatory bowel disease (IBD), diabetes, or antibiotic use in the past year; and at least 1,250 16S sequences/sample (Fig. 1B and S1B).

The two largest populations in the data set (United States and United Kingdom) differed significantly in alpha-diversity, with Faith’s phylogenetic diversity (PD) higher in U.K. samples (9) (Mann-Whitney test *P* < 1 × 10^−15^) (Fig. 1C). One balance (10) (a log-ratio compositional transform) explained most of the taxonomic separation between U.S. and U.K. samples (area under the curve [AUC] = 77.7%; analysis of variance [ANOVA] *P* = 1.01 × 10^−78^, *F* = 386.85) (Fig. S1C and Table S3). To understand how these two populations differed from others, we compared adult AGP samples (predominantly from industrialized regions) to samples from adults living traditional lifestyles (6, 11, 12) (e.g., hunter-gatherer and remote agrarian populations). As previously observed (6), samples from industrial and traditional populations separated in principal-coordinate analysis (PCoA) space of unweighted UniFrac distances (13) (Fig. S1D). UniFrac is a formal distance metric (14) which computes a dissimilarity based on the amount of unique phylogenetic branch length between two samples. These distances show a greater variation within industrial populations than within traditional populations (2) and facile separation based on microbial taxonomy (industrial versus nonindustrial agrarian, AUC = 98.9%; ANOVA *P* = 1.52 × 10^−260^, *F* = 1,265.8; industrial versus hunter-gatherer, AUC = 99.5%; ANOVA *P* = 4.48 × 10^−227^, *F* = 1,092.35) (Fig. 1D and Table S3).

10.1128/mSystems.00031-18.2 FIG S1  Workflow and population-scale analyses. (A) Heat map of income levels from the U.S. Census and American Gut participant locations. (B) Sample flowchart for what sample sets correspond to each analysis. (C) Using PLS-DA, we observed separation between U.S. (*n* = 6,634) and U.K. (*n* = 2,071) fecal samples. (D) We performed a principal-coordinate analysis comparing children over the age of 3 years and adults from industrialized (*n* = 4,643 AGP samples, *n* = 4,927 samples total), remote farming (*n* = 131), and hunter-gatherer (*n* = 30) lifestyles. Download FIG S1, JPG file, 1.9 MB. Copyright © 2018 McDonald et al. 2018 McDonald et al. This content is distributed under the terms of the Creative Commons Attribution 4.0 International license.

10.1128/mSystems.00031-18.8 TABLE S3  sOTUs relevant to the balance analyses and summary of differentially abundant taxa in U.K. cohort (negative effect size indicated that the taxon is more prevalent in control [NC] subjects). Download TABLE S3, XLSX file, 0.2 MB. Copyright © 2018 McDonald et al. 2018 McDonald et al. This content is distributed under the terms of the Creative Commons Attribution 4.0 International license.

### Removal of bacterial blooms.

An important practical question is whether self-collected microbiome samples can match those from better-controlled studies. Most AGP samples are stools collected on dry swabs and shipped without preservative to minimize costs and avoid exposure to toxic preservatives. Escherichia coli and a few other taxa grow in transit, so based on data from controlled-storage studies as previously described (15), we removed sub-operational taxonomic units (sOTUs) (16) (median of 7.9% of sequences removed per sample) shown to bloom.

**FIG 2:**
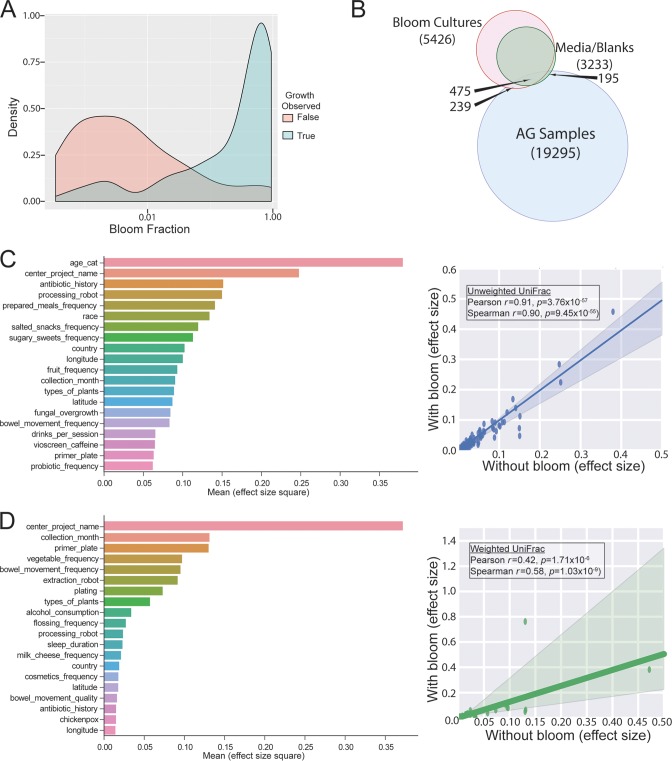
Blooms and effect sizes. (A) The fraction of 16S reads that recruit to bloom reads defined by Amir et al. (15) is strongly associated with the likelihood for microbial growth under aerobic culture conditions on rich medium. (B) Overlap of mass spectral features (consensus MS/MS cluster nodes; see Materials and Methods, “Molecular networking”) between AGP samples and blooms. (C) Unweighted UniFrac effect sizes. The inset shows the correlation of effect sizes when including or excluding the bloom 16S reads (Pearson *r* = 0.91, *P* = 3.76 × 10^−57^). (D) Weighted UniFrac effect sizes. The inset shows the correlation of the effect sizes when including or excluding bloom 16S reads (Pearson *r* = 0.42, *P* = 1.71 × 10^−6^); the outlier is the 16S bloom fraction of the sample.

We further characterized the impact of these organisms through culturing, high-performance liquid chromatography mass spectrometry (HPLC-MS) analysis of cultured isolates, and shotgun metagenomics of the primary samples and storage controls (15, 17). Culturing primary specimens stored at −80°C (United States, *n* = 116; United Kingdom, *n* = 73; other, *n* = 25) showed a strong correlation between the fraction of sequences reported as blooms in 16S sequencing and positive microbial growth following overnight incubation under aerobic conditions (Fig. 2A). Culture supernatants were characterized using HPLC-MS; most metabolites in these supernatants were absent from the primary specimens (Fig. 2B; see method details in Text S1). We sequenced draft genomes of 169 isolates; of these, 65 contained the exact E. coli 16S sequence in the published bloom filter (15). To characterize the impact of the 16S filter for blooms used exactly as described in reference 15, we computed effect sizes over the participant covariates and technical parameters for 9,511 individual participant samples, including and excluding blooms (complete list in Table S2), and observed tight correlations for both unweighted (Fig. 2C) (Pearson *r* = 0.91, *P* = 3.76 × 10^−57^; Spearman *r* = 0.90, *P* = 9.45 × 10^−55^) and weighted (Fig. 2D) (Pearson *r* = 0.42, *P* = 1.71 × 10^−6^; Spearman *r* = 0.58, *P* = 1.03 × 10^−9^) UniFrac values, suggesting that the presence of the blooms does not substantially alter effect sizes of the study variables. An outlier on the quantitative metric (weighted UniFrac) is present and corresponds to a variable representing the fraction of bloom reads in a sample. In Text S1, we further compare the ranking of these effect sizes to reference 17. The filter for 16S blooms acts by removing exact sOTUs from the data set prior to rarefaction. This filter is applied to all samples in the data set, including samples from other studies when performing meta-analyses.

### Novel taxa and microbiome configurations.

**FIG 3:**
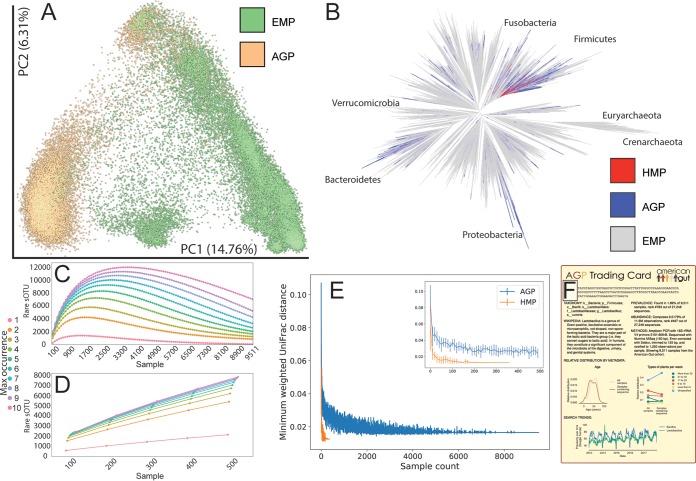
OTU and beta-diversity novelty. (A) The AGP data placed into the context of extant microbial diversity at a global scale. (B) A phylogenetic tree showing the diversity spanned by the AGP and the HMP in the context of Greengenes and the EMP. (C and D) sOTU novelty over increasing numbers of samples in the AGP (C); the AGP appears to have begun to reach saturation and is contrasted with the data from the work of Yatsunenko et al. (6) (D), which, unlike the AGP, had extremely deep sequencing per sample. (E) The minimum observed UniFrac distance between samples over increasing numbers of samples for the AGP and the HMP; the inset is from 0 to 500 samples. (F) An AGP “trading card” of an sOTU of interest (shown in full in Fig. S2).

To better understand human microbiome diversity, we placed AGP samples in the context of the EMP (1). Building on earlier work that revealed a striking difference between host-associated and environmental microbiomes (18), we found that the distances between pairs of human gut microbiomes (just one body site in one vertebrate) are often comparable to the distances between completely different types of environments and that even the first two dimensions of a PCoA plot capture this intuition visually (Fig. 3A). This intuition is confirmed by PERMDISP (homogeneity of dispersion) analysis: on average, a randomly chosen AGP fecal sample was more likely to be close to the centroid of the AGP fecal distribution (distances in the range of 0.1 to 0.2) than a randomly chosen EMP sample and was less distant on average from the centroid overall (*P* < 0.001, PERMDISP). However, the maximum distance from the centroid was greater for AGP than EMP (0.65 versus 0.58; no statistical analysis possible because this is a single value), matching the intuition from the PCoA plot that the dispersion of the AGP samples is large compared to individual environments and that the extremes of the spread are comparable to that of the EMP. Because the maximum distance from the centroid increases with the number of samples, this distance would have been expected to be greater for the EMP, which is a larger data set.

10.1128/mSystems.00031-18.3 FIG S2  Trading cards and LS’s samples compared to ICU patients and AGP participants and diet state change analysis. (A) Unweighted UniFrac distance distributions for the sample immediately prior to surgery versus all ICU fecal samples and distances of the sample immediately following surgery versus all ICU fecal samples (Kruskal-Wallis *H* = 79.774, *P* = 4.198 × 10^−19^). (B) Same as panel A except comparing against all AGP fecal samples (Kruskal-Wallis *H* = 8117.734, *P* = 0.0). (C) The median distances of each sample in LS’s longitudinal data set compared to both ICU and AGP. The last presurgery sample is on day 25, and the first postsurgery sample is day 27. (D) A principal-coordinate analysis of UniFrac distances of the American Gut Project, samples from the “extreme” diet study by David et al. (25), and the Earth Microbiome Project. No obvious state change by the diet of the participants in the work of David et al. is observed. Download FIG S2, JPG file, 1.5 MB. Copyright © 2018 McDonald et al. 2018 McDonald et al. This content is distributed under the terms of the Creative Commons Attribution 4.0 International license.

Inserting the sOTU fragments of AGP and EMP samples into a Greengenes (19) reference phylogenetic tree using SATé-enabled Phylogenetic Placement (SEPP) (20) (Fig. 3B) showed that the AGP population harbored much broader microbial diversity, as measured by phylogenetic diversity, than the Human Microbiome Project (HMP) (5). While the AGP vastly exceeds the phylogenetic diversity observed in the HMP (Faith’s PD, 1,579.6 versus 338.2), both data sets are dwarfed by the breadth of bacterial and archaeal phylogenetic diversity in environmental samples (Faith’s PD, 17,740.6). This result is expected based on the relative size of the data sets (HMP < AGP < EMP), as Faith’s PD increases with sampling effort until the diversity of a habitat is saturated, which has not yet been achieved for any of these types of samples. We confirmed that these differences were statistically significant (*P* < 0.001) by bootstrap resampling samples from each study, measuring the distances again, and examining the fraction of the time that the rank order of the PD of the studies differed from that reported (0 of 1,000 replicates). Examining sOTUs over increasing numbers of samples, we observed a reduction in the discovery rate of novel sOTUs starting around 3,000 samples, emphasizing the need for focused sampling efforts outside the present AGP population (Fig. 3C). The importance of sample size for detecting novel microbes and microbiomes is apparent when contrasted with the work of Yatsunenko et al. (6), which contained hundreds of samples from three distinct human populations at ~1 million sequences/sample (Fig. 3D). This effect is magnified in beta-diversity analysis, where the AGP has saturated the configuration space, and new samples are not “distant” from existing samples (Fig. 3E). To encourage broad scientific engagement with sOTUs found in the AGP, we adapted the EMP “trading cards” for sOTUs (Fig. 3F and S2).

### Temporal and spatial analyses.

**FIG 4:**
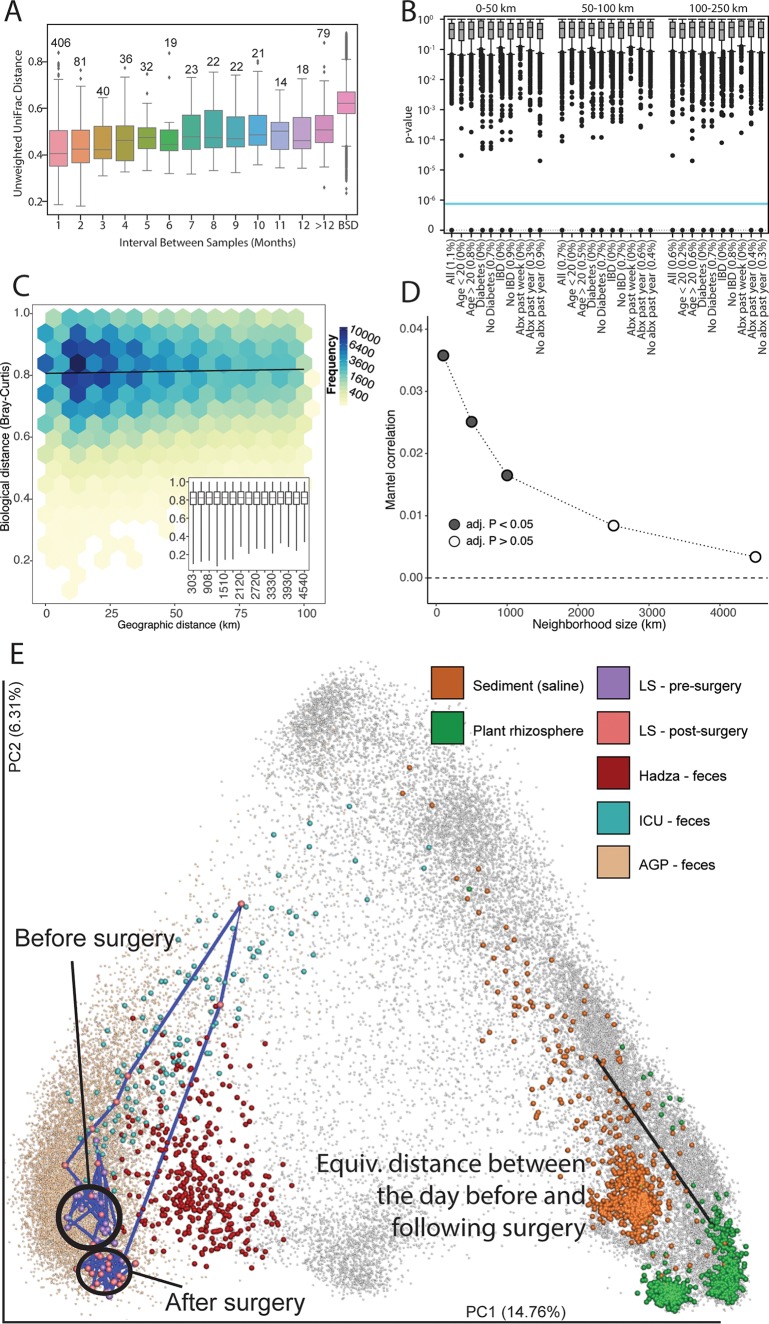
Temporal and spatial patterns. (A) Five hundred sixty-five individuals had multiple samples. Distances between samples within an individual shown at 1 month, 2 months, etc., out to over 1 year; between-subject distances are shown as BSD. Even at 1 year, the median distance between a participant’s samples is less than the median between-participant distance. (B) Within the United States, spatial processes of sOTUs appear driven by stochastic processes, as few sOTUs exhibit spatial autocorrelation (Moran’s *I*) on the full data set or partitions (e.g., participants older than 20 years). (C) Distance-decay relationship for Bray-Curtis dissimilarities between subject pairs that are within a 100-km (great-circle distance) neighborhood radius of one another (Mantel test *r* = 0.036, adjusted *P* = 0.03). To avoid the overplotting associated with visualization of the more than 3.4 × 10^5^ pairwise comparisons, we visualized this relationship using two-dimensional frequency bins; darker colors indicate higher-frequency bins. Solid lines represent fits from linear models to raw data. The inset shows the largest radius (i.e., the contiguous United States). Axes are the same as in the large panel. (D) Mantel correlogram of estimated Mantel *r* correlations, significance of distance-decay relationships, and neighborhood size (*x* axis). Filled points represent neighborhood sizes for which distance-decay relationships were significant (adjusted *P* values < 0.05). (E) Characterizing a large bowel resection using the AGP, the EMP, a hunter-gatherer population, and ICU patients in an unweighted UniFrac principal-coordinate plot. A state change was observed in the resulting microbial community. The change in the microbial community immediately following surgery is the same as the distance between a marine sediment sample and a plant rhizosphere sample.

Longitudinal samples are required for understanding human microbiome dynamics (21). We examined 565 individuals who contributed multiple samples and observed an increasing trend of intrapersonal divergence with time. Still, over time individuals resemble themselves more than others, even after 1 year (Fig. 4A).

Recent reports suggest that the microbes of human bodies (8), like those of homes (22), are influenced mostly by local phenomena rather than regional biogeography (23), and accordingly, we observed only weak geographic associations with sOTUs (Fig. 4B), no significant distance-decay relationships (Fig. 4C), and, with Bray-Curtis distance, only a weak effect at neighborhood sizes of ca. 100 km (Mantel *r* = 0.036, Benjamini-Hochberg adjusted *P* = 0.03) to 1,000 km (Mantel *r* = 0.016, Benjamini-Hochberg adjusted *P* = 0.03) (Fig. 4D).

We tested whether patterns in individual longitudinal sample sets could be better explained when placed in the context of the AGP by integrating samples collected from (i) a time series of 58 time points from one subject (designated LS), prior to and following a large bowel resection; (ii) two time points from 121 patients in an intensive care unit (ICU) (24); (iii) samples from the “extreme” diet study from the work of David et al. (25); and (iv) samples from the Hadza hunter-gatherers for additional context (22). Through the longitudinal sampling of LS, dramatic pre- and postmicrobial configuration changes that exceeded the span of microbial diversity associated with the AGP population were observed (Fig. 4E; animated in reference 26). Immediately after surgery, the subject’s samples more closely resembled those of ICU patients (Kruskal-Wallis *H* = 79.774, *P* = 4.197 × 10^−19^) (Fig. S2A to C) and showed a persistent state change upon return to the AGP fecal space. Remarkably, the UniFrac distance between the samples immediately prior to and following the surgery was almost identical to the distance between a marine sediment sample and a plant rhizosphere sample (unweighted UniFrac distance of 0.78). Furthermore, the observed state change in LS is not systematically observed in the extreme diet study (Fig. S2D) (PERMANOVA [permutational multivariate analysis of variance] not significant [NS] when controlling for individual). Despite extensive dietary shifts, these subjects do not deviate from the background AGP context.

### Dietary plant diversity.

**FIG 5:**
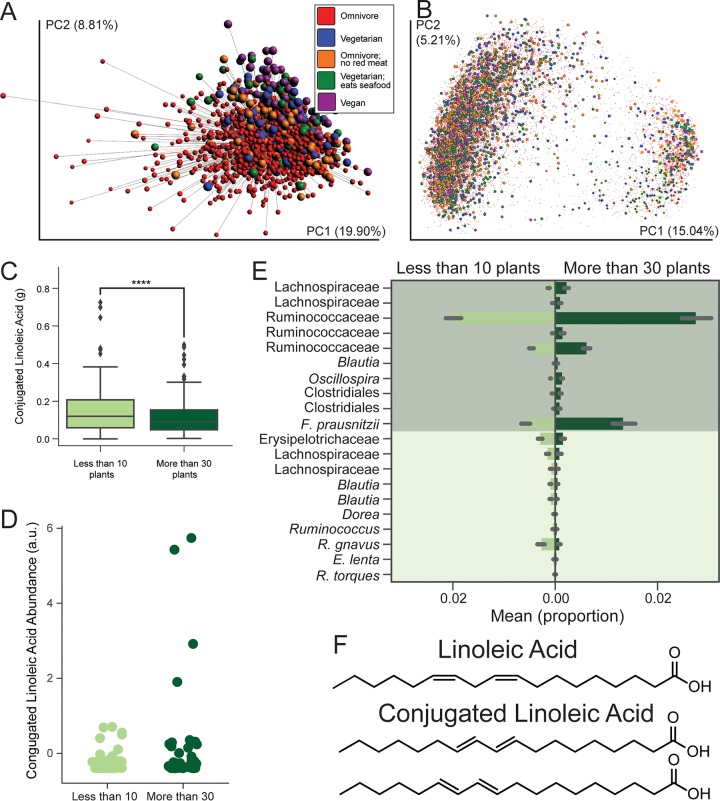
Diversity of plants in a diet. (A) Procrustes analysis of fecal samples from *n* = 1,596 individuals using principal components of the VioScreen FFQ responses and principal coordinates of the unweighted UniFrac distances (*M*^2^ = 0.988) colored by diet; Procrustes tests the fit of one ordination space to another. PCA shows grouping by diets such as vegan, suggesting that self-reported diet type is consistent with differences in micronutrients and macronutrients as recorded by the FFQ; however, these dietary differences do not explain relationships between the samples in 16S space. (B) The full AGP data set, including skin and oral samples, through unweighted UniFrac and principal-coordinate analysis, highlighting a lack of apparent clustering by diet type. (C and D) Dietary conjugated linoleic acid levels as reported by the FFQ between the extremes of plant diversity consumption (C) and the levels of CLA observed by HPLC-MS (D). (E) Differential abundances of sOTUs (showing the most specific taxon name per sOTU) between those who eat fewer than 10 plants per week and those who eat over 30 per week. (F) The molecules linoleic acid (LA) and conjugated linoleic acid (CLA) (only trans-, trans-isomers are shown) were found to comprise the octadecadienoic acid found to be the key feature in this difference in number of plants consumed.

The self-reported dietary data suggested, unexpectedly, that the number of unique plant species that a subject consumes is associated with microbial diversity, rather than self-reported categories such as “vegan” or “omnivore” (Fig. 2C and D). Principal-component analysis (PCA) of FFQ responses (Fig. 5A) revealed clusters associated with diet types such as “vegan.” However, these dietary clusters did not significantly relate to microbiome configurations (Fig. 5B) (Procrustes [Fig. 5A] *M*^2^ = 0.988). We therefore characterized the impact of dietary plant diversity, as measured using food frequency questionnaires and mass spectrometry (Fig. 5C and D), on the microbial community.

Using a partial least-squares (PLS) approach (10), we identified several putative short-chain fatty acid (SCFA) fermenters associated with eating more than 30 types of plants, including sOTUs putatively of the species Faecalibacterium prausnitzii and of the genus *Oscillospira* (27) (AUC = 68.5%; ANOVA *P* = 8.9 × 10^−39^, *F* = 177.2) (Fig. 5E and Table S3). These data suggest community-level changes associated with microbial fermentation of undigested plant components. Because bacteria differ in their carbohydrate binding modules and enzymes that hydrolyze diverse substrates in the gut (28), a diet containing various types of dietary fibers and resistant starches likely supports a more diverse microbial community (29, 30). Studies suggest that these types of responses in the gut microbiome to a high-plant diet may be common across vertebrates. For example, core fecal taxa of herbivorous mammals (both hindgut fermenters and ruminants) have been identified to include both *Prevotella* and *Ruminococcaceae* (31, 32). *Oscillospira* in particular has been found to increase in omnivorous lizards fed a plant-rich diet (33).

Plant consumption was also associated with a reduction in certain antibiotic resistance genes. Individuals who consume more than 30 types of plants per week compared to those who consume 10 or fewer plants per week had significantly reduced abundance of antibiotic resistance genes for aminoglycosides, chloramphenicol, and major facilitator superfamily (MFS) transporters (antibiotic efflux pumps).

To test these effects in the stool metabolome, we performed HPLC-MS annotation and molecular networking (34, 35) on a subset of fecal samples (*n* = 219), preferentially selecting individuals at the extremes of plant type consumption, i.e., eating <10 or >30 different types of plants per week. Several fecal metabolites differed between the two groups, with one key discriminating feature annotated as octadecadienoic acid (annotation level 2 according to the 2007 metabolomics initiative [36]). Further investigation using authentic standards revealed that the detected feature was comprised of multiple isomers, including linoleic acid (LA) and conjugated linoleic acid (CLA). CLA abundance did not correlate with dietary CLA consumption as determined by the FFQ (dietary [Fig. 5C]; Spearman *r* < 0.16; *P* > 0.15) but was significantly higher in individuals consuming >30 types of plants and those consuming more fruits and vegetables generally (Fig. 5D) (one-sided *t* test; *P* < 10^−5^). CLA is a known end product of LA conversion by lactic acid bacteria in the gut, such as Lactobacillus plantarum (37) and *Bifidobacterium* spp. (38). FFQ-based dietary levels of LA and MS-detected LA did not differ significantly between groups (Fig. S3), suggesting that their different microbiomes may differentially convert LA to CLA. Several other putative octadecadienoic acid isomers were also detected (Fig. 5F), some strongly correlated with plant consumption. Determining these compounds’ identities as well as their origin and function may uncover new links between the diet, microbiome, and health.

10.1128/mSystems.00031-18.4 FIG S3  Dietary levels of linoleic acid based on validated food frequency questionnaire responses; the linoleic acid detected by mass spectrometry did not differ significantly between groups consuming few and those consuming many types of plants per week. Download FIG S3, PDF file, 0.8 MB. Copyright © 2018 McDonald et al. 2018 McDonald et al. This content is distributed under the terms of the Creative Commons Attribution 4.0 International license.

### Molecular novelty in the human gut metabolome.

**FIG 6:**
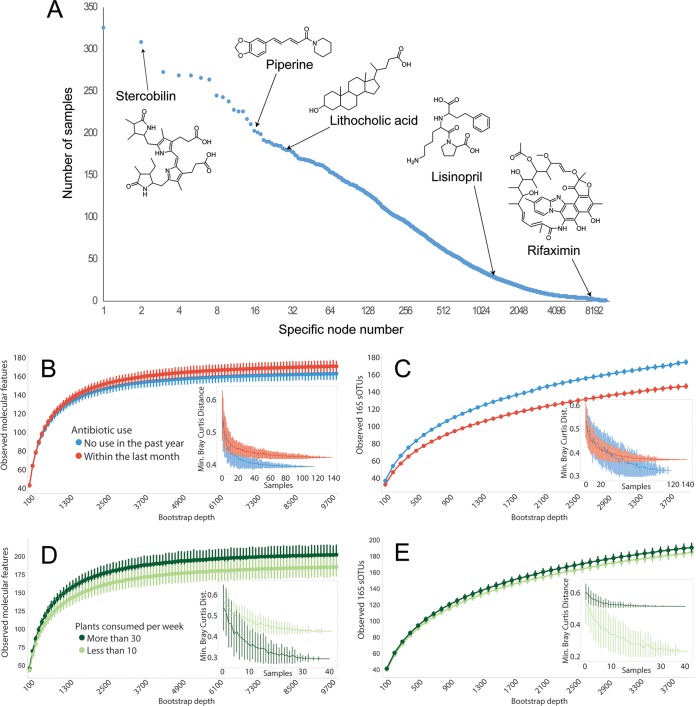
(A) Compound occurrence frequency plot. Examples of compounds originating from food (piperine, black pepper alkaloid), host (stercobilin, heme catabolism product), and bacterial activity (lithocholic acid, microbially modified bile acid) or exogenous compounds such as antibiotics (rifaximin) or other drugs (lisinopril, high blood pressure medication) are shown. (B to E) Alpha- and beta-diversity assessments of antibiotic (B and C) and plant (D and E) cohorts; insets depict minimum observed beta-diversity over increasing samples.

Our untargeted HPLC-MS approach allowed us to search for novel molecules in the human stool metabolome, parallel to our search for novelty in microbes and microbiome configurations described above. Bacterial N-acyl amides were recently shown to regulate host metabolism by interacting with G-protein-coupled receptors (GPCRs) in the murine gastrointestinal tract, mimicking host-derived signaling molecules (39). These agonistic molecules regulate metabolic hormones and glucose homeostasis as efficiently as host ligands. Manipulating microbial genes that encode enzymes that produce specific metabolites eliciting host cellular responses could enable new drugs or treatment strategies for many major diseases, including diabetes, obesity, and Alzheimer’s disease: roughly 34% of all marketed drugs target GPCRs (40). We observed N-acyl amide molecules previously hypothesized but unproven to be present in the gut (39) (Fig. 6 and Fig. S4), as well as new N-acyl amides (Fig. 6).

10.1128/mSystems.00031-18.5 FIG S4  Molecular novelty in the gut microbiome: molecular subnetwork of N-acyl amides (the full analysis can be found at https://gnps.ucsd.edu/ProteoSAFe/status.jsp?task=a07557dc26cc4d3f8a2076d5ae0898a2). The structural relationships in complex metabolite mixtures can be represented as networks, as described in Materials and Methods. The nodes denote unique metabolites common across multiple samples; the edges between nodes represent similarities of MS/MS spectra (the greater width of the edge denotes greater similarity); the edges are labeled with *m/z* differences of corresponding parent ions for the detected moieties. (A to I) Cluster/nodes of microbially derived G-protein-coupled receptor agonistic molecules detected in human fecal samples are shown. Molecules B, G, and H have been described previously (compounds 1, 2, and 4b [35] and commendamide [120]); molecules A, C, D, E, and I are previously not reported (proposed structures are shown). (J) Manual annotation of novel metabolites via comparison of experimental MS fragmentation patterns to those given in reference 39. (Top panel) Reference spectrum for compound 2 in reference 39; (bottom panel) experimental MS/MS spectrum for the parent ion *m/z* 611.5357. The compound is annotated as 3-(myristoyloxy)palmitoyl lysine. (K) *In silico* annotation using CSI:FingerID (81) for the ion with *m/z* 330.2640: the possible candidate structures are ranked by match percent. The top structure with 71.02% match corresponds to commendamide. (L) Manual annotation via comparison of experimental exact mass to that of the identified compound in reference 81, *N*-3-OH-palmitoyl ornithine. The peaks in the experimental MS/MS spectrum are examined and compared to theoretical fragments that would result from breaking bonds in the proposed structure. The structure is deemed to be consistent with the *N*-3-OH-palmitoyl ornithine annotation. Download FIG S4, PDF file, 1.3 MB. Copyright © 2018 McDonald et al. 2018 McDonald et al. This content is distributed under the terms of the Creative Commons Attribution 4.0 International license.

Levels of two N-acyl amides, annotated as commendamide (*m/z* 330.2635 [Fig. S4B]) and *N*-3-OH-palmitoyl ornithine (*m/z* 387.3220 [Fig. S4C]), positively correlated with a self-reported medical diagnosis of thyroid disease (Kruskal-Wallis false discovery rate [FDR] *P* = 0.032, *P* = 2.48 × 10^−3^, χ^2^ = 11.99; *N*-3-OH-palmitoyl ornithine, Kruskal-Wallis FDR *P* = 0.048, *P* = 5.63 × 10^−3^, χ^2^ = 10.35). Conversely, glycodeoxycholic acid (*m/z* 450.3187) was significantly higher in individuals not reporting thyroid disease diagnosis (Kruskal-Wallis FDR *P* = 1.28 × 10^−4^, *P* = 4.41 × 10^−7^, χ^2^ = 29.27). This cholic acid is produced through microbial dehydroxylation, again linking gut microbiota to endocrine function (41, 42).

Finally, we compared metabolome diversity to 16S rRNA amplicon diversity in the samples selected for dietary plant diversity and a second set of samples selected to explore antibiotic effects (*n* = 256 individuals who self-reported not having taken antibiotics in the past year [*n* = 117] or self-reported having taken antibiotics in the past month [*n* = 139]; participants were matched for age, BMI, and country). By computing a collector’s curve of observed molecular features in both cohorts (Fig. 6B and D), we observe that, paradoxically, individuals who had taken antibiotics in the past month (*n* = 139) had significantly greater molecular diversity (Kruskal-Wallis *H* = 255.240, *P* = 1.87 × 10^−57^) than those who had not taken antibiotics in the past year (*n* = 117) and differed in molecular beta-diversity (Fig. 6B, inset), suggesting that antibiotics promote unique metabolomes that result from differing chemical and microbial environments in the gut. Notably, the diversity relationships of this set are not reflected in 16S diversity (Fig. 6C and E), where antibiotic use shows decreased diversity (Kruskal-Wallis *H* = 3,983.839, *P* = 0.0). Within the dietary plant diversity cohort, we observed a significant increase (Kruskal-Wallis *H* = 897.106, *P* = 4.17 × 10^−197^) in molecular alpha-diversity associated with a high diversity of plant consumption (*n* = 42) compared to low plant diversity (*n* = 43), a relationship also observed in 16S diversity, where high dietary plant diversity increased 16S alpha-diversity (Kruskal-Wallis *H* = 65.817, *P* = 4.947 × 10^−16^). Recent antibiotic use (within the last 30 days) was, counterintuitively, associated with a decrease in quinolone resistance genes (see Materials and Methods), although not with a change in other families of antimicrobial resistance (AMR) genes. However, these results are difficult to interpret given the wide range of antibiotics taken by subjects, the many confounding variables, and the self-reported nature of the data. Studies in more carefully controlled clinical environments would be needed to make more meaningful statements about the role of the use of specific antibiotics in modifying the overall antimicrobial resistance profile of the human gut microbiome.

### Citizen science aspects of the project.

The AGP engages citizen scientists both through providing an individualized report (Fig. 1A) and through auxiliary resources to support human microbiome research, including an online course (Gut Check: Exploring Your Microbiome, https://www.coursera.org/learn/microbiome). Because the AGP is crowdsourced and self-selected, and subjects generally support the cost of sample processing, the population is unrepresentative in several important respects, including having a lower prevalence of smoking and obesity and having higher education and income (Fig. S1A) and underrepresentation of Hispanic and African American communities (Table S1); generalization of the results should therefore be treated with caution. Targeted and population-based studies will be crucial for filling these cohort gaps (Text S1). Because of the citizen science nature of the project, we sought to minimize errors and misclassifications well known to occur in self-reported data (43). Survey responses relied on controlled vocabularies. For analyses, we trimmed numeric entries at extremes (e.g., weight over 200 kg or below 2.5 kg) and excluded obviously incorrect answers (e.g., infants drinking alcohol) and samples for which necessary data were not supplied (e.g., missing ZIP code data for spatial analyses); see the supplemental material for details.

To promote public data engagement, we aimed to broaden the citizen science experience obtained by participating in AGP by “gamifying” the data and separately by developing an online forum for microbiome data discussion and discovery. The gamification introduces concepts of beta-diversity and challenges users to identify clusters of data in principal-coordinate space (http://csb.cs.mcgill.ca/colonyb/). The forum, called Gut Instinct (http://gutinstinct.ucsd.edu), enables participants to share lifestyle-based insights with one another. Participants also have the option to share their AGP sample barcodes, which will help us uncover novel contextual knowledge. Gut Instinct now has over 1,050 participants who have collectively created over 250 questions. Participants will soon design and run their own investigations using controlled experiments to further understand their own lifestyle and the AGP data.

### A living data set.

The AGP is dynamic, with samples arriving from around the world daily. This allows a living analysis, similar to continuous molecular identification and annotation revision in the Global Natural Products Molecular Networking (GNPS) database (34). Although the analysis presented here represents a single snapshot, samples continued to arrive during preparation of the manuscript. For example, after we defined the core “healthy” sample set, an exploratory analysis using matched controls was performed by collaborators to test for correlations between mental illness and microbiome composition (as reported in references 44 and 45). By analyzing mental illness status (depression, schizophrenia, posttraumatic stress disorder [PTSD], and bipolar disorder—four of the most disabling illnesses per the World Health Organization [46]) reported by AGP participants (*n* = 125) against matched 1:1 healthy controls (*n* = 125), we observed a significant partitioning using PERMANOVA in weighted UniFrac (*P* = 0.05, pseudo-*F* = 2.36). These findings were reproducible within U.S. residents (*n* = 122, *P* = 0.05, pseudo-*F* = 2.58), U.K. residents (*n* = 112, *P* = 0.05, pseudo-*F* = 2.16), women (*n* = 152, *P* = 0.04, pseudo-*F* = 2.35), and people 45 years of age or younger (*n* = 122, *P* = 0.05, pseudo-*F* = 2.45). We also reproduced some previously reported differentially abundant taxa in Chinese populations using our U.K. subset (44, 47) (Table S3). This shows that multicohort replication is possible within the AGP (additional detail in Text S1).

## DISCUSSION

The AGP provides an example of a successful crowdfunded citizen science project that facilitates human microbiome hypothesis generation and testing on an unprecedented scale, provides a free data resource derived from over 10,000 human-associated microbial samples, and both recaptures known microbiome results and yields new ones. Ongoing living data efforts, such as the AGP, will allow researchers to document and potentially mitigate the effects of a slow but steady global homogenization driven by increased travel, life spans, and access to similar diets and therapies, including antibiotics. Because the AGP is a subproject of the EMP (1), all samples were processed using the publicly available and widely used EMP protocols to facilitate meta-analyses, as highlighted above. Further examples of applications include assessing the stability of AGP runs over time and comparing the AGP population to fecal samples collected from a fecal transplant study (48) and an infant microbiome time series (49), the latter using different DNA sequencing technology, to highlight how this context can provide insight (50).

A unique aspect of the AGP is the open community process of assembling the Research Network and analyzing these data, which are released immediately on data generation. Analysis details are shared through a public forum (GitHub, https://github.com/knightlab-analyses/american-gut-analyses). Scientific contributions to the project were made through a geographically diverse Research Network represented here as the American Gut Consortium, established prior to project launch and which has grown over time. This model allows a “living analysis” approach, embracing new researchers and analytical tools on an ongoing basis (e.g., Qiita [http://qiita.microbio.me] and GNPS [34]). Examples of users of the AGP as a research platform include educators at several universities, UC San Diego Athletics, and the American Gastroenterological Association (AGA). Details on projects using the AGP infrastructure can be found in the supplemental material.

The AGP therefore represents a unique citizen science data set and resource, providing a rich characterization of microbiome and metabolome diversity at the population level. We believe that the community process for involving participants from sample collection through data analysis and deposition will be adopted by many projects harnessing the power of citizen science to understand the world around and within our own bodies.

## MATERIALS AND METHODS

### Participant recruitment and sample processing.

Participants signed up for the project through Indiegogo (https://www.indiegogo.com) and later FundRazr (http://fundrazr.com/). A contribution to the project was made to help offset the cost of sample processing and sequencing (typically $99 per sample; no requirement to contribute if another party was covering the contribution). All participants’ consent was obtained under an approved Institutional Review Board human research subject protocol, either from the University of Colorado Boulder (protocol no. 12-0582; December 2012 to March 2015) or from the University of California, San Diego (protocol no. 141853; February 2015 to present). The IRB-approved protocol specifically allows for public deposition of all data that are not personally identifying and for return of results to participants (Fig. 1A). This research was performed in accordance with the University of Colorado Boulder’s Institutional Review Board protocol number 12-0582 and the University of California San Diego’s Human Research Protection Program protocol number 141853.

Self-reported metadata were collected through a web portal (http://www.microbio.me/americangut). Samples were collected using BBL culture swabs (Becton, Dickinson and Company, Sparks, MD) and returned by mail. Samples collected in Australia and the United Kingdom were shipped using domestic post within each country to an aggregation site and stored at −80°C at the aggregation site until shipment to the United States. Shipment to the United States was done on dry ice using a certified shipping service. All samples were handled and processed in kind with other American Gut samples. For more information, please find an extensive benchmark of shipping conditions in reference 17. All samples were processed using the EMP protocols. Briefly, the V4 region of the 16S rRNA gene was amplified with barcoded primers and sequenced as previously described (51). Sequencing prior to August 2014 was done using the 515f/806r primer pair with the barcode on the reverse primer (52); subsequent rounds were sequenced with the updated 515f/806rB primer pair with the barcode on the forward read (52). Sequencing batches 1 to 19 and 23 to 49 were sequenced using an Illumina MiSeq; sequencing for batches 20 and 21 was performed with an Illumina HiSeq Rapid Run, and round 22 was sequenced with an Illumina HiSeq High-Output.

### 16S data processing.

The 16S sequence data were processed using a sequence variant method, Deblur v1.0.2 (16), trimming to 125 nucleotides (nt) (otherwise default parameters), to maximize the specificity of 16S data; a trim of 125 nt was used because one sequencing round in the American Gut used 125 cycles while the rest used 150. Following processing by Deblur, previously recognized bloom sequences were removed (15). The Deblur sOTUs were inserted into the Greengenes 13_8 (19) 99% reference tree using SEPP (20). SEPP uses the simultaneous alignment and tree estimation strategy described in reference 53 to identify reasonable placements for sequence fragments within an existing phylogeny and alignment. Taxonomy was assigned using an implementation of the RDP classifier (54) as implemented in QIIME2 (55). Multiple rarefactions were computed, with the minimum being 1,250 sequences per sample with the analyses using the 1,250-sequence set except where noted explicitly. Rarefaction was used to mitigate uneven sequencing depth in accordance with the benchmarking in reference 56. Diversity calculations were computed using scikit-bio 0.5.1 with the exception of UniFrac (13), which was computed using an unpublished algorithmic variant, Striped UniFrac (https://github.com/biocore/unifrac), which scales to larger data sets and produces results identical to previously published UniFrac algorithms. In brief, unweighted UniFrac computes a dissimilarity between two samples by summing up the amount of branch length that is unique to each sample and divides this by the sum of the branch length that is common to the two samples.

### Metadata curation.

To address the self-reported nature of the AGP data and the ongoing nature of the project, basic filtering was performed on the age, height, weight, and body mass index (BMI). Height and weight were gated to consider heights only between 48 cm and 210 cm and weight between 2.5 kg and 200 kg. BMI calculations using values outside this range were not considered. We assumed that age was misreported by any individual who reported a birthdate after the sample was collected. We also assumed that age was misreported for participants who reported an age of less than 4 years but height over 105 cm, weight over 20 kg, or any alcohol consumption. Values assumed to be incorrect were dropped from analyses (see Fig. S1B in the supplemental material).

### Sample selection.

Analyses in this paper were performed on a subset of the total AGP samples. A single fecal sample was selected for each participant with at least one fecal sample that amplified to 1,250 sequences per sample unless otherwise noted. Priority was given to samples that were associated with VioScreen (http://www.viocare.com/vioscreen.html) metadata.

The samples used for analysis and subsets used in various analyses are described in Table S2. Briefly, we defined the healthy subset (*n* = 3,942) as adults aged 20 to 69 years with a BMI between 18.5 and 30 kg/m^2^ who reported no history of inflammatory bowel disease (IBD) or diabetes and no antibiotic use in the last year. There were 1,762 participants who provided results for the VioScreen food frequency questionnaire (FFQ; http://www.viocare.com/vioscreen.html). The meta-analysis with non-Western samples (*n* = 4,643) included children over the age of 3 years and adults with a BMI of between 18.5 and 30 kg/m^2^ and no reported history of inflammatory bowel disease, diabetes, or antibiotic use in the last year.

### Population-level comparisons.

Population-level comparisons were calculated for all American Gut participants living in the United States. BMI categorization was considered only for adults over the age of 20 years, since the description of BMI in children is based on their age and sex. Education level was considered for adults over the age of 25 years. This threshold was used to match the available data from the U.S. Census Bureau (57). The percentage of the American Gut participants was calculated as the fraction of individuals who reported results for that variable. U.S. population data are from the 2010 census (58); U.S. Census Bureau reports (57); Centers for Disease Control reports on obesity (59), diabetes (60), inflammatory bowel disease (IBD) (61), and smoking (62); and a report from the Williams Institute (63) (Table S2).

### Alpha- and beta-diversity analyses within American Gut.

OTU tables generated in the primary processing step were rarefied to 1,250 sequences per sample. Shannon, observed OTU, and PD whole-tree diversity metrics were calculated as the mean of 10 rarefactions using QIIME (9, 55). Alpha-diversity for single metadata categories was compared with a Kruskal-Wallis test. Unweighted UniFrac distance between samples was tested with PERMANOVA (64) and permuted *t* tests in QIIME.

### Balances.

The goal of this analysis was to design two-way classifiers to classify samples and sOTUs. This will allow us to identify sOTUs that are strongly associated with a given environment. To do this while accounting for issues due to compositionality, we used balances (10) constructed from partial least-squares (65).

First, the sOTU table was centered log ratio (CLR) transformed with a pseudocount of 1. Partial least-squares discriminant analysis (PLS-DA) was then performed on this sOTU table using a single PLS component, using a binary categorical variable as the response and the CLR-transformed sOTU table as the predictor. This PLS component represented an axis, which assigns scores to each OTU according to how strongly associated it is with each class. An sOTU with a strong negative score indicates an association for the one category, which we will designate the negative category. An sOTU with a strong positive score indicates that sOTU is strongly associated with the other category, which we will designate the positive category.

We assumed that PLS scores associated with each OTU were normally distributed. Specifically

$$\text{score}\left(x_{\text{pos}}^{\left(i\right)}\right)∼N\left(\mu_{\text{pos}},\sigma_{\text{pos}}^{2}\right)$$

$$\text{score}\left(x_{\text{neg}}^{\left(i\right)}\right)∼N\left(\mu_{\text{neg}},\sigma_{\text{neg}}^{2}\right)$$

$$\text{score}\left(x_{\text{null}}^{\left(i\right)}\right)∼N\left(\mu_{\text{null}},\sigma_{\text{null}}^{2}\right)$$

where μ_null_ ≈ 0 , μ_neg_ < 0, and μ_pos_ > 0. To obtain estimates of these normal distributions, Gaussian mixture models with three Gaussians were fitted from the PLS scores. Thresholds were determined from the intersection of Gaussians. The OTUs with PLS scores less than the intersection *N*(μ_null_, σ_null_^2^) and *N*(μ_neg_, σ_neg_^2^) are classified as associated with the negative category. The OTUs with PLS scores greater than the intersection *N*(μ_null_, σ_null_^2^) and *N*(μ_pos_, σ_pos_^2^) are classified as associated with the positive category.

The balance was constructed as follows

$$b=\sqrt{\frac{\left|x_{\text{pos}}\right|\left|x_{\text{neg}}\right|}{\left|x_{\text{pos}}\right|+\left|x_{\text{neg}}\right|}}\log\left(\frac{g\left(x_{\text{pos}}\right)}{g\left(x_{\text{neg}}\right)}\right)$$

From this balance, we calculated receiver operator characteristic (ROC) curves and AUC to assess the classification accuracy and ran ANOVA to assess the statistical significance. The dimensionality was shrunk through some initial filtering (an sOTU must have at least 50 reads, must exist in at least 20 samples except where noted, and must have a variance over 10 to remove sOTUs that do not appear to change), so that the number of samples is greater than the number of sOTUs to reduce the likelihood of overfitting. This technique was used to investigate differences due to plant consumption, country of residence, and Western versus non-Western samples and was consistently applied with the exception that a filter of 5 samples was used for the Western–versus–non-Western analysis due to group sample sizes.

Balances on plant consumption were constructed using partial least-squares. Only samples from people who consumed fewer than 10 types of plants a week or more than 30 types of plants a week were considered.

### Meta-analysis of samples from the American Gut and from individuals living agrarian and hunter-gatherer lifestyles.

A meta-analysis compared fecal samples collected from healthy individuals who were 3 years of age or older and included in the AGP data set to a previously published 16S rRNA V4 region data set that included healthy people living an industrialized, remote agrarian, or hunter-gatherer lifestyle (6, 11, 12). The AGP subset of healthy individuals was determined by filtering by the metadata columns “subset_antibiotic,” “subset_ibd,” and “subset_diabetes” and, for individuals over the age of 16 years, “subset_bmi.” All data sets were processed using the Deblur pipeline as noted above, with the exception that all reads in the meta-analysis, including AGP data, were trimmed to 100 nt to accommodate the read length in the work of Yatsunenko et al. (6). Bloom reads as described above were removed from all samples. We used Striped UniFrac as noted above to estimate beta-diversity (unweighted UniFrac) and EMPeror software (66) version 0.9 to visualize principal coordinates. We used a nonparametric PERMANOVA with 999 permutations to test for significant differences in fecal microbiomes associated with industrialized, remote agrarian, and hunter-gatherer lifestyles. All AGP samples were considered to be from people living an industrialized lifestyle. Balances were constructed from partial least-squares to assess the differences between the hunter-gatherer and industrialized populations and between the remote farmers and industrialized populations.

### Spatial autocorrelation.

We sought to investigate distance-decay patterns—the relationship between microbial community similarity and spatial proximity—among American Gut participants, to determine the extent to which geographical distances could explain variation in microbial community taxonomic compositions between participant pairs. The correlation between community-level Bray-Curtis (67) distances and participants’ spatial proximities (i.e., great-circle distances, kilometers) was assessed using a Mantel test (68) with 1,000 matrix permutations. Analyses were conducted using the subset of participants located in the continental United States who had not received antibiotics in the last year. Different neighborhood sizes were investigated in order to detect the relevant spatial scale on which significant distance-decay patterns in microbial community compositions emerged. To accomplish this, we computed distance-decay relationships for a series of model adjacencies corresponding to neighborhood radii of 100, 500, 1,000, 2,500, and 4,500 km among participants and adjusted *P* values for multiple comparisons using the Benjamini-Hochberg procedure (69). We also studied spatial correlations in phylogenetic community dissimilarities, calculated as weighted normalized UniFrac distances, using the procedure described above. Analyses were conducted in the R statistical programming environment.

The spatial autocorrelation of each individual taxon was assessed using Moran’s *I* statistic (70). Taxa present in fewer than 10 samples were filtered, since these would not be sufficiently powered. Analyses were conducted using binary spatial weight matrices, with neighborhoods of 0 to 50 km, 50 to 100 km, and 100 to 250 km. The different neighborhoods were useful for detecting spatial autocorrelation at different scales. All spatial weight matrices were row standardized. We checked for spatial autocorrelation at three taxonomic ranks: class, genus, and OTU. We also considered whether there was autocorrelation within subsets of individuals who were under 20 years old and between 20 and 70 years old; those having IBD, no IBD, diabetes, and no diabetes; and those who had taken antibiotics within the past week or year or not within the past year. The results presented above did not qualitatively depend on the subset of individuals considered. Statistical significance was assessed using permutation tests, which were implemented using a Markov chain Monte Carlo algorithm. To assess each *P* value, 100 chains were run each starting from a different random permutation. Each chain had 1,000 iterations. We used Bonferroni corrections to correct for multiple comparisons, with an overall significance level set to 0.05. Analyses were run using custom Java code, optimized for running many spatial autocorrelation analyses on large data sets (71).

### Metadata cross-correlation.

To account for covariance among metadata for effect size and variation analyses, we examined the correlation between individual metadata variables, including technical parameters. Groups in ordinal variables were combined if there was insufficient sample size (e.g., people who reported sleeping less than 5 h were combined with those who reported sleeping 5 to 6 h into a variable described as “less than 6”). The same transformations were used for effect size analysis. Any group with fewer than 25 total observations was ignored during analysis; if this resulted in a metadata column having no groups, the column was removed from analysis. The relationship between continuous and ordinal covariates was calculated using Pearson’s correlation. Ordinal and categorical covariates were compared using a modified Cramér *V* statistic (72). Continuous and categorical covariates were compared with a Welch *t* test (72). We used 1 −* R* as a distance between the covariates. Traversing the resulting binary, weighted cluster tree starting at tip level into the direction of the root, i.e., bottom up, we grouped tips together that are members of the same subtree after covering a distance of approximately 0.5 (branch length 0.29). A representative variable from each cluster was selected for analysis (Table S2).

### Effect size calculations.

Effect size was calculated on 179 covariates (including technical parameters), selected from the cross correlation (Table S2). Ordinal groups with small sample sizes at the extreme were collapsed as noted above. Individuals who reported self-diagnosis or diagnosis by an alternative practitioner for medical conditions were excluded from the analysis. Any metadata variable with fewer than 50 observations per group or that made up less than 3% of the total number of respondents was also excluded from the effect size analysis. Continuous covariates were categorized into quartiles. For each one of the 179 variables, we applied the mixed directional FDR (mdFDR) (73) methodology to test for the significance of each pairwise comparison among the groups. For each significant pairwise comparison, we computed the effect size using Cohen’s *d* (74) or the absolute difference between the mean of each group divided by the pooled standard deviation. For analysis of diversity, we used Faith’s phylogenetic diversity (alpha-diversity) and weighted and unweighted UniFrac distances (beta-diversity).

### Variation analysis.

Using the methodology reported in the supplemental material of reference 4, we computed Adonis (75) using 1,000 permutations, over the sample sets used in the effect size calculations as noted above, and applied Benjamini-Hochberg correction (FDR < 0.1) to assess drivers of variation in beta-diversity.

### Meta-analysis movie.

American Gut samples from all body sites were combined with data from an infant time series (ITS) (49), a fecal transplant study (44), and recent work characterizing the microbiome of patients in the intensive care unit (24). The combination of the data sets in the figshare supplementary movie S2 in reference 50 required that all sequences were trimmed to an even length of 125 nucleotides. All projects except for the infant time series were sequenced using an Illumina instrument. In order to combine the data, we expressed the Illumina and non-Illumina data through a common reference database. Specifically, the Deblur sOTUs from the Illumina data were mapped against the Greengenes (19) database (13_8 release) using 99% similarity; the associations between the input sOTUs, and their cluster memberships, were used to construct an OTU table based on the original sOTU-per-sample sequence counts (i.e., summing the counts for all sOTUs in a common OTU). The infant time series data were picked using a closed reference OTU picking approach against the same reference at the same similarity. The infant time series data set followed a closed reference OTU picking approach using 99% similarity. The resulting two tables (from Illumina-generated data and the ITS data set) were merged and analyzed using the Greengenes 99% tree. The table was rarefied to 1,250 sequences per sample. Principal-coordinate projections were calculated based on unweighted UniFrac distance (13). The principal-coordinate analysis was visualized and animated in EMPeror 1.0.0-beta8-dev (66, 76). The movie was captured in QuickTime (Apple, Cupertino, CA) and edited with Premiere Pro (Adobe, San Jose, CA).

### Integration with the Earth Microbiome Project.

A precomputed 100-nt Deblur BIOM table representing the data in reference 1 was obtained from ftp://ftp.microbio.me/emp/release1/otu_tables/deblur/. One-hundred-nucleotide (100-nt) Deblur tables were also obtained from Qiita for Hadza fecal samples (Qiita study identifier [ID] 11358 [22]), ICU microbiome samples (Qiita study ID 2136 [24]), and a longitudinal series which includes samples immediately prior to and following a large bowel resection (Qiita study ID 10283, EBI accession no. ERP105968, unpublished); all samples were processed using the EMP Illumina 16S V4 protocol. The EMP data set used a minimum sOTU count of 25; the same threshold was applied to the other data sets included prior to merge. Blooms as identified by the method in reference 15 were removed from all samples. This collection of BIOM tables was then merged, yielding an OTU table representing 40,600 samples. sOTUs were restricted to those already present in the EMP 100-nt fragment insertion tree, which represents 329,712 sOTUs. The table was then rarefied to 1,000 sequences per sample, and unweighted UniFrac was computed using 768 processors with the aforementioned Striped algorithm. Visualizations and animations were performed using EMPeror v1.0.0b12.dev0.

### Extreme diet study state assessment.

The sequence data from reference 25 were processed by Deblur to assess 16S sOTUs in common with the AGP processing above. In order to assess a state difference with PERMANOVA, we needed to control for sample independence within the longitudinal sampling. To do so, we randomly selected one sample from each individual per diet, computed PERMANOVA, and repeated the process 100 times. None of the trials produced a *P* value below 0.05.

### VioScreen PCA and diet type Procrustes analysis.

Before performing principal-component analysis (PCA) on the informal diet questions, VioScreen variables that were categorical or received less than a 90% response among the 1,762 participants were excluded, leaving 1,596 participants. PCA was then performed using the VioScreen information from these participants’ responses over 207 VioScreen questions and then colored by their types of diet as answered in the AGP informal food survey. The coordinates from the PCA were extracted. For the same samples, PCoA of unweighted UniFrac distances was computed on the 16S data subset from the primary processing set. The coordinates from the PCA and the PCoA were assayed for a measure of fitness using Procrustes as implemented in QIIME v1.9.1.

### Beta-diversity added.

To assess added beta-diversity, we applied the technique used in reference 5 (Fig. 3). Specifically, we randomly sampled *n* samples from the distance matrix 10 times, over an increasing value of *n*. For each set of sampled distances, we computed the minimum observed distance.

### sOTU novelty.

To assess sOTU novelty, we randomly sampled *n* samples from an sOTU table 10 times, over an increasing value of *n*. At each sampling, we computed the number of sOTUs observed with read counts within minimum thresholds. In other words, a minimum threshold of 1 is the number of singletons observed in the sampled set, a minimum threshold of 2 is the number of singletons and doubletons, etc.

### Within-individual beta-diversity.

Many of the individuals in the American Gut Project contributed multiple samples but at uneven time intervals. In order to explore intrapersonal variation, we replicated the analysis in the work of Lloyd-Price et al. (77) (Fig. 3). Specifically, we determined all time deltas between a subject’s samples and gathered the distributions of beta-diversity between any two samples binned by month. An individual is represented only a single time in a given month but may be represented in multiple months if the individual had, for instance, contributed samples over the course of a year.

### HPLC-MS analysis.

A total of 498 samples were selected for analysis via mass spectrometry. Specifically, two groups were chosen. First, given the large body of primary literature describing the negative impact of antibiotics on the gut microbiome, and the general interest in this topic from many American Gut participants, we chose 279 samples from individuals (age, BMI, and country matched) who self-reported not having taken antibiotics in the past year or reported having taken antibiotics in the past month or week. We chose a second group of 219 samples collected from individuals who answered the question “In an average week, how many different plants do you eat? (e.g., if you consume a can of soup that contains carrots, potatoes, and onion, you can count this as 3 different plants; if you consume multigrain bread, each different grain counts as a plant. Include all fruits in the total)” on the main American Gut Project main survey and who had also completed the VioScreen food frequency questionnaire. When American Gut participants collect samples, they do so on a double-headed swab; therefore, all samples chosen for this analysis had one remaining swab head (the first had been used for DNA extraction and microbiome sequencing).

### Cell culture sample preparation for metabolomics analysis.

The supernatants collected from cell cultures (see “Expanded bloom assessment” below) were processed to make them compatible with HPLC-MS analysis. The solid-phase extraction (SPE) with wash was carried out to reduce the impact of cell culture medium, which is highly detrimental for the electrospray ionization (ESI). The 30-mg sorbent Oasis HLB (Waters, Waltham, MA) SPE cartridges were used to achieve broad metabolite coverage. The cell samples were stored at −80°C and thawed at room temperature immediately prior to extraction. The thawed samples were then centrifuged for 10 min at 1,200 rpm and extracted. For the SPE, the Oasis HLB SPE cartridge was conditioned with 700 µl of 100% HPLC-grade methanol and equilibrated with 700 µl of HPLC-grade deionized (DI) water. The cell supernatant (~350 to 400 µl) was loaded into the cartridge and allowed to slowly elute. The loaded SPE wells were then washed with 800 µl of 5% methanol in water, and the absorbed material was slowly eluted with 200 µl of 100% methanol. Vacuum up to ~20 lb/in^2^ was applied for the wells that did not elute within an hour. The collected eluent was stored at −20°C until the HPLC-MS analysis.

### Fecal sample preparation for metabolomics analysis.

The swab tubes scheduled for analysis were removed from the −80°C freezer and placed on dry ice for the duration of sample processing. Each tube with a swab was logged by reading the barcode with a barcode scanner, and the swab was removed from the tube and placed onto a Thermo Fisher Scientific (Waltham, MA) 2-ml deep-well 96-well plate set on top of dry ice coolant. The top part of each swab’s stick was snapped off and discarded. Immediately after all of the wells were filled with swabs, 200 µl of HPLC-grade 90% (vol/vol) ethanol-water solvent was added to each well using a multichannel pipette. Four blanks of unused swabs and extraction solvent were included onto each plate. Each plate was then sealed with a 96-well plate lid, sonicated for 10 min, and placed into the refrigerator at 2°C to extract samples overnight. After extraction, the swabs were removed from wells and discarded, the plates were placed into a lyophilizer, and the entire sample was dried down and then resuspended in 200 µl 90% (vol/vol) ethanol-water. The plates were resealed and centrifuged at 2,000 rpm for 10 min. The 100-µl aliquots of sample were then transferred onto a Falcon 96-well MS plate using a multichannel pipette, and each plate was immediately sealed with sealing film. The MS plates were centrifuged at 2,000 rpm for 10 min and stored at 2°C until analysis.

### HPLC-MS analysis.

The metabolomics analysis of samples was conducted using reverse-phase (RP) high-performance liquid chromatography mass spectrometry (HPLC-MS). The HPLC-MS analysis was performed on a Dionex UltiMate 3000 Thermo Fisher Scientific high-performance liquid chromatography system (Thermo Fisher Scientific, Waltham, MA) coupled to a Bruker Impact HD quadrupole time of flight (qTOF) mass spectrometer. The chromatographic separation was carried out on a Kinetex C_18_ 1.7-µm, 100-Å ultrahigh-performance liquid chromatography (UHPLC) column (50 mm by 2.1 mm) (Phenomenex, Torrance, CA), held at 40°C during analysis. A total of 5 µl of each sample was injected. Mobile phase A was water, and mobile phase B was acetonitrile, both with added 0.1% (vol/vol) formic acid. The solvent gradient table was set as follows: initial mobile phase composition was 5% B for 1 min, increased to 40% B over 1 min and then to 100% B over 6 min, held at 100% B for 1 min, and decreased back to 5% B in 0.1 min, followed by a washout cycle and equilibration for a total analysis time of 13 min. The scanned *m/z* range was 80 to 2,000, the capillary voltage was 4,500 V, the nebulizer gas pressure was 2 × 10^5^ Pa, the drying gas flow rate was 9 liters/min, and the temperature was 200°C. Each full MS scan was followed by tandem MS (MS/MS) using collision-induced dissociation (CID) fragmentation of the seven most abundant ions in the spectrum. For MS/MS, the collision cell collision energy was set at 3 eV and the collision energy was stepped 50%, 75%, 150%, and 200% to obtain optimal fragmentation for differentially sized ions. The scan rate was 3 Hz. An HP-921 lock mass compound was infused during the analysis to carry out postprocessing mass correction.

### MS data analysis.

The collected HPLC-MS raw data files were first converted from Bruker’s *d* to mzXML format and then processed with the open-source OpenMS 2.0 software (78) in order to deconvolve and align each peak across different chromatograms (feature detection). The alignment window was set at 0.5 min, the noise threshold was set at 1,000 counts, the chromatographic peak full width at half-maximum (FWHM) value was set at 20, and the mass error was set at 30 ppm. All of the peaks that were present in any of the blanks with a signal-to-noise ratio (S/N) below 10:1 were removed from the final feature table. The number of features with corresponding MS/MS was as follows: VioScreen study sample cohort, 5,144 total MS2 features; antibiotic study sample cohort, 8,288 total MS2 features. The number of MS1 features is difficult to estimate exactly as it depends on feature detection settings and the number of samples, but it is typically about 4- to 5-fold greater depending on the sample. For all of the MS1 features detected across all samples, only ~1 to 5% are (35) present in an individual sample.

### Molecular networking.

Raw mass spectrometry data files were converted to the .mzXML format using Bruker Data Analysis software and uploaded to the GNPS (https://gnps.ucsd.edu/) MassIVE mass spectrometry database (https://massive.ucsd.edu/). Molecular networking (35) was performed to identify spectra shared between different sample types and to identify known molecules in the data set. All annotations are at level 2/3 according to the proposed minimum standards in metabolomics (36). The data were filtered by removing all MS/MS peaks within ±17 Da of the precursor *m/z*. MS/MS spectra were window filtered by choosing only the top 6 peaks in the ±50-Da window throughout the spectrum. The MS spectra were then clustered with the MS-Cluster algorithm with a parent mass tolerance of 0.02 Da and an MS/MS fragment ion tolerance of 0.02 Da to create consensus spectra (34, 75). Further, consensus spectra that contained fewer than 4 spectra were discarded. A network was then created where edges were filtered to have a cosine score above 0.65 and more than 5 matched peaks. The edges between two nodes were kept in the network if and only if each of the nodes appeared in each other’s respective top 10 most similar nodes. The spectra in the network were then searched against GNPS spectral libraries (34). The library spectra were filtered in the same manner as the input data. All library matches were required to have a score above 0.7 and at least 6 matched peaks. Molecular networks were visualized and mined using Cytoscape software (79).

Chemical annotations were carried out by automatic matching of fragmentation spectra to multiple databases using Global Natural Product Social Molecular Networking (GNPS) (34) and then examining the data at the MS/MS level by molecular networking (35). The goal is to retrieve spectra with identical and similar fragmentation patterns and combine them into consensus nodes and clusters, respectively. The consensus node spectra are then compared against public MS/MS libraries to provide molecular annotations (80). Further annotations could be suggested by examining the molecular network (35) (enabling the propagation of annotations). The links to the job for the molecular networks used for this study can be found at http://gnps.ucsd.edu/ProteoSAFe/status.jsp?task=9bd16822c8d448f59a03e6cc8f017f43 (antibiotic subset) and http://gnps.ucsd.edu/ProteoSAFe/status.jsp?task=d26ae082b1154f73ac050796fcaa6bda (type of plant subset).

### Molecular networking-based propagation of annotations.

Library matching was done with the GNPS reference library; however, many nodes that have no annotations are matched to a neighboring node that did have a match. Consequently, manual annotation of compounds was carried out in two steps. The exact mass of compounds and their MS/MS fragmentation spectra were matched to the reference spectra found in supplementary information in reference 39 (Fig. S4A). In addition, commendamide and its analog were identified by matching the exact mass of the corresponding ion and by *in silico* prediction of the MS/MS fragmentation spectra with CSI:FingerID (81) (Fig. S4B). For novel molecules that were found within the cluster of compounds of interest but were not described in the literature previously, the structure was postulated using annotation propagation from adjacent annotated nodes in the cluster as described in reference 34 by assessing differences in parent mass and fragmentation patterns. Annotations obtained with precursor and MS/MS matching are considered level 2 or 3 annotations according to the 2007 metabolomics standards initiative (36). All molecular networking analyses and annotations are available here: antibiotic use subset (82), type of plant subset (83), cell cultures of isolates (84), and fecal samples conetworked with the cell cultures (85). The raw data contain a significant number of abundant features originating from swab polymers. Therefore, selective background peak removal was carried out specifically for the polymer compounds originating from swabs that were used for the sample collection. The *m/z* shifts that correspond to the polymer repeating units (44.0262, 88.0524, 132.0786, and 176.1049) were identified with the GNPS *m/z* difference frequency plot. The network clusters that contained nodes with the corresponding mass differences were deemed to belong to polymers, and all member nodes of the network clusters were removed from the feature table (a total of 1,632 features/nodes). Principal-coordinate analysis (PCoA) using a Hellinger distance (86) matrix was used to confirm that the batch effect corresponding to the batches of swabs was mitigated prior to further analysis. To confirm putative annotations, authentic standards were purchased for linoleic acid (LA; Spectrum Laboratory Products, Inc., USA), conjugated linoleic acid (CLA; mixture of 4 isomers: 9,11 and 10,12 isomers, E and Z) (Sigma-Aldrich, USA), and selected antibiotics—tetracycline, oxytetracycline, and doxycycline (Abcam Inc., USA). For level 1 identifications, each authentic compound was analyzed under identical experimental conditions and retention times (RTs) and MS/MS spectra were compared with putatively annotated compounds.

### Molecular networking-based propagation of annotations for the N-acyl ligands.

The annotation of GPCR agonist compounds was not possible via direct library matching, as their spectra are not present in any MS libraries, but direct comparison with fragmentation patterns presented in reference 36 allowed us to establish these compounds’ identity with level 3 identification. Consequently, manual annotation of compounds was carried out in two steps. The exact mass of compounds and their MS/MS fragmentation spectra were matched to the reference spectra found in the supplemental material (Fig. S4A). Compound *m/z* 611.5357 was identified in this fashion. In addition, commendamide (330.2640) and its analog (*m/z* 344.2799) were identified by matching the exact mass of the corresponding ion and by *in silico* prediction of the MS/MS fragmentation spectra with CSI:FingerID (81) (Fig. S4B). For novel molecules that were found within clusters of compounds of interest but were not described in the literature previously, the structure was postulated using annotation propagation from adjacent annotated nodes in the cluster as described in reference 34 by assessing differences in parent mass and fragmentation patterns. The key structure, *m/z* 387.322, has been annotated as *N*-3-OH-palmitoyl ornithine based on the exact mass and previous annotation (39) as well as analysis of fragmentation patterns to confirm structural moieties of fragments (Fig. S4C). The rest of the structural assignments have been propagated from that structure. The ornithine moiety has been determined to be present in each structure (due to the presence of the signature ion with *m/z* 115.09), and acylation of the hydroxyl is not possible due to insufficient mass of the structures; thus, the changing mass was postulated to correspond to different lengths of the alkyl substituent (Fig. 6).

### Selective feature detection.

Selective feature extraction was performed with open-source MZmineb2 software (87). To separate closely eluting LA and CLA isomers as well as separate various N-acyl amides, crop filtering with a retention time (RT) range of 5.4 to 6.0 min and an *m/z* range of 281.246 to 281.248 was applied to all chromatograms. Mass detection was performed with a signal threshold of 1.0E2 and an 0.6-s minimum peak width. The mass tolerance was set to 20 ppm, and the maximum allowed retention time deviation was set to 5 s. For chromatographic deconvolution, the baseline cutoff algorithm with a 5.0E1 signal threshold was used. The maximum peak width was set to 0.5 min. Similarly, the MS feature for the reference compound stercobilin was extracted with a crop filter RT range of 2.0 to 4.0 min and an *m/z* range of 595.345 to 595.355. The stercobilin reference compound was used to assess the variability of chromatographic retention times to ensure that the retention times of compounds of interest (LA and CLA in particular) were correctly identified. After isotope peak removal, the peak lists of all samples were aligned within the corresponding retention time and mass tolerances. Gap filling was performed on the aligned peak list using the peak finder module with 1% intensity, 10-ppm *m/z* tolerance, and 0.05-min RT tolerance, respectively. After the creation and export of a feature matrix containing the feature retention times, exact mass, and peak areas of the corresponding extracted ion chromatograms, we added sample metadata to the feature matrix metadata of the samples.

The selective feature extraction with the same settings has been performed for all of the detected compounds listed in Fig. S4A to I (the *m/z* range crop filter window was set for corresponding *m/z* for each compound).

### Correlations of metabolites with metadata.

We have investigated correlations between metabolites (especially those of interest, such as N-acyl amides) and all of the categories in the metadata. The data were subsetted into the VioScreen and antibiotic cohorts and normalized using probabilistic quotient normalization (88). In order to test the association of the metabolites with the categorical metadata fields, we performed the Kruskal-Wallis test followed by Benjamini and Hochberg FDR correction for all metabolites. The significant metabolite-metadata associations (adjusted *P* value < 0.05) were further connected to GNPS spectral library matches associating the MS1 feature with the MS2 precursor ion in a 10-ppm mass window and 20-s retention time window. The results are summarized in Table S5.

### Data pretreatment for statistical analysis.

A PCoA plot using Hellinger distance (distance matrix, Hellinger; grouping, hierarchical cluster analysis) was built with all samples in the subset; one sample was found to be an outlier and removed. The data were then filtered to remove features with near-constant, very small values and values with low repeatability using the interquartile range (IQR) estimate. A detailed description of the methodology is given in reference 89. The samples were normalized by sum total of peak intensities, an important step due to the large variability of the fecal material load on different swabs. To reduce the effect of background signal and make the sum normalization appropriate, the subtraction of blank and polymer peak features was conducted prior to analysis, as described above. The data were further scaled by mean centering and dividing by standard deviation for each feature.

The data were split into two groups for downstream analysis. Group 1 contained samples from individuals answering “more than 30” (*n* = 41) and “less than 10” (*n* = 44) to the main American Gut Project survey question “In an average week, how many different plants do you eat?” Group 2 contained samples from individuals answering “antibiotic use within last week” (*n* = 56) and “I have not taken antibiotics in the past year” (*n* = 115) to the main American Gut Project survey question “I have taken antibiotics in the last ____.” for the antibiotic history study, correspondingly.

The resultant feature tables were used as input for the MetaboAnalyst software (90). Partial least-squares discriminant analysis (PLS-DA) (65) was used to explore and visualize variance within data and differences among experimental categories. Random forests (91) (RF) supervised analysis was used to further verify the validity of determined discriminating features.

### Expanded bloom assessment.

The American Gut Project data set now spans multiple omics types and includes data that were unavailable during the analysis described in the work of Amir et al. (15). To better understand how the blooming organisms impacted the samples in the American Gut, we (i) performed an additional set of 16S-based experiments; (ii) cultured historical samples covering a range of bloom fractions, characterized their metabolites, and sequenced the isolates; (iii) performed shotgun metagenomics sequencing on the “high-bloom” samples; (iv) ran the set of samples previously run for HPLC-MS (e.g., the plant and antibiotic cohorts) for shotgun metagenomics; and (v) ran the storage samples from reference 17 for shotgun metagenomics. The additional sequencing effort was to provide a basis to assess whether functional potential driven by the blooms was impacting any of the biological results discussed in this paper. The additional HPLC-MS work was to characterize the metabolites specific to the blooms to remove them from analysis.

### 16S-based bloom experiments.

Effect size calculations were computed prior to and following the removal of bloom reads using the procedure described by Amir et al. (15). The fraction of reads recruiting to blooms was included as a covariate. Effect sizes were assessed over Faith’s phylogenetic diversity (9), unweighted UniFrac (13), and weighted UniFrac (92). We then computed Pearson and Spearman correlations of the effect sizes, per metric, between the bloom and bloom-removed result (Fig. 2C and D). In addition to the effect size calculations, we also tested whether the bloom fraction was correlated with any metadata category and did not observe significant correlations.

We then tested the removal of blooms from other studies in which room-temperature shipping was not performed by retrieving a wide variety of human fecal studies from Qiita. UniFrac distance matrices were computed prior to and following bloom removal, followed by Mantel tests. The results of this procedure are outlined in Table S4.

10.1128/mSystems.00031-18.9 TABLE S4  Application of the filter for blooms to other human fecal studies which were not subjected to room-temperature shipping, taxonomy of the draft isolate genomes, the specific bloom 16S sOTUs observed, and ubiquitous colibactin-like biosynthetic gene clusters (top) and a unique surfactin-like biosynthetic gene cluster observed in the bloom isolates. Download TABLE S4, XLSX file, 0.01 MB. Copyright © 2018 McDonald et al. 2018 McDonald et al. This content is distributed under the terms of the Creative Commons Attribution 4.0 International license.

Finally, we correlated the relative intensities of the HPLC-MS data associated with the antibiotic and plant cohorts against the fraction of blooming reads. Critically, we observed a set of spectra that are significantly correlated (Table S5) with this fraction. On annotation using molecular networking (discussed in detail under “HPLC-MS analysis” above), we observed these metabolites to putatively be lysophosphatidylethanolamine (LysoPE), a lysophospholipid (LPL) which has previously been associated with the release of colicin (93). These metabolites were removed from subsequent analyses.

10.1128/mSystems.00031-18.10 TABLE S5  A set of molecular features which appeared to significantly correlate with the bloom fraction and Kruskal-Wallis tests for metabolites in the antibiotic and VioScreen cohorts of samples. Download TABLE S5, XLSX file, 0.1 MB. Copyright © 2018 McDonald et al. 2018 McDonald et al. This content is distributed under the terms of the Creative Commons Attribution 4.0 International license.

### Culturing.

Primary specimens (*n* = 214) were selected from three plates based on the median fraction of reads recruiting to the blooms across the plate, whether the primary specimen still existed, and the aim of gathering samples from at least the United States (*n* = 116) and United Kingdom (*n* = 73); additional countries were included in smaller sample sizes and include Australia (*n* = 7), Germany (*n* = 7), Canada (*n* = 3), Croatia (*n* = 2), Belgium (*n* = 2), France (*n* = 1), Austria (*n* = 1), Sweden (*n* = 1), and the Czech Republic (*n* = 1). The bloom typically observed in these samples (and in the full AGP data set) is E. coli (ID 04195686f2b70585790ec75320de0d6f from reference 15), although a few of the other bloom sequences were represented at high read fraction as well. Samples were retrieved from −80°C and thawed on ice. The swab head was broken off into 500 µl sterile 1× Dulbecco’s phosphate-buffered saline and vortexed vigorously for 30 s. Serial dilutions from this initial stock were made, including 1:10,000 and 1:1,000,000. Ten microliters of the 1:10,000 dilution was inoculated into 1.5 ml sterile tryptic soy broth (TSB; BD catalog no. 2253534) in sterile 96-deep-well plates (community cultures [CC]) and incubated overnight at 37°C on an orbital shaker at 500 rpm. Values of optical density at 600 nm (OD_600_) above 0.1 (TSB controls measured ~0.08) were counted as positive growth. Samples with a high bloom fraction tended to grow overnight under ambient conditions, and samples with a low bloom fraction tended to not grow under these conditions (Fig. 2A). Additionally, 100 µl of each dilution was plated onto tryptic soy agar using sterile glass beads and incubated overnight at 37°C. The following morning, a picture of the best dilution was captured and the most representative colony was selected from each plate and inoculated into 1.5 ml sterile TSB for overnight incubation as described above (isolates [IS]). The following morning, OD_600_ measurements were taken and the cultures were pelleted at 3,000 × *g* for 5 min. The supernatant and cell pellets were stored at −20°C for metabolomic analysis and DNA extraction, respectively.

### Shotgun sequencing of cultured specimens.

Shotgun sequencing was performed on all isolates and community cultures using a 1:10 miniaturized Nextera library preparation with 1 ng genomic DNA (gDNA) input or up to 1 µl and a 15-cycle PCR amplification. Libraries were quantified with the PicoGreen double-stranded DNA (dsDNA) assay kit, and 50 ng of each library (or a 4-µl maximum) was pooled. The library was size selected for 200 to 700 bp using the Sage Bioscience Pippin Prep and sequenced as a paired-end 150-cycle run on an Illumina HiSeq 2500 v2 sequencer in Rapid Run mode at the UCSD IGM Genomics Center. Sequence processing, including assembly performed as described for metagenomic processing below, with the exception that “--meta” was not used with SPAdes (94), and read binning against the resulting contigs was not performed. For each isolate, contigs with abnormally high or low coverage as defined by the 1.5× interquartile range (IQR) rule were dropped. The characterization of the metabolites from the supernatant using HPLC-MS is discussed under “HPLC-MS analysis” above.

Following assembly of the draft genomes, taxonomic assessment by Kraken (95) revealed that of the 119 successfully sequenced colony isolate cultures, 95 matched the bloom organisms identified by Amir et al. (15). Compellingly, 70 of these isolate genomes contained exact 16S sequence matches to a bloom organism identified by reference 15, including 65 which matched the dominant E. coli bloom in the American Gut (Table S4).

### Biosynthetic gene cluster analysis of isolates.

The read data for the isolates were then assessed for predicted biosynthetic gene clusters (BGCs). We used biosyntheticSPAdes (D. Meleshko, H. Mohimani, I. Hajirasouliha, M. H. Medema, A. Korobeynikov, and P. Pevzner, submitted for publication) to analyze BGCs in the assembly graph of individual genomes. Below, we focus on the longest BGCs that are particularly difficult to reconstruct based on *ad hoc* analysis of contigs and reveal their variations (which likely translate into variations of their natural products). Some of the reconstructed long BGCs are ubiquitous (shared by many isolates, albeit with some variations), while others are unique, e.g., present in a single or a small number of isolates. We identified BGCs, representing in the alphabet of their domains (Table S4), and uncovered variations in their sequence across multiple isolates. Specifically, we found a ubiquitous BGC similar to the elusive peptide-polyketide genotoxin colibactin and a unique surfactin-like BGC. Colibactin triggers DNA double-strand breaks in eukaryotic cells (96, 97) and induces cellular senescence and metabolic reprogramming in affected mammalian cells (98). Of the 11 samples containing the longest colibactin-like BGC, 10 of them contained the exact E. coli bloom 16S sequence described above; the 11th isolate was actually a canine fecal sample plated alongside human samples (as the AGP allows participants to submit pet samples).

Although colibactin is frequently harbored by various E. coli strains, the variations of colibactin BGCs across various isolates have not been studied before. Genomic analysis revealed wide variations in colibactin-like BGCs, suggesting that various strains produce related but not identical variants of natural products (99). These variations may give rise to the suite of LysoPE-associated spectra identified between the 16S and HPLC-MS data sets.

### Shotgun sequencing of the high-bloom and storage samples.

Previously extracted DNA from the high-bloom samples used for culturing was obtained, as was previously extracted DNA from the work of Song et al. (17). Shotgun sequencing libraries from a total of 5 ng (or 3.5 µl maximum) gDNA were used in a 1:10 miniaturized Kapa HyperPlus protocol with a 15-cycle PCR amplification. Libraries were quantified with the PicoGreen dsDNA assay kit, and 50 ng (or 1 µl maximum) of each library was pooled. The pool was size selected for 300 to 700 bp and sequenced as a paired-end 150-cycle run on an Illumina HiSeq 2500 v2 sequencer in Rapid Run mode at the UCSD IGM Genomics Center. Sequence processing, including assembly, was performed as described for metagenomic processing below.

### Functional assessment of conjugated and nonconjugated linoleic acid.

To investigate the metabolic potential of the gut microbiome for producing conjugated linoleic acid from linoleic acid, we estimated the abundance of linoleic acid isomerase (LAI) in the fecal metagenome. We focused this investigation on the “plant” cohort, which were samples selected to maximize the difference between the number of types of plant metadata category as discussed above. First, we translated the assembled metagenomes to metaproteomes using Prodigal gene prediction software. To map LAI to these metaproteomes, we used a representative LAI protein sequence (UniProt D2BQ64), which was matched against UniProtKB (via https://www.ebi.ac.uk/Tools/hmmer/) for multiple sequence alignment (MSA). The resulting MSA file in Clustal format was then used to generate a hidden Markov model (HMM) profile for LAI using hmmbuild in HMMER software (100). Subsequently, we mapped the resulting HMM profile to sample metaproteomes using hmmsearch with an E value threshold of 10E−5. We calculated abundances of LAI per sample based on abundance (coverage × length) of LAI containing contigs in each sample, normalized to total sample biomass, and performed linear regression between LAI abundances and bloom fraction. We did not note any correlation between metabolic potential of gut metagenome to produce LAI and the fraction of blooming bacteria (samples with no LAI hits were removed from this analysis). Similarly, there was no correlation between CLA abundances and bloom fraction in the samples. These results suggest that our report on the differential abundance of CLA in subjects with different dietary practices (with respect to the number of different types of plants consumed) is unlikely to be confounded by the presence of blooming bacteria.

### Storage sample assessment.

Metagenomic reads from the storage samples were mapped to the 169 isolate assemblies. We then ran model comparison tests on each to determine which mappings were significantly different between frozen samples and samples left out at ambient temperatures for various periods of time. Using the ‘lme’ package (101) in R (v3.3.3, R Core Team, 2017), linear mixed-effects models were applied to the abundances, with individual treated as the random effect. Mappings were considered to be significantly associated with temperature if the model was significantly improved (ANOVA *P* ≤ 0.05) by incorporating a fixed effect of temperature. Seven mappings to isolates were found to be significantly increased in samples stored at ambient temperatures compared to frozen samples in both storage studies, of which 3 contained the 16S of the dominant E. coli bloom in the AGP samples and 2 contained the 16S from other blooms recognized by the work in reference 15.

### Shotgun sequence processing.

Raw Fastq files were processed using Atropos v1.1.5 (102) to remove adapters and low-quality regions. Putative human genome contaminations were identified and removed by using Bowtie 2 v2.3.0 (103) with the “--very-sensitive” option against the human reference genome GRCh37/hg19.

Sequences were assigned taxonomy using Kraken v1.0.0 (95) against the “standard” database built according to the Kraken manual, which contains all complete bacterial, archeal, and viral genomes available from NCBI RefSeq as of 3 August 2017. Results were processed using Bracken v1.0.0 (104) to estimate the relative abundance of species-level taxa.

Metagenome sequencing data were assembled using SPAdes v3.11.1 (94) with the “--meta” flag enabled (105). Contigs of ≥1 kb in length were retained and fed to the prokaryotic genome annotation pipeline Prokka v1.12 (106). Putatively individual genomes were inferred using MaxBin2 v2.2.4 (107).

In parallel, contigs were sheared into 200-bp fragments and taxonomy was assigned using Kraken (see above). For each contig, the most assigned taxon at each taxonomic rank and the proportion of sequences assigned to it were inferred.

A total of 3,725 genome bins were identified from 677 out of 780 AGP metagenomes, with 5.50 ± 4.05 bins per sample and a maximum bin number of 30. Bins with completeness of <50% were dropped, leaving 1,029 bins from 464 samples (2.22 ± 1.97 bins per sample, maximum bins = 19).

### Antimicrobial resistance gene assessment.

There are 780 metagenome shotgun samples in total, of which 713 have at least 100,000 reads and have corresponding metadata. Of these, 240 belong to the antibiotic (“Abx”) cohort, and 203 belong to the “plant” cohort.

We ran FragGeneScan (v1.30 “run_FragGeneScan.pl -complete=0 -train=illumina_10 -genome=<input.fa> -out=<out.file>”) (108) on quality-controlled reads to infer partial protein sequences and then ran HMMER (v3.1b2 “hmmsearch --cut_tc --cpu 4 --tblout <out.file> <resfam.db> <input.file>”) (109) against the ResFam database, version 1.2.2 (110, 111), to identify putative antibiotic resistance genes.

The ResFam database contains entries for 170 antimicrobial resistance (AMR) gene families. Of these, 122 belong to at least 1 of 16 antibiotic classes, all 170 belong to at least 1 of 21 mechanism classes, and 42 belong to 5 beta-lactamase classes in the Ambler classification.

We normalized the counts against the total number of reads per sample. The results were converted to BIOM tables, filtered to retain valid samples only (see above), and collapsed from family to the three types of classes (see above). The relative abundance table containing antibiotic classes was used for downstream analysis.

For each metadata category, the upper and lower extrema were selected for comparison. The Kruskal-Wallis *H* test (using the SciPy command Kruskal) was performed to assess the differential abundance between upper and lower extrema for each of the nine antibiotic classes. The *H* statistic and *P* value were reported for each antibiotic class.

Specifically, for the cohort “Abx,” the metadata column “antibiotic_history” was tested. Categories “Week” and “Month” were merged into “within 1 month”; “I have not taken antibiotics in the past year.” was abbreviated as “beyond 1 year.” Other categories were omitted.

For the cohort “plant,” metadata column “types_of_plants” was tested. Categories “less than 5” and “6 to 10” were merged into “10 or less”; “More than 30” was retained; others were omitted.

### Filtering bacterial blooms for metabolomics analysis.

To assess and account for the impact of the metabolites contributed by these organisms, we have performed HPLC-MS analysis of cultures of blooming organisms to establish possible contributions, as described above. It was found that there is a negligible overlap of the bloom-associated metabolites with the compounds detected in AGP samples (Fig. 2B). Furthermore, we have verified that none of the compounds discussed in this work (LA, CLA, and compounds in Fig. S4A to I) are present in these bloom cultures. The main organism implicated in bloom was determined to be E. coli, as described earlier, and MS data corroborate these findings (network not shown).

Considering that the metabolites resulting from microbial activity in cultures can differ significantly from those *in vivo* (e.g., many of the metabolites could originate not from *de novo* synthesis but rather from microbial modifications of external compounds that are not present in medium, e.g., from the host), we also explored associations of metabolites in AGP metabolomics samples and blooms. Spearman rank correlation analysis of the fraction of 16S reads in a sample reporting as bloom to metabolites observed in the same samples revealed several features that correlate significantly (Table S5). There exists a significant overlap between the antibiotic and VioScreen study subsets, indicating a potential common origin of these features. The strongest correlation was found for the feature *m/z* 480.3106 with multiple bloom organisms (ρ^2 > 0.25 for E. coli at *P* < 1e−40). This feature was found to also significantly correlate with the principal coordinates of the PCoA, with and without blooms in the UniFrac matrices for both subsets. The tentative annotation of this feature is LysoPE, a lysophospholipid (LPL). The production of LPLs *in vivo* is a result of phospholipase A enzymatic activity associated with Gram-negative bacteria. It is known that LysoPE is essential for release of colicin (93). Colicin (by itself not detectable with the MS methodology in this study due to very high molecular mass) is a bacteriocin related to microbial warfare and is known to be produced by E. coli, the major bloomer in AGP. It can be suggested that the blooming of an organism is related to attempting to kill competitors to maximize nutrient availability. Importantly, removal of all of the features associated with bloom does not alter the metabolomics results at all, which indicates that all of the observed biological trends reported here are not related to blooms.

### Mental health in the American Gut Project.

From the AGP cohort, we selected subjects who endorsed a mental health disorder (depression, schizophrenia, PTSD, and/or bipolar disorder). This resulted in 1,140 subjects. Six hundred thirty-six subjects endorsed at least one of the exclusion criteria (antibiotic use in the last year, IBD, Clostridium difficile infection, pregnancy, Alzheimer’s disease, anorexia or bulimia, history of substance use disorder, epilepsy or seizure disorder, kidney disease, and phenylketonuria). Out of the remaining 504 subjects, 319 did not provide information regarding country of residence, hence forming a case cohort of 185 subjects. The remaining samples were further filtered down to 125 samples to include only high-quality fecal microbiome data (at least 1,250 sequences/sample) at a single time point per subject. For those cases, we created a 1:1 matched sample of patients and nonpsychiatric comparison (NC) participants based on age (±5 years), BMI, history of diabetes, smoking frequency, country of residence, census region (if in the United States), and sequencing plate. For each of the cohorts, we calculated beta-diversity distance matrices using Bray-Curtis dissimilarity and weighted UniFrac. On the resulting matrices, we ran pairwise PERMANOVA with 999 permutations between “cases” (people who reported mental illness) and NCs (outmatched control data set). Differential abundance testing was performed using the permuted mean difference test at 10,000 permutations, with discrete FDR (112) correction at alpha = 0.1.

### Data availability.

All sequence data and deidentified participant responses can be found in EBI under project PRJEB11419 and Qiita study ID 10317. Per-sample EBI accession numbers and download links partitioned by the sequencing run are described in Table S1. The sOTU table following bloom removal containing the 9,511 samples used in the manuscript can be accessed from figshare in both read count (113) and relative abundance (114), as well as the corresponding phylogeny (115), sample metadata (116), collated alpha-diversities (117), and unweighted (118) and weighted (119) UniFrac distance matrices. For the mass spectrometry, data are accessed through https://gnps.ucsd.edu: MSV000080187 (antibiotic study cohort), MSV000080186 (plant consumption frequency study cohort), and MSV000081777 (cell cultures of blooms). For the novel annotated structures of N-acyl amides, the reference MS/MS spectra at GNPS are CCMSLIB00004679232, CCMSLIB00004679233, CCMSLIB00004679234, CCMSLIB00004679235, CCMSLIB00004679236, CCMSLIB00004679237, CCMSLIB00004679238, CCMSLIB00004679239, and CCMSLIB00004679240. All of the raw data are publicly available at the UCSD Center for Computational Mass Spectrometry (36) (data set ID MassIVE MSV000080179). The additional sequence data generated from the American Gut samples were deposited in EBI under the American Gut accession (ERP012803), and the storage sample data were deposited under their accession (ERP015155). The deidentified participant and sequence data for the American Gut are available from the European Bioinformatics Institute under accession number ERP012803. Per-sample accession numbers are included in the supplemental material (Table S1). The EBI accession includes more samples than presented here, as consent for some samples was obtained after the analysis was completed.

## References

[1] Thompson LR, Sanders JG, McDonald D, Amir A, Ladau J, Locey KJ, Prill RJ, Tripathi A, Gibbons SM, Ackermann G, Navas-Molina JA, Janssen S, Kopylova E, Vázquez-Baeza Y, González A, Morton JT, Mirarab S, Zech Xu Z, Jiang L, Haroon MF, Kanbar J, Zhu Q, Jin Song S, Kosciolek T, Bokulich NA, Lefler J, Brislawn CJ, Humphrey G, Owens SM, Hampton-Marcell J, Berg-Lyons D, McKenzie V, Fierer N, Fuhrman JA, Clauset A, Stevens RL, Shade A, Pollard KS, Goodwin KD, Jansson JK, Gilbert JA, Knight R, Earth Microbiome Project 2017 A communal catalogue reveals Earth’s multiscale microbial diversity. Nature 551:457–463. doi:10.1038/nature24621. 29088705 PMC6192678

[2] Falony G, Joossens M, Vieira-Silva S, Wang J, Darzi Y, Faust K, Kurilshikov A, Bonder MJ, Valles-Colomer M, Vandeputte D, Tito RY, Chaffron S, Rymenans L, Verspecht C, De Sutter L, Lima-Mendez G, D’hoe K, Jonckheere K, Homola D, Garcia R, Tigchelaar EF, Eeckhaudt L, Fu J, Henckaerts L, Zhernakova A, Wijmenga C, Raes J 2016 Population-level analysis of gut microbiome variation. Science 352:560–564. doi:10.1126/science.aad3503. 27126039

[3] Qin J, Li R, Raes J, Arumugam M, Burgdorf KS, Manichanh C, Nielsen T, Pons N, Levenez F, Yamada T, Mende DR, Li J, Xu J, Li S, Li D, Cao J, Wang B, Liang H, Zheng H, Xie Y, Tap J, Lepage P, Bertalan M, Batto JM, Hansen T, Le Paslier D, Linneberg A, Nielsen HB, Pelletier E, Renault P, Sicheritz-Ponten T, Turner K, Zhu H, Yu C, Li S, Jian M, Zhou Y, Li Y, Zhang X, Li S, Qin N, Yang H, Wang J, Brunak S, Doré J, Guarner F, Kristiansen K, Pedersen O, Parkhill J, Weissenbach J, MetaHIT Consortium, Bork P, Ehrlich SD, Wang J, Wang J 2010 A human gut microbial gene catalogue established by metagenomic sequencing. Nature 464:59–65. doi:10.1038/nature08821. 20203603 PMC3779803

[4] Zhernakova A, Kurilshikov A, Bonder MJ, Tigchelaar EF, Schirmer M, Vatanen T, Mujagic Z, Vila AV, Falony G, Vieira-Silva S, Wang J, Imhann F, Brandsma E, Jankipersadsing SA, Joossens M, Cenit MC, Deelen P, Swertz MA, LifeLines Cohort Study, Weersma RK, Feskens EJM, Netea MG, Gevers D, Jonkers D, Franke L, Aulchenko YS, Huttenhower C, Raes J, Hofker MH, Xavier RJ, Wijmenga C, Fu J 2016 Population-based metagenomics analysis reveals markers for gut microbiome composition and diversity. Science 352:565–569. doi:10.1126/science.aad3369. 27126040 PMC5240844

[5] Human Microbiome Project Consortium 2012 Structure, function and diversity of the healthy human microbiome. Nature 486:207–214. doi:10.1038/nature11234. 22699609 PMC3564958

[6] Yatsunenko T, Rey FE, Manary MJ, Trehan I, Dominguez-Bello MG, Contreras M, Magris M, Hidalgo G, Baldassano RN, Anokhin AP, Heath AC, Warner B, Reeder J, Kuczynski J, Caporaso JG, Lozupone CA, Lauber C, Clemente JC, Knights D, Knight R, Gordon JI 2012 Human gut microbiome viewed across age and geography. Nature 486:222–227. doi:10.1038/nature11053. 22699611 PMC3376388

[7] Flores GE, Caporaso JG, Henley JB, Rideout JR, Domogala D, Chase J, Leff JW, Vázquez-Baeza Y, Gonzalez A, Knight R, Dunn RR, Fierer N 2014 Temporal variability is a personalized feature of the human microbiome. Genome Biol 15:531. doi:10.1186/s13059-014-0531-y. 25517225 PMC4252997

[8] Song SJ, Lauber C, Costello EK, Lozupone CA, Humphrey G, Berg-Lyons D, Caporaso JG, Knights D, Clemente JC, Nakielny S, Gordon JI, Fierer N, Knight R 2013 Cohabiting family members share microbiota with one another and with their dogs. Elife 2:e00458. doi:10.7554/eLife.00458. 23599893 PMC3628085

[9] Faith DP 1992 Conservation evaluation and phylogenetic diversity. Biol Conserv 61:1–10. doi:10.1016/0006-3207(92)91201-3.

[10] Morton JT, Sanders J, Quinn RA, McDonald D, Gonzalez A, Vázquez-Baeza Y, Navas-Molina JA, Song SJ, Metcalf JL, Hyde ER, Lladser M, Dorrestein PC, Knight R 2017 Balance trees reveal microbial niche differentiation. mSystems 2:e00162-16. doi:10.1128/mSystems.00162-16. 28144630 PMC5264246

[11] Clemente JC, Pehrsson EC, Blaser MJ, Sandhu K, Gao Z, Wang B, Magris M, Hidalgo G, Contreras M, Noya-Alarcón Ó, Lander O, McDonald J, Cox M, Walter J, Oh PL, Ruiz JF, Rodriguez S, Shen N, Song SJ, Metcalf J, Knight R, Dantas G, Dominguez-Bello MG 2015 The microbiome of uncontacted Amerindians. Sci Adv 1:e1500183. doi:10.1126/sciadv.1500183. 26229982 PMC4517851

[12] Obregon-Tito AJ, Tito RY, Metcalf J, Sankaranarayanan K, Clemente JC, Ursell LK, Zech Xu Z, Van Treuren W, Knight R, Gaffney PM, Spicer P, Lawson P, Marin-Reyes L, Trujillo-Villarroel O, Foster M, Guija-Poma E, Troncoso-Corzo L, Warinner C, Ozga AT, Lewis CM 2015 Subsistence strategies in traditional societies distinguish gut microbiomes. Nat Commun 6:6505. doi:10.1038/ncomms7505. 25807110 PMC4386023

[13] Lozupone C, Knight R 2005 UniFrac: a new phylogenetic method for comparing microbial communities. Appl Environ Microbiol 71:8228–8235. doi:10.1128/AEM.71.12.8228-8235.2005. 16332807 PMC1317376

[14] Lozupone C, Lladser ME, Knights D, Stombaugh J, Knight R 2011 UniFrac: an effective distance metric for microbial community comparison. ISME J 5:169–172. doi:10.1038/ismej.2010.133. 20827291 PMC3105689

[15] Amir A, McDonald D, Navas-Molina JA, Debelius J, Morton JT, Hyde E, Robbins-Pianka A, Knight R 2017 Correcting for microbial blooms in fecal samples during room-temperature shipping. mSystems 2:e00199-16. doi:10.1128/mSystems.00199-16. 28289733 PMC5340865

[16] Amir A, McDonald D, Navas-Molina JA, Kopylova E, Morton JT, Zech Xu Z, Kightley EP, Thompson LR, Hyde ER, Gonzalez A, Knight R 2017 Deblur rapidly resolves single-nucleotide community sequence patterns. mSystems 2:e00191-16. doi:10.1128/mSystems.00191-16. 28289731 PMC5340863

[17] Song SJ, Amir A, Metcalf JL, Amato KR, Xu ZZ, Humphrey G, Knight R 2016 Preservation methods differ in fecal microbiome stability, affecting suitability for field studies. mSystems 1:e00021-16. doi:10.1128/mSystems.00021-16. PMC5069758 27822526

[18] Ley RE, Lozupone CA, Hamady M, Knight R, Gordon JI 2008 Worlds within worlds: evolution of the vertebrate gut microbiota. Nat Rev Microbiol 6:776–788. doi:10.1038/nrmicro1978. 18794915 PMC2664199

[19] McDonald D, Price MN, Goodrich J, Nawrocki EP, DeSantis TZ, Probst A, Andersen GL, Knight R, Hugenholtz P 2012 An improved Greengenes taxonomy with explicit ranks for ecological and evolutionary analyses of bacteria and archaea. ISME J 6:610–618. doi:10.1038/ismej.2011.139. 22134646 PMC3280142

[20] Mirarab S, Nguyen N, Warnow T 2012 SEPP: SATé-enabled phylogenetic placement. Pac Symp Biocomput 2012:247–258. 10.1142/9789814366496_0024 22174280

[21] Halfvarson J, Brislawn CJ, Lamendella R, Vázquez-Baeza Y, Walters WA, Bramer LM, D’Amato M, Bonfiglio F, McDonald D, Gonzalez A, McClure EE, Dunklebarger MF, Knight R, Jansson JK 2017 Dynamics of the human gut microbiome in inflammatory bowel disease. Nat Microbiol 2:17004. doi:10.1038/nmicrobiol.2017.4. 28191884 PMC5319707

[22] Smits SA, Leach J, Sonnenburg ED, Gonzalez CG, Lichtman JS, Reid G, Knight R, Manjurano A, Changalucha J, Elias JE, Dominguez-Bello MG, Sonnenburg JL 2017 Seasonal cycling in the gut microbiome of the Hadza hunter-gatherers of Tanzania. Science 357:802–806. doi:10.1126/science.aan4834. 28839072 PMC5891123

[23] Ladau J, Sharpton TJ, Finucane MM, Jospin G, Kembel SW, O’Dwyer J, Koeppel AF, Green JL, Pollard KS 2013 Global marine bacterial diversity peaks at high latitudes in winter. ISME J 7:1669–1677. doi:10.1038/ismej.2013.37. 23514781 PMC3749493

[24] McDonald D, Ackermann G, Khailova L, Baird C, Heyland D, Kozar R, Lemieux M, Derenski K, King J, Vis-Kampen C, Knight R, Wischmeyer PE 2016 Extreme dysbiosis of the microbiome in critical illness. mSphere 1:e00199-16. doi:10.1128/mSphere.00199-16. 27602409 PMC5007431

[25] David LA, Maurice CF, Carmody RN, Gootenberg DB, Button JE, Wolfe BE, Ling AV, Devlin AS, Varma Y, Fischbach MA, Biddinger SB, Dutton RJ, Turnbaugh PJ 2014 Diet rapidly and reproducibly alters the human gut microbiome. Nature 505:559–563. doi:10.1038/nature12820. 24336217 PMC3957428

[26] McDonald D, Robbins-Pianka A, Mann AE, Vrbanac A, Amir A, Frazier A, Gonzalez A, Tripathi A, Fahimipour AK, Brennen C, Martino C, Lebrilla C, Lozupone C, Lewis CM, Raison C, Zhang C, Lauber CL, Warinner C, Lowry CA, Callewaert C, Bloss C, Huttenhower C, Knights D, Willner D, Galzerani DD, Gonzalez DJ, Mills DA, Chopra D, Gevers D, Berg-Lyons D, Sears DD, Wendel D, Wolfe E, Lovelace E, Hyde ER, Pierce E, TerAvest E, Montassier E, Bolyen E, Bushman FD, Ackermann G, Wu GD, Church GM, Rahnavard G, Saxe G, Gogul G, Humphrey G, Holscher HD, Ugrina I, German JB, Caporaso JG, Gilbert J, Wozniak JM, Kerr J, Ravel J, Gaffney J, Lewis JD, Morton JT, Suchodolski JS, Jansson JK, Hampton-Marcell JT, Bobe J, Leach J, Raes J, Green JL, Metcalf JL, Chase JH, Eisen JA, Monk J, Navas-Molina JA, Clemente JC, et al. 2018 movie_s1.mp4. figshare doi:10.6084/m9.figshare.5936479.

[27] Gophna U, Konikoff T, Nielsen HB 2017 Oscillospira and related bacteria—from metagenomic species to metabolic features. Environ Microbiol 19:835–841. doi:10.1111/1462-2920.13658. 28028921

[28] Lombard V, Golaconda Ramulu H, Drula E, Coutinho PM, Henrissat B 2014 The carbohydrate-active enzymes database (CAZy) in 2013. Nucleic Acids Res 42:D490–D495. doi:10.1093/nar/gkt1178. 24270786 PMC3965031

[29] El Kaoutari A, Armougom F, Gordon JI, Raoult D, Henrissat B 2013 The abundance and variety of carbohydrate-active enzymes in the human gut microbiota. Nat Rev Microbiol 11:497–504. doi:10.1038/nrmicro3050. 23748339

[30] Cummings JH, Macfarlane GT 1991 The control and consequences of bacterial fermentation in the human colon. J Appl Bacteriol 70:443–459. doi:10.1111/j.1365-2672.1991.tb02739.x. 1938669

[31] Henderson G, Cox F, Ganesh S, Jonker A, Young W, Global Rumen Census Collaborators, Janssen PH 2015 Rumen microbial community composition varies with diet and host, but a core microbiome is found across a wide geographical range. Sci Rep 5:14567. doi:10.1038/srep14567. 26449758 PMC4598811

[32] O’Donnell MM, Harris HMB, Ross RP, O’Toole PW 2017 Core fecal microbiota of domesticated herbivorous ruminant, hindgut fermenters, and monogastric animals. Microbiologyopen 6. doi:10.1002/mbo3.509. PMC5635170 28834331

[33] Kohl KD, Brun A, Magallanes M, Brinkerhoff J, Laspiur A, Acosta JC, Bordenstein SR, Caviedes-Vidal E 2016 Physiological and microbial adjustments to diet quality permit facultative herbivory in an omnivorous lizard. J Exp Biol 219:1903–1912. doi:10.1242/jeb.138370. 27307545

[34] Wang M, Carver JJ, Phelan VV, Sanchez LM, Garg N, Peng Y, Nguyen DD, Watrous J, Kapono CA, Luzzatto-Knaan T, Porto C, Bouslimani A, Melnik AV, Meehan MJ, Liu W-T, Crüsemann M, Boudreau PD, Esquenazi E, Sandoval-Calderón M, Kersten RD, Pace LA, Quinn RA, Duncan KR, Hsu C-C, Floros DJ, Gavilan RG, Kleigrewe K, Northen T, Dutton RJ, Parrot D, Carlson EE, Aigle B, Michelsen CF, Jelsbak L, Sohlenkamp C, Pevzner P, Edlund A, McLean J, Piel J, Murphy BT, Gerwick L, Liaw C-C, Yang Y-L, Humpf H-U, Maansson M, Keyzers RA, Sims AC, Johnson AR, Sidebottom AM, Sedio BE, Klitgaard A, Larson CB, Boya P CA, Torres-Mendoza D, Gonzalez DJ, Silva DB, Marques LM, Demarque DP, Pociute E, O’Neill EC, Briand E, Helfrich EJN, Granatosky EA, Glukhov E, Ryffel F, Houson H, Mohimani H, Kharbush JJ, Zeng Y, Vorholt JA, et al. 2016 Sharing and community curation of mass spectrometry data with Global Natural Products Social Molecular Networking. Nat Biotechnol 34:828–837. doi:10.1038/nbt.3597. 27504778 PMC5321674

[35] Watrous J, Roach P, Alexandrov T, Heath BS, Yang JY, Kersten RD, van der Voort M, Pogliano K, Gross H, Raaijmakers JM, Moore BS, Laskin J, Bandeira N, Dorrestein PC 2012 Mass spectral molecular networking of living microbial colonies. Proc Natl Acad Sci U S A 109:E1743–E1752. doi:10.1073/pnas.1203689109. PMC3387089 22586093

[36] Sumner LW, Amberg A, Barrett D, Beale MH, Beger R, Daykin CA, Fan TW-M, Fiehn O, Goodacre R, Griffin JL, Hankemeier T, Hardy N, Harnly J, Higashi R, Kopka J, Lane AN, Lindon JC, Marriott P, Nicholls AW, Reily MD, Thaden JJ, Viant MR 2007 Proposed minimum reporting standards for chemical analysis. Chemical Analysis Working Group (CAWG) Metabolomics Standards Initiative (MSI). Metabolomics 3:211–221. doi:10.1007/s11306-007-0082-2. 24039616 PMC3772505

[37] Kishino S, Takeuchi M, Park SB, Hirata A, Kitamura N, Kunisawa J, Kiyono H, Iwamoto R, Isobe Y, Arita M, Arai H, Ueda K, Shima J, Takahashi S, Yokozeki K, Shimizu S, Ogawa J 2013 Polyunsaturated fatty acid saturation by gut lactic acid bacteria affecting host lipid composition. Proc Natl Acad Sci U S A 110:17808–17813. doi:10.1073/pnas.1312937110. 24127592 PMC3816446

[38] Coakley M, Ross RP, Nordgren M, Fitzgerald G, Devery R, Stanton C 2003 Conjugated linoleic acid biosynthesis by human-derived Bifidobacterium species. J Appl Microbiol 94:138–145. doi:10.1046/j.1365-2672.2003.01814.x. 12492934

[39] Cohen LJ, Esterhazy D, Kim SH, Lemetre C, Aguilar RR, Gordon EA, Pickard AJ, Cross JR, Emiliano AB, Han SM, Chu J, Vila-Farres X, Kaplitt J, Rogoz A, Calle PY, Hunter C, Bitok JK, Brady SF 2017 Commensal bacteria make GPCR ligands that mimic human signalling molecules. Nature 549:48–53. doi:10.1038/nature23874. 28854168 PMC5777231

[40] Hauser AS, Attwood MM, Rask-Andersen M, Schiöth HB, Gloriam DE 2017 Trends in GPCR drug discovery: new agents, targets and indications. Nat Rev Drug Discov 16:829–842. doi:10.1038/nrd.2017.178. 29075003 PMC6882681

[41] Clarke G, Stilling RM, Kennedy PJ, Stanton C, Cryan JF, Dinan TG 2014 Minireview: gut microbiota: the neglected endocrine organ. Mol Endocrinol 28:1221–1238. doi:10.1210/me.2014-1108. 24892638 PMC5414803

[42] Cani PD, Knauf C 2016 How gut microbes talk to organs: the role of endocrine and nervous routes. Mol Metab 5:743–752. doi:10.1016/j.molmet.2016.05.011. 27617197 PMC5004142

[43] Smith B, Chu LK, Smith TC, Amoroso PJ, Boyko EJ, Hooper TI, Gackstetter GD, Ryan MAK, Millennium Cohort Study Team 2008 Challenges of self-reported medical conditions and electronic medical records among members of a large military cohort. BMC Med Res Methodol 8:37. doi:10.1186/1471-2288-8-37. 18644098 PMC2447848

[44] Jiang H, Ling Z, Zhang Y, Mao H, Ma Z, Yin Y, Wang W, Tang W, Tan Z, Shi J, Li L, Ruan B 2015 Altered fecal microbiota composition in patients with major depressive disorder. Brain Behav Immun 48:186–194. doi:10.1016/j.bbi.2015.03.016. 25882912

[45] Lin P, Ding B, Feng C, Yin S, Zhang T, Qi X, Lv H, Guo X, Dong K, Zhu Y, Li Q 2017 Prevotella and Klebsiella proportions in fecal microbial communities are potential characteristic parameters for patients with major depressive disorder. J Affect Disord 207:300–304. doi:10.1016/j.jad.2016.09.051. 27741466

[46] Saraceno B 2002 The WHO World Health Report 2001 on mental health. Epidemiol Psichiatr Soc 11:83–87. doi:10.1017/S1121189X00005546. 12212469

[47] Zheng P, Zeng B, Zhou C, Liu M, Fang Z, Xu X, Zeng L, Chen J, Fan S, Du X, Zhang X, Yang D, Yang Y, Meng H, Li W, Melgiri ND, Licinio J, Wei H, Xie P 2016 Gut microbiome remodeling induces depressive-like behaviors through a pathway mediated by the host’s metabolism. Mol Psychiatry 21:786–796. doi:10.1038/mp.2016.44. 27067014

[48] Weingarden A, González A, Vázquez-Baeza Y, Weiss S, Humphry G, Berg-Lyons D, Knights D, Unno T, Bobr A, Kang J, Khoruts A, Knight R, Sadowsky MJ 2015 Dynamic changes in short- and long-term bacterial composition following fecal microbiota transplantation for recurrent Clostridium difficile infection. Microbiome 3:10. doi:10.1186/s40168-015-0070-0. 25825673 PMC4378022

[49] Koenig JE, Spor A, Scalfone N, Fricker AD, Stombaugh J, Knight R, Angenent LT, Ley RE 2011 Succession of microbial consortia in the developing infant gut microbiome. Proc Natl Acad Sci U S A 108(Suppl 1):4578–4585. doi:10.1073/pnas.1000081107. 20668239 PMC3063592

[50] McDonald D, Robbins-Pianka A, Mann AE, Vrbanac A, Amir A, Frazier A, Gonzalez A, Tripathi A, Fahimipour AK, Brennen C, Martino C, Lebrilla C, Lozupone C, Lewis CM, Raison C, Zhang C, Lauber CL, Warinner C, Lowry CA, Callewaert C, Bloss C, Huttenhower C, Knights D, Willner D, Galzerani DD, Gonzalez DJ, Mills DA, Chopra D, Gevers D, Berg-Lyons D, Sears DD, Wendel D, Wolfe E, Lovelace E, Hyde ER, Pierce E, TerAvest E, Montassier E, Bolyen E, Bushman FD, Ackermann G, Wu GD, Church GM, Rahnavard G, Saxe G, Gogul G, Humphrey G, Holscher HD, Ugrina I, German JB, Caporaso JG, Gilbert J, Wozniak JM, Kerr J, Ravel J, Gaffney J, Lewis JD, Morton JT, Suchodolski JS, Jansson JK, Hampton-Marcell JT, Bobe J, Leach J, Raes J, Green JL, Metcalf JL, Chase JH, Eisen JA, Monk J, Navas-Molina JA, Clemente JC, et al. 2018 movie_s2.mp4. figshare doi:10.6084/m9.figshare.5936482.

[51] Caporaso JG, Lauber CL, Walters WA, Berg-Lyons D, Huntley J, Fierer N, Owens SM, Betley J, Fraser L, Bauer M, Gormley N, Gilbert JA, Smith G, Knight R 2012 Ultra-high-throughput microbial community analysis on the Illumina HiSeq and MiSeq platforms. ISME J 6:1621–1624. doi:10.1038/ismej.2012.8. 22402401 PMC3400413

[52] Apprill A, McNally S, Parsons R, Weber L 2015 Minor revision to V4 region SSU rRNA 806R gene primer greatly increases detection of SAR11 bacterioplankton. Aquat Microb Ecol 75:129–137. doi:10.3354/ame01753.

[53] Liu K, Raghavan S, Nelesen S, Linder CR, Warnow T 2009 Rapid and accurate large-scale coestimation of sequence alignments and phylogenetic trees. Science 324:1561–1564. doi:10.1126/science.1171243. 19541996

[54] Wang Q, Garrity GM, Tiedje JM, Cole JR 2007 Naive Bayesian classifier for rapid assignment of rRNA sequences into the new bacterial taxonomy. Appl Environ Microbiol 73:5261–5267. doi:10.1128/AEM.00062-07. 17586664 PMC1950982

[55] Caporaso JG, Kuczynski J, Stombaugh J, Bittinger K, Bushman FD, Costello EK, Fierer N, Peña AG, Goodrich JK, Gordon JI, Huttley GA, Kelley ST, Knights D, Koenig JE, Ley RE, Lozupone CA, McDonald D, Muegge BD, Pirrung M, Reeder J, Sevinsky JR, Turnbaugh PJ, Walters WA, Widmann J, Yatsunenko T, Zaneveld J, Knight R 2010 QIIME allows analysis of high-throughput community sequencing data. Nat Methods 7:335–336. doi:10.1038/nmeth.f.303. 20383131 PMC3156573

[56] Weiss S, Xu ZZ, Peddada S, Amir A, Bittinger K, Gonzalez A, Lozupone C, Zaneveld JR, Vázquez-Baeza Y, Birmingham A, Hyde ER, Knight R 2017 Normalization and microbial differential abundance strategies depend upon data characteristics. Microbiome 5:27. doi:10.1186/s40168-017-0237-y. 28253908 PMC5335496

[57] Ryan CL, Bauman K 2016 Educational attainment in the United States: 2015. Population characteristics. Current population reports. United States Census Bureau, Washington, DC https://www.census.gov/content/dam/Census/library/publications/2016/demo/p20-578.pdf.

[58] Howden LM, Meyer JA 2011 Age and sex composition: 2010. 2010 census briefs. United States Census Bureau, Washington, DC https://www.census.gov/prod/cen2010/briefs/c2010br-03.pdf.

[59] National Center for Health Statistics 2016 Table 58. *In* Health, United States, 2015: with special feature on racial and ethnic health disparities. National Center for Health Statistics, Hyattsville, MD https://www.cdc.gov/nchs/data/hus/2015/058.pdf. 27308685

[60] Gao HX, Regier EE, Close KL 2016 Prevalence of and trends in diabetes among adults in the United States, 1988–2012. J Diabetes 8:8–9. 27144261

[61] Centers for Disease Control and Prevention 2015 Inflammatory bowel disease. Epidemiology of the IBD. Centers for Disease Control and Prevention, Atlanta, GA https://www.cdc.gov/ibd/ibd-epidemiology.htm.

[62] Centers for Disease Control and Prevention 2018 Current cigarette smoking among adults in the United States. Centers for Disease Control and Prevention, Atlanta, GA https://www.cdc.gov/tobacco/data_statistics/fact_sheets/adult_data/cig_smoking/index.htm.

[63] Flores AR, Herman JL, Gates GJ, Brown TNT 2016 How many adults identify as transgender in the United States? The Williams Institute, Los Angeles, CA http://williamsinstitute.law.ucla.edu/wp-content/uploads/How-Many-Adults-Identify-as-Transgender-in-the-United-States.pdf.

[64] Anderson MJ 2001 A new method for non-parametric multivariate analysis of variance. Austral Ecol 26:32–46. doi:10.1111/j.1442-9993.2001.01070.pp.x.

[65] Wold S, Sjöström M, Eriksson L 2001 PLS-regression: a basic tool of chemometrics. Chemometrics Intell Lab Syst 58:109–130. doi:10.1016/S0169-7439(01)00155-1.

[66] Vázquez-Baeza Y, Pirrung M, Gonzalez A, Knight R 2013 EMPeror: a tool for visualizing high-throughput microbial community data. Gigascience 2:16. doi:10.1186/2047-217X-2-16. 24280061 PMC4076506

[67] Bray JR, Curtis JT 1957 An ordination of the upland forest communities of southern Wisconsin. Ecol Monogr 27:325–349. doi:10.2307/1942268.

[68] Mantel N 1967 The detection of disease clustering and a generalized regression approach. Cancer Res 27:209–220. 6018555

[69] Benjamini Y, Hochberg Y 1995 Controlling the false discovery rate: a practical and powerful approach to multiple testing. J R Stat Soc 57:289–300.

[70] Moran PAP 1950 Notes on continuous stochastic phenomena. Biometrika 37:17–23. doi:10.1093/biomet/37.1-2.17. 15420245

[71] Maurice CF, Knowles SCL, Ladau J, Pollard KS, Fenton A, Pedersen AB, Turnbaugh PJ 2015 Marked seasonal variation in the wild mouse gut microbiota. ISME J 9:2423–2434. doi:10.1038/ismej.2015.53. 26023870 PMC4611506

[72] Cramér H 1946 Mathematical methods of statistics (PMS-9). Princeton University Press, Princeton, NJ.

[73] Guo W, Sarkar SK, Peddada SD 2010 Controlling false discoveries in multidimensional directional decisions, with applications to gene expression data on ordered categories. Biometrics 66:485–492. doi:10.1111/j.1541-0420.2009.01292.x. 19645703 PMC2895927

[74] Cohen J 1992 A power primer. Psychol Bull 112:155–159. doi:10.1037/0033-2909.112.1.155. 19565683

[75] Yang JY, Sanchez LM, Rath CM, Liu X, Boudreau PD, Bruns N, Glukhov E, Wodtke A, de Felicio R, Fenner A, Wong WR, Linington RG, Zhang L, Debonsi HM, Gerwick WH, Dorrestein PC 2013 Molecular networking as a dereplication strategy. J Nat Prod 76:1686–1699. doi:10.1021/np400413s. 24025162 PMC3936340

[76] Vázquez-Baeza Y, Gonzalez A, Smarr L, McDonald D, Morton JT, Navas-Molina JA, Knight R 2017 Bringing the dynamic microbiome to life with animations. Cell Host Microbe 21:7–10. doi:10.1016/j.chom.2016.12.009. 28081445

[77] Lloyd-Price J, Mahurkar A, Rahnavard G, Crabtree J, Orvis J, Hall AB, Brady A, Creasy HH, McCracken C, Giglio MG, McDonald D, Franzosa EA, Knight R, White O, Huttenhower C 2017 Strains, functions and dynamics in the expanded Human Microbiome Project. Nature 550:61–66. doi:10.1038/nature23889. 28953883 PMC5831082

[78] Röst HL, Sachsenberg T, Aiche S, Bielow C, Weisser H, Aicheler F, Andreotti S, Ehrlich HC, Gutenbrunner P, Kenar E, Liang X, Nahnsen S, Nilse L, Pfeuffer J, Rosenberger G, Rurik M, Schmitt U, Veit J, Walzer M, Wojnar D, Wolski WE, Schilling O, Choudhary JS, Malmström L, Aebersold R, Reinert K, Kohlbacher O 2016 OpenMS: a flexible open-source software platform for mass spectrometry data analysis. Nat Methods 13:741–748. doi:10.1038/nmeth.3959. 27575624

[79] Shannon P, Markiel A, Ozier O, Baliga NS, Wang JT, Ramage D, Amin N, Schwikowski B, Ideker T 2003 Cytoscape: a software environment for integrated models of biomolecular interaction networks. Genome Res 13:2498–2504. doi:10.1101/gr.1239303. 14597658 PMC403769

[80] Nguyen DD, Melnik AV, Koyama N, Lu X, Schorn M, Fang J, Aguinaldo K, Lincecum TL Jr, Ghequire MGK, Carrion VJ, Cheng TL, Duggan BM, Malone JG, Mauchline TH, Sanchez LM, Kilpatrick AM, Raaijmakers JM, De Mot RD, Moore BS, Medema MH, Dorrestein PC 2016 Indexing the Pseudomonas specialized metabolome enabled the discovery of poaeamide B and the bananamides. Nat Microbiol 2:16197. doi:10.1038/nmicrobiol.2016.197. 27798598 PMC5538791

[81] Dührkop K, Shen H, Meusel M, Rousu J, Böcker S 2015 Searching molecular structure databases with tandem mass spectra using CSI:FingerID. Proc Natl Acad Sci U S A 112:12580–12585. doi:10.1073/pnas.1509788112. 26392543 PMC4611636

[82] GNPS 27 9 2016 GNPS antibiotic use subset. http://gnps.ucsd.edu/ProteoSAFe/status.jsp?task=9bd16822c8d448f59a03e6cc8f017f43.

[83] GNPS 27 9 2016 GNPS plants subset. http://gnps.ucsd.edu/ProteoSAFe/status.jsp?task=d26ae082b1154f73ac050796fcaa6bda.

[84] GNPS 5 12 2017 GNPS isolate supernatant. https://gnps.ucsd.edu/ProteoSAFe/status.jsp?task=23f0f5e5c70f4163b445de71d086d186.

[85] GNPS 5 12 2017 GNPS fecal samples co-networked. https://gnps.ucsd.edu/ProteoSAFe/status.jsp?task=adcfbba9b4ca448f8b2133559b16d954.

[86] Hellinger E 1909 Neue Begründung der Theorie quadratischer Formen von unendlichvielen Veränderlichen. J Reine Angew Math 136:210–271.

[87] Pluskal T, Castillo S, Villar-Briones A, Oresic M 2010 MZmine 2: modular framework for processing, visualizing, and analyzing mass spectrometry-based molecular profile data. BMC Bioinformatics 11:395. doi:10.1186/1471-2105-11-395. 20650010 PMC2918584

[88] Ejigu BA, Valkenborg D, Baggerman G, Vanaerschot M, Witters E, Dujardin JC, Burzykowski T, Berg M 2013 Evaluation of normalization methods to pave the way towards large-scale LC-MS-based metabolomics profiling experiments. OMICS 17:473–485. doi:10.1089/omi.2013.0010. 23808607 PMC3760460

[89] Hackstadt AJ, Hess AM 2009 Filtering for increased power for microarray data analysis. BMC Bioinformatics 10:11. doi:10.1186/1471-2105-10-11. 19133141 PMC2661050

[90] Xia J, Wishart DS 2016 Using MetaboAnalyst 3.0 for comprehensive metabolomics data analysis. Curr Protoc Bioinformatics 55:14.10.1–14.10.91. doi:10.1002/cpbi.11. 27603023

[91] Breiman L 2001 Random forests. Mach Learn 45:5–32. doi:10.1023/A:1010933404324.

[92] Lozupone CA, Hamady M, Kelley ST, Knight R 2007 Quantitative and qualitative beta diversity measures lead to different insights into factors that structure microbial communities. Appl Environ Microbiol 73:1576–1585. doi:10.1128/AEM.01996-06. 17220268 PMC1828774

[93] Pugsley AP, Goldzahl N, Barker RM 1985 Colicin E2 production and release by Escherichia coli K12 and other Enterobacteriaceae. J Gen Microbiol 131:2673–2686. doi:10.1099/00221287-131-10-2673. 3934329

[94] Bankevich A, Nurk S, Antipov D, Gurevich AA, Dvorkin M, Kulikov AS, Lesin VM, Nikolenko SI, Pham S, Prjibelski AD, Pyshkin AV, Sirotkin AV, Vyahhi N, Tesler G, Alekseyev MA, Pevzner PA 2012 SPAdes: a new genome assembly algorithm and its applications to single-cell sequencing. J Comput Biol 19:455–477. doi:10.1089/cmb.2012.0021. 22506599 PMC3342519

[95] Wood DE, Salzberg SL 2014 Kraken: ultrafast metagenomic sequence classification using exact alignments. Genome Biol 15:R46. doi:10.1186/gb-2014-15-3-r46. 24580807 PMC4053813

[96] Nougayrède JP, Homburg S, Taieb F, Boury M, Brzuszkiewicz E, Gottschalk G, Buchrieser C, Hacker J, Dobrindt U, Oswald E 2006 Escherichia coli induces DNA double-strand breaks in eukaryotic cells. Science 313:848–851. doi:10.1126/science.1127059. 16902142

[97] Putze J, Hennequin C, Nougayrède JP, Zhang W, Homburg S, Karch H, Bringer MA, Fayolle C, Carniel E, Rabsch W, Oelschlaeger TA, Oswald E, Forestier C, Hacker J, Dobrindt U 2009 Genetic structure and distribution of the colibactin genomic island among members of the family Enterobacteriaceae. Infect Immun 77:4696–4703. doi:10.1128/IAI.00522-09. 19720753 PMC2772509

[98] Secher T, Samba-Louaka A, Oswald E, Nougayrède JP 2013 Escherichia coli producing colibactin triggers premature and transmissible senescence in mammalian cells. PLoS One 8:e77157. doi:10.1371/journal.pone.0077157. 24116215 PMC3792898

[99] Gurevich A, Mikheenko A, Shlemov A, Korobeynikov A, Mohimani H, Pevzner PA 2018 Increased diversity of peptidic natural products revealed by modification-tolerant database search of mass spectra. Nat Microbiol 3:319–327. doi:10.1038/s41564-017-0094-2. 29358742 PMC5951390

[100] Finn RD, Clements J, Eddy SR 2011 HMMER web server: interactive sequence similarity searching. Nucleic Acids Res 39:W29–W37. doi:10.1093/nar/gkr367. 21593126 PMC3125773

[101] Bates D, Mächler M, Bolker B, Walker S 2015 Fitting linear mixed-effects models using lme4. J Stat Softw 67:1–48. doi:10.18637/jss.v067.i01.

[102] Didion JP, Martin M, Collins FS 2017 Atropos: specific, sensitive, and speedy trimming of sequencing reads. PeerJ 5:e3720. doi:10.7717/peerj.3720. 28875074 PMC5581536

[103] Langmead B, Salzberg SL 2012 Fast gapped-read alignment with Bowtie 2. Nat Methods 9:357–359. doi:10.1038/nmeth.1923. 22388286 PMC3322381

[104] Lu J, Breitwieser FP, Thielen P, Salzberg SL 2017 Bracken: estimating species abundance in metagenomics data. PeerJ Comput Sci 3:e104. doi:10.7717/peerj-cs.104.

[105] Nurk S, Meleshko D, Korobeynikov A, Pevzner PA 2017 metaSPAdes: a new versatile metagenomic assembler. Genome Res 27:824–834. doi:10.1101/gr.213959.116. 28298430 PMC5411777

[106] Seemann T 2014 Prokka: rapid prokaryotic genome annotation. Bioinformatics 30:2068–2069. doi:10.1093/bioinformatics/btu153. 24642063

[107] Wu YW, Simmons BA, Singer SW 2016 MaxBin 2.0: an automated binning algorithm to recover genomes from multiple metagenomic datasets. Bioinformatics 32:605–607. doi:10.1093/bioinformatics/btv638. 26515820

[108] Rho M, Tang H, Ye Y 2010 FragGeneScan: predicting genes in short and error-prone reads. Nucleic Acids Res 38:e191. doi:10.1093/nar/gkq747. 20805240 PMC2978382

[109] Eddy SR 2011 Accelerated profile HMM searches. PLoS Comput Biol 7:e1002195. doi:10.1371/journal.pcbi.1002195. 22039361 PMC3197634

[110] Gibson MK, Forsberg KJ, Dantas G 2015 Improved annotation of antibiotic resistance determinants reveals microbial resistomes cluster by ecology. ISME J 9:207–216. doi:10.1038/ismej.2014.106. 25003965 PMC4274418

[111] Boolchandani M, Patel S, Dantas G 2017 Functional metagenomics to study antibiotic resistance. Methods Mol Biol 1520:307–329. doi:10.1007/978-1-4939-6634-9_19. 27873261

[112] Jiang L, Amir A, Morton JT, Heller R, Arias-Castro E, Knight R 2017 Discrete false-discovery rate improves identification of differentially abundant microbes. mSystems 2:e00092-17. doi:10.1128/mSystems.00092-17. 29181446 PMC5698492

[113] McDonald D, Robbins-Pianka A, Mann AE, Vrbanac A, Amir A, Frazier A, Gonzalez A, Tripathi A, Fahimipour AK, Brennen C, Martino C, Lebrilla C, Lozupone C, Lewis CM, Raison C, Zhang C, Lauber CL, Warinner C, Lowry CA, Callewaert C, Bloss C, Huttenhower C, Knights D, Willner D, Galzerani DD, Gonzalez DJ, Mills DA, Chopra D, Gevers D, Berg-Lyons D, Sears DD, Wendel D, Wolfe E, Lovelace E, Hyde ER, Pierce E, TerAvest E, Montassier E, Bolyen E, Bushman FD, Ackermann G, Wu GD, Church GM, Rahnavard G, Saxe G, Gogul G, Humphrey G, Holscher HD, Ugrina I, German JB, Gregory Caporaso JG, Gilbert J, Wozniak JM, Kerr J, Ravel J, Gaffney J, Lewis JD, Morton JT, Suchodolski JS, Jansson JK, Hampton-Marcell JT, Bobe J, Leach J, Raes J, Green JL, Metcalf JL, Chase JH, Eisen JA, Monk J, Navas-Molina JA, et al. 2018 American Gut Project fecal sOTU counts table. figshare doi:10.6084/m9.figshare.6137192.

[114] McDonald D, Robbins-Pianka A, Mann AE, Vrbanac A, Amir A, Frazier A, Gonzalez A, Tripathi A, Fahimipour AK, Brennen C, Martino C, Lebrilla C, Lozupone C, Lewis CM, Raison C, Zhang C, Lauber CL, Warinner C, Lowry CA, Callewaert C, Bloss C, Huttenhower C, Knights D, Willner D, Galzerani DD, Gonzalez DJ, Mills DA, Chopra D, Gevers D, Berg-Lyons D, Sears DD, Wendel D, Wolfe E, Lovelace E, Hyde ER, Pierce E, TerAvest E, Montassier E, Bolyen E, Bushman FD, Ackermann G, Wu GD, Church GM, Rahnavard G, Saxe G, Gogul G, Humphrey G, Holscher HD, Ugrina I, German JB, Caporaso JG, Gilbert J, Wozniak JM, Kerr J, Ravel J, Gaffney J, Lewis JD, Morton JT, Suchodolski JS, Jansson JK, Hampton-Marcell JT, Bobe J, Leach J, Raes J, Green JL, Metcalf JL, Chase JH, Eisen JA, Monk J, Navas-Molina JA, et al. 2018 American Gut Project fecal sOTU relative abundance table. figshare doi:10.6084/m9.figshare.6137198.

[115] McDonald D, Robbins-Pianka A, Mann AE, Vrbanac A, Amir A, Frazier A, Gonzalez A, Tripathi A, Fahimipour AK, Brennen C, Martino C, Lebrilla C, Lozupone C, Lewis CM, Raison C, Zhang C, Lauber CL, Warinner C, Lowry CA, Callewaert C, Bloss C, Huttenhower C, Knights D, Willner D, Galzerani DD, Gonzalez DJ, Mills DA, Chopra D, Gevers D, Berg-Lyons D, Sears DD, Wendel D, Wolfe E, Lovelace E, Hyde ER, Pierce E, TerAvest E, Montassier E, Bolyen E, Bushman FD, Ackermann G, Wu GD, Church GM, Rahnavard G, Saxe G, Gogul G, Humphrey G, Holscher HD, Ugrina I, German JB, Caporaso JG, Gilbert J, Wozniak JM, Kerr J, Ravel J, Gaffney J, Lewis JD, Morton JT, Suchodolski JS, Jansson JK, Hampton-Marcell JT, Bobe J, Leach J, Raes J, Green JL, Metcalf JL, Chase JH, Eisen JA, Monk J, Navas-Molina JA, et al. 2018 ag_tree.tre. figshare doi:10.6084/m9.figshare.6137270.

[116] McDonald D, Robbins-Pianka A, Mann AE, Vrbanac A, Amir A, Frazier A, Gonzalez A, Tripathi A, Fahimipour AK, Brennen C, Martino C, Lebrilla C, Lozupone C, Lewis CM, Raison C, Zhang C, Lauber CL, Warinner C, Lowry CA, Callewaert C, Bloss C, Huttenhower C, Knights D, Willner D, Galzerani DD, Gonzalez DJ, Mills DA, Chopra D, Gevers D, Berg-Lyons D, Sears DD, Wendel D, Wolfe E, Lovelace E, Hyde ER, Pierce E, TerAvest E, Montassier E, Bolyen E, Bushman FD, Ackermann G, Wu GD, Church GM, Rahnavard G, Saxe G, Gogul G, Humphrey G, Holscher HD, Ugrina I, German JB, Caporaso JG, Gilbert J, Wozniak JM, Kerr J, Ravel J, Gaffney J, Lewis JD, Morton JT, Suchodolski JS, Jansson JK, Hampton-Marcell JT, Bobe J, Leach J, Raes J, Green JL, Metcalf JL, Chase JH, Eisen JA, Monk J, Navas-Molina JA, et al. 2018 Full American Gut Project mapping file. figshare doi:10.6084/m9.figshare.6137315.

[117] McDonald D, Robbins-Pianka A, Mann AE, Vrbanac A, Amir A, Frazier A, Gonzalez A, Tripathi A, Fahimipour AK, Brennen C, Martino C, Lebrilla C, Lozupone C, Lewis CM, Raison C, Zhang C, Lauber CL, Warinner C, Lowry CA, Callewaert C, Bloss C, Huttenhower C, Knights D, Willner D, Galzerani DD, Gonzalez DJ, Mills DA, Chopra D, Gevers D, Berg-Lyons D, Sears DD, Wendel D, Wolfe E, Lovelace E, Hyde ER, Pierce E, TerAvest E, Montassier E, Bolyen E, Bushman FD, Ackermann G, Wu GD, Church GM, Rahnavard G, Saxe G, Gogul G, Humphrey G, Holscher HD, Ugrina I, German JB, Caporaso JG, Gilbert J, Wozniak JM, Kerr J, Ravel J, Gaffney J, Lewis JD, Morton JT, Suchodolski JS, Jansson JK, Hampton-Marcell JT, Bobe J, Leach J, Raes J, Green JL, Metcalf JL, Chase JH, Eisen JA, Monk J, Navas-Molina JA, et al. 2018 American Gut collated alpha diversities. figshare doi:10.6084/m9.figshare.6137312.

[118] McDonald D, Robbins-Pianka A, Mann AE, Vrbanac A, Amir A, Frazier A, Gonzalez A, Tripathi A, Fahimipour AK, Brennen C, Martino C, Lebrilla C, Lozupone C, Lewis CM, Raison C, Zhang C, Lauber CL, Warinner C, Lowry CA, Callewaert C, Bloss C, Huttenhower C, Knights D, Willner D, Galzerani DD, Gonzalez DJ, Mills DA, Chopra D, Gevers D, Berg-Lyons D, Sears DD, Wendel D, Wolfe E, Lovelace E, Hyde ER, Pierce E, TerAvest E, Montassier E, Bolyen E, Bushman FD, Ackermann G, Wu GD, Church GM, Rahnavard G, Saxe G, Gogul G, Humphrey G, Holscher HD, Ugrina I, German JB, Caporaso JG, Gilbert J, Wozniak JM, Kerr J, Ravel J, Gaffney J, Lewis JD, Morton JT, Suchodolski JS, Jansson JK, Hampton-Marcell JT, Bobe J, Leach J, Raes J, Green JL, Metcalf JL, Chase JH, Eisen JA, Monk J, Navas-Molina JA, et al. 2018 Unweighted UniFrac distances. figshare doi:10.6084/m9.figshare.6131024.

[119] McDonald D, Robbins-Pianka A, Mann AE, Vrbanac A, Amir A, Frazier A, Gonzalez A, Tripathi A, Fahimipour AK, Brennen C, Martino C, Lebrilla C, Lozupone C, Lewis CM, Raison C, Zhang C, Lauber CL, Warinner C, Lowry CA, Callewaert C, Bloss C, Huttenhower C, Knights D, Willner D, Galzerani DD, Gonzalez DJ, Mills DA, Chopra D, Gevers D, Berg-Lyons D, Sears DD, Wendel D, Wolfe E, Lovelace E, Hyde ER, Pierce E, TerAvest E, Montassier E, Bolyen E, Bushman FD, Ackermann G, Wu GD, Church GM, Rahnavard G, Saxe G, Gogul G, Humphrey G, Holscher HD, Ugrina I, German JB, Caporaso JG, Gilbert J, Wozniak JM, Kerr J, Ravel J, Gaffney J, Lewis JD, Morton JT, Suchodolski JS, Jansson JK, Hampton-Marcell JT, Bobe J, Leach J, Raes J, Green JL, Metcalf JL, Chase JH, Eisen JA, Monk J, Navas-Molina JA, et al. 2018 Weighted normalized UniFrac distances. figshare doi:10.6084/m9.figshare.6133121.

[120] Cohen LJ, Kang HS, Chu J, Huang YH, Gordon EA, Reddy BVB, Ternei MA, Craig JW, Brady SF 2015 Functional metagenomic discovery of bacterial effectors in the human microbiome and isolation of commendamide, a GPCR G2A/132 agonist. Proc Natl Acad Sci U S A 112:E4825–E4834. doi:10.1073/pnas.1508737112. PMC4568208 26283367

