# Supplementary material for: American Gut: an Open Platform for Citizen Science Microbiome Research
Source: mSystems. 2018 May 15;3(3):e00031-18. doi: 10.1128/mSystems.00031-18 (PMC5954204; doi:10.1128/mSystems.00031-18)
Supplement: TEXT S1 [file sys003182229s1.docx]

**Supplemental**

**Effect size comparisons**

**Multi-cohort replication detail**

**Projects using the American Gut infrastructure**

**American Gut Survey**

**Supplemental references**

**Multi-cohort replication detail**

Results. Our cohort consists of 250 American Gut Project participants (122 USA residents, 112 UK, 2 Canada, 6 Switzerland, 2 Germany, 2 France and 4 Australia) -- 152 females and 98 males. Of the clinical population, 97% endorsed depression, 10% PTSD, 4% bipolar disorder, and 2% schizophrenia (some individuals endorsed more than one disorder). There was significant community level separation between patients and NCs using weighted UniFrac (*p*=0.05, *pseudo-F*=2.36) and Bray-Curtis (*p*=0.05, *pseudo-F*=1.36). Three sequencing plate pair was significantly different using weighted UniFrac (FDR-corrected *p* between 0.02 and 0.03). However, case-control distribution on each of those plates were equal, so we have not discarded them. No significant plate-to-plate differences were observed using Bray-Curtis dissimilarity and in all cases *pseudo-F* values were smaller to those reported for mental illness to NC differences.

This difference is notable as groups were 1:1 matched on various demographic factors and clinical conditions known to be present in higher rates among psychiatric patients, which can impact microbial composition.

As reported in the main text, microbiome differences between countries are a major driver of separation. Hence, we decided to perform additional analyses only on the largest groups, i.e. USA and UK. Repeating the previous analysis only on USA and UK residents confirms our previous finding with an increased level of confidence - Bray-Curtis *p*=0.03

weighted UniFrac *p*<0.05. It suggests that country-to-country differences likely obscure the signal.

To follow-up on that, we analysed UK and USA residents separately. Looking at a subset including only UK residents we observe a significant separation both in Bray-Curtis (*p*=0.01, *pseudo-F*=1.63) and weighted UniFrac (*p*=0.05, *pseudo-F*=2.16) and no significant plate-to-plate variation.Within this cohort we were also able to find 2 differentially abundant sOTUs differentiating cases and controls. One belonging to family Ruminococcaceae and the other to phylum Tenericutes, both are more prevalent in NC subjects (table S3). Interestingly, family Ruminococcaceae has been reported in the literature as associated with major depressive disorder (MDD). A study on a cohort from eastern China reported this family to be depleted in MDD subjects [(1)](https://paperpile.com/c/zODTm0/LB7H), while a study from central China reported this family to be over-represented in MDD subjects [(2)](https://paperpile.com/c/zODTm0/GrVC). Furthermore, an unclassified member of the family Ruminococcaceae was also found to be decreased in bipolar disorder [(3)](https://paperpile.com/c/zODTm0/ImlOr).

For USA only, we similarly observe a significant weighted UniFrac separation (*p*=0.05, *pseudo-F*=2.58), but lose significance in Bray-Curtis dissimilarity (*p*>0.1). A likely reason for this effect, is that the UK represents a more geographically homogenous cohort, while for US citizens we observe a wide geographic spread between subjects (West 60, Northeast 30, South 18, Midwest 14).

We also attempted replicating our findings looking at some of the selected matching criteria. Splitting our cohort by sex resulted in a significant result for females (weighted UniFrac *p*=0.04; *pseudo-F* = 2.35), but not for males. The likely reason being not enough statistical power in this group (98 participants). We were also able to replicate this finding for the bottom half of the population by age (122 subjects up to 45 years; as in the main text) finding weighted UniFrac (*p*=0.05; *pseudo-F* = 2.45) splitting subjects by mental health status.

Discussion. Mental health status questions have only been part of the AGP questionnaire since late 2015. Despite this limited time of availability given the length of the project, we were able to accumulate significant evidence of associations between mental illness and the microbiome and perform a multi-cohort replication. These findings are among the first few multi-national studies to provide evidence for altered gut microbiota in severe mental illness. Global community differences were observed between participants who endorsed a mental illness and NCs. Notably, these differences were observed after controlling for comorbid medical conditions known to be common in psychiatric disorders, which are also risk factors for microbial abnormalities. This study is among one of the largest investigations of the gut microbiome to date in psychiatric populations. A notable strength of these findings is that effects are seen across different psychiatric diagnoses. Furthermore, these results indicate the potential to draw clinically-relevant conclusions from self-reported, crowd-sourced data. Increased noise in samples (e.g., from shipping samples) and errors/variability in respondent metadata were minimized by using careful case-control matching accounting for confounding factors. This is one of the first clinical investigations of the gut-brain axis in which samples were not restricted by geographical region or research institution and were collected across the US, UK, and other countries.

**Effect size comparisons**

Although technical variables are present in the effect size results, we note that many of these variables are nested. For instance, mastermix_lot in general varied with the processing round (i.e., center_project_name), but in some instances, the same lot was used for multiple rounds or multiple lots were used within a round. While strongly correlated with the sequencing round, it fell into a separate cluster when assessing cross correlation between metadata variables. It is clear technical variables show non-trivial effects in the data, but expected signals (e.g., age, BMI, etc) including subtle effects (e.g., sleep duration, alcohol consumption) are still easily detectable despite these technical effects. Critically, many of these effects, including some subtle ones, replicate results reported in Falony *et al* 2016 [(4)](https://paperpile.com/c/zODTm0/4WnYn); for example, alcohol had a stronger effect than sleep in our population. To test whether the ranking was correlated, we first computed a Spearman correlation between rankings for the two populations in figure 3 (FGFP and LLDeep [(5)](https://paperpile.com/c/zODTm0/T2OAI)) of Falony *et al*, and observed *r*=0.06 *p*=0.78, suggesting that rank order of variables may be impacted by study population and/or details of survey design and translation. We then computed the Spearman correlation between the weighted UniFrac effects in our study and our best-identified matches to variables common with Falony *et al* and LLDeep (table S2, observing *r*=-0.39 *p*=0.25 with FGFP and *r*=0.61 *p*=0.077 with LLDeep). The concept that as population studies accumulate, commonality in effect size ranking will be observed, therefore seems encouraging.

With the variation analysis, and similar to the effect size observations, we tested whether the rankings in the Bray Curtis results were correlated using Spearman with either FGFP or LLDeep, observing r=-0.33 p=0.41 with FGFP and r=0.61 p=0.15 with LLDeep.

**Projects using the American Gut infrastructure.**

The American Gut Project has raised over $2,000,000 USD, with contributions to the project now being made through FundRazr (*http://fundrazr.com*). Early in the project, and after launch, we began to get international participants but their participation required a large shipping expense (e.g., $500 USD from Australia). In order to accomodate international participants, we began working on establishing a network of sites to allow for bulk shipment of samples. In October 2014, a sister project, the British Gut (*http://britishgut.org/*), was launched in the United Kingdom (UK), while aggregation sites for forwarding samples have been established in Australia and Singapore. These successful sites are providing paradigms for international expansion, which are continuing to be explored.

One attractive component of using crowdsourcing for sample collection via the AGP is the ability to assemble large cohorts (or expand upon existing ones) using targeted subproject inclusion. By empowering citizen scientists to participate directly in ongoing experiments -- and pay for a portion of experimental costs for sample analysis -- the cost and required manpower of assembling the necessary cohorts for microbiome studies is vastly reduced. The AGP has collected an unprecedented number of samples using a crowdsourcing model that will continue to be scalable for some time. It is also possible to leverage this infrastructure to collect samples from specialized cohorts by targeted subproject inclusion. The feasibility of this is demonstrated by incorporation of our Intensive Care Unit (ICU) [(6)](https://paperpile.com/c/zODTm0/u0COM), Office Microbial Community Establishment study (OMCE) [(7)](https://paperpile.com/c/zODTm0/fu407), Autism Spectrum Disorder (ASD), Mind & Microbiome (M&M), and UC San Diego student athlete pilot programs (see below).

Office Microbial Community Establishment. We recently conducted a study at Northern Arizona University where we explored the spatial and temporal aspects of microbial community establishment in the office environment by monitoring bacterial, archaeal, and fungal communities in nine offices in Flagstaff, San Diego, and Toronto. Samples were taken from the offices approximately every other day over the course of a year. Bacterial 16S rRNA and the fungal internal transcribed spacer 1 (ITS1) were amplified and sequenced in order to track the composition and dynamics of these communities in the offices starting from UV sterilized building materials. Throughout the course of this study, detailed building and environmental parameters were recorded in order to understand the abiotic factors that might drive the composition of microbial communities. However, we suspect that biotic factors, namely the office inhabitants, are also likely driving community establishment. Through the AGP, we tracked the skin, oral, and fecal microbiomes of human inhabitants of our selected offices. The volunteers coordinated directly with the American Gut Project, recording personal data and sample identifiers through the project’s website. The human subject component of this study was completed using the AGP pipeline under the approval of the AGP IRB, and the PIs on the office study will only receive de-identified sample metadata and sequence data for analysis. The AGP thus provided the infrastructure to incorporate a human component into this study, which would have been impractical otherwise as it would have been a costly side-endeavor.

ICU Pilot. The gut is hypothesized to play a central role in the progression of critical illness, sepsis and multiple organ dysfunction syndrome [(8)](https://paperpile.com/c/zODTm0/AuPCA). Recent clinical evidence indicates alteration of gut microbiome is associated with infectious complications and mortality in ICU patients [(9)](https://paperpile.com/c/zODTm0/HJMXq). To date, evaluations of the microbial ecology in the ICU have been restricted to small culture-based studies. These limited studies frequently demonstrate that ICU patients are rapidly colonized with opportunistic pathogens and suffer significant loss of microbial diversity, which has been suggested to be associated with poor outcomes [(9)](https://paperpile.com/c/zODTm0/HJMXq). Further, several studies have demonstrated that the effects of commonly used broad-spectrum antibiotics (common in ICU) on the microbiota can be long lasting, with effects lasting weeks to years [(10)](https://paperpile.com/c/zODTm0/Amt3F). However, the fate of commensal organisms in the ICU, which serve beneficial purposes, is poorly understood. For this reason, a trial with prospective monitoring of the ICU microbiome with comprehensive culture-independent techniques was needed. To address this question, samples were collected from ICU patients who were expected to remain in the ICU > 72 hours at five different ICUs (4 Centers in North America). Data were collected on ICU outcomes, antibiotic use, and nutrition intake in conjunction with the Canadian International Critical Care Nutrition Survey (www.criticalcarenutrition.com) and the Translational Pharmacology of Nutrition Program at the University of Colorado School of Medicine. Patients were not excluded from the pilot based on health status. Fecal samples were collected within 72 hours of admission to the ICU, and at ten days or discharge. These samples were run together with American Gut samples in accordance with the methods described in this manuscript. The results [(6)](https://paperpile.com/c/zODTm0/u0COM) of this study begin to address fundamental questions with regards to the impact of critical illness and ICU nutrition delivery on the microbiome. We hope further data on the prognostic role of microbiome alterations on clinical outcome will be generated from these results as analysis continues. This may help guide future interventions via probiotic, prebiotic, fecal transplant therapy and nutrition interventions to reconstitute a normal, potentially beneficial microbiome following critical illness.

ASD Cohort. The ASD cohort was established to increase the prevalence of ASD patients and matched controls in the AGP population. The aim was to recruit 500 ASD patients and ideally 500 matched sibling controls (neurotypical siblings), or parent controls. The aim of this control group was to attain some genetic lineage control, as well as enabling children/adults from the same household to be assessed for microbial similarity. The hypothesis that the microbiome of ASD patients may be influenced by the microbiome of the home environment could therefore be tested. Comparison of matched controls to the rest of the AGP population allows for assessment of variables that delineate microbial similarity, e.g. age, diet, geographic location, medical history, etc. This comparison then allows for a more detailed investigation of the ASD-control comparative analysis, so that individual bacterial taxa that are statistically enriched or reduced in the ASD cohort can be co-analyzed against taxa that are statistically enriched or reduced in the neurotypical cohort against the AGP population. This study is performed in collaboration with other ongoing ASD population cohorts at the University of Colorado School of Medicine and Stanford, and provides a platform for continued expansion of this important cohort.

M&M Cohort. The goal of the M&M cohort is to survey the gut microbiome generally for associations with mental health, and is in collaboration with Northeastern University (NU). At NU, enrollment is ongoing in which participants come to the lab to take a broad psychological battery of standardized psychological surveys and donate stool samples, which are collected using the AGP kits and processed using the AGP sequencing pipeline. The M&M cohort dataset currently has 267 participants, and data analysis is underway. The goal of this project is to serve as a foundation for other comparative studies of the gut microbiome and human psychology.

UC San Diego Student Athlete Pilot**.** Several studies have explored the effects of exercise on the gut microbiome in both animal models and in humans. Germ-free mice were reported to have reduced swimming endurance and reduced presence of antioxidants (which may protect against exercise-induced oxidative damage) than specific pathogen free (SPF) mice [(11)](https://paperpile.com/c/zODTm0/9rQfy). In professional college rugby players [(12)](https://paperpile.com/c/zODTm0/JKreG) and in soldiers undergoing a multi-day march [(13)](https://paperpile.com/c/zODTm0/gbo21), gut microbiome composition and metabolism were altered; however, while rugby players had reduced markers of inflammation and improved metabolic profiles, the opposite was observed in the soldiers, indicating that stress level and duration likely play a key role in microbiome-exercise associations. Nevertheless, current studies are only beginning to unravel the athlete microbiome and its precise relationship with diet, training, performance, and recovery. To further characterize the athlete microbiome, including to identify sport-specific differences, we have partnered with UC San Diego Athletics to sequence the gut microbiomes of all athletes on the UC San Diego campus who desire to participant, representing an available pool size of up to 1500 athletes engaging in 60 different sports. We are continuing to receive athletes into the cohort, which currently consists of 67 athletes from 14 sports (men’s and women’s track, men’s and women’s basketball, men’s soccer, men’s and women’s swimming, men’s tennis, men’s and women’s volleyball, men’s water polo, softball, women’s rowin, and men’s fencing).

**American Gut Survey:**

**American Gut Project**

GENERAL QUESTIONNAIRE

***You may decline to answer any question.***

***Personal and Contact Information***

Name: Today’s Date:

Gender: Birth Date: (MM/YY)

Height: Weight:

Country of birth: Current ZIP code:

Country of residence:

***General Diet Information***

1.) How would you classify your diet?

a. I eat anything with no exclusions (omnivore)

b. I eat anything except red meat

c. Vegetarian

d. Vegetarian but eat seafood

e. Vegan

2.) Are you taking a daily multivitamin?

a. Yes

b. No

3.) How frequently do you take a probiotic?

a. Never

b. Rarely (a few times/month)

c. Occasionally (1-2 times/week)

d. Regularly (3-5 times/week)

e. Daily

4.) How frequently do you take Vitamin B complex, folate or folic acid?

a. Never

b. Rarely (a few times/month)

c. Occasionally (1-2 times/week)

d. Regularly (3-5 times/week)

e. Daily

5.) How frequently do you take a Vitamin D supplement?

a. Never

b. Rarely (a few times/month)

c. Occasionally (1-2 times/week)

d. Regularly (3-5 times/week)

e. Daily

6.) Are you taking any other nutritional/herbal supplements?

a.Yes** *Please indicate what you are taking in the appropriate space in the supplemental answer section*

b. No

7.) Do you eat a specialized diet ? (select all that apply):

a. paleo-diet or primal diet,

b. modified paleo diet,

c. raw food diet,

d. FODMAP,

e. Westen-Price, or other low-grain, low processed food diet

f. Kosher,

g. Halaal,

h. Exclude nightshades,

i. Exclude dairy,

j. Exclude refined sugars,

k. Other restrictions not described here,

l. I do not eat a specialized diet.

8.) Do you eat meat/dairy products from animals treated with antibiotics?

a. Yes

b. No

c. Not Sure

9.) Do you follow any other special diet restrictions other than those indicated above?

a. Yes** *Please explain in the supplemental answer section*

b. No

10.) What is your drinking water source at home?

a. City

b. Well

c. Bottled

d. Filtered

e. Not sure

***General Information***

11.) What is your race/ethnicity?

a. Caucasian

b. Asian or Pacific Islander

c. African American

d. Hispanic

e. Other** *Please explain in the supplemental answer section*

12.) When did you move to your current state of residence?

a. Within the past month

b. Within the past 3 months

c. Within the past 6 months

d. Within the past year

e. I have lived in my current state of residence for more than a year.

13.) I have traveled outside of the United States in the past _________. (***If you answered a, b, or c,* *please explain in the supplemental answer section*

a. Month**

b. 3 months**

c. 6 months**

d. 1 year

e. I have not been outside of the United States in the past year.

14.) How many non-family roommates do you have?

a. None

b. One

c. Two

d. Three

e. More than three

15.) Are any of your roommates participating in this study?

a. Yes** *Please explain in the supplemental answer section*

b. No

c. Not sure

16.) Are you related to or live with any of the other participants in this study?

a. Yes

b. No

c. Not sure

17.) Do you have a dog(s)?

a. Yes** *Please explain in the supplemental answer section*

b. No

18.) Do you have a cat(s)?

a. Yes** *Please explain in the supplemental answer section*

b. No

19.) Do you have other pet(s)at home:

a. Yes** *Please explain in the supplemental answer section*

b. No

20.) Which is your dominant hand?

a. I am right handed

b. I am left handed

c. I am ambidextrous

21.) What is your highest level of education?

a. Did not complete high school

b. High School or GED equivalent

c. Some college or technical school

d. Associate’s degree

e. Bachelor’s degree

f. Some graduate school or professional

g. Graduate or Professional degree

***General Lifestyle and Hygiene Information***

1.) How often do you exercise?

a. Daily

b. Regularly (3-5 times/week)

c. Occasionally (1-2 times/week)

d. Rarely (few times/month)

e. Never

2.) Do you generally exercise indoors or outdoors?

a. Indoors

b. Outdoors

c. Both

d. Depends on the season

e. None of the above

3.) Do you bite your fingernails?

a. Yes

b. No

4.) How often do you use a swimming pool/hot tub?

a. Daily

b. Regularly (3-5 times/week)

c. Occasionally (1-2 times/week)

d. Rarely (few times/month)

e. Never

5.) How often do you smoke cigarettes?

a. Daily

b. Regularly (3-5 times/week)

c. Occasionally (1-2 times/week)

d. Rarely (few times/month)

e. Never

6.) How often do you drink alcohol?

a. Daily

b. Regularly (3-5 times/week)

c. Occasionally (1-2 times/week)

d. Rarely (few times/month)

e. Never

7.) What type(s) of alcohol do you typically consume (select all that apply)?

a. Beer/Cider

b. Sour beers

c. White wine

d. Red wine

e. Spirits/hard alcohol

8.) How many alcoholic drinks do you usually have when you do drink?

a. 1

b. 1-2

c. 2-3

d. 3-4

e. 4+

f. I don't drink

9.) How often do you brush your teeth?

a. 2+ times/day

b. 1-2 times/day

c. Once a day

d. Never

10.) How often do you floss your teeth?

a. Daily

b. Regularly (3-5 times/week)

c. Occasionally (1-2 times/week)

d. Rarely (few times/month)

e. Never

11.) How often do you wear facial cosmetics?

a. Daily

b. Regularly (3-5 times/week)

c. Occasionally (1-2 times/week)

d. Rarely (few times/month)

e. Never

12.) Do you use deodorant or antiperspirant (antiperspirants generally contain aluminum)?

a. I use deodorant

b. I use an antiperspirant

c. Not sure, but I use some form of deodorant/antiperspirant

d. I do not use deodorant or an antiperspirant

13.) Approximately how many hours of sleep to you get in an average night?

a. Less than 5 hours

b. 5-6 hours

c. 6-7 hours

d. 7-8 hours

e. 8 or more hours

14.) Do you use fabric softener when drying your clothes?

a. Yes

b. No

***If you consider yourself a regular surfer, click here to volunteer more information about this activity (Link to surfer questionnaire)***

***General Health Information***

15.) How many times do you have a bowel movement in an average day?

a. Less than one

b. One

c. Two

d. Three

e. Four

f. Five or more

16.) Describe the quality of your bowel movements:

a. I tend to be constipated (have difficulty passing stool)- Type 1 and 2;

b. I tend to have diarrhea (watery stool) – Type 5, 6 and 7;

c. I tend to have normal formed stool – Type 3 and 4.

17.) I have taken antibiotics in the last ____________. (*If you answered a or b, please indicate which antibiotic you took and what you were treating in the supplemental answer section*

a. Week**

b. Month**

c. 6 months

d. Year

e. I have not taken antibiotics in the past year.

18.) I have received a flu vaccine in the last ____________.

a. Week

b. Month

c. 6 months

d. Year

e. I have not gotten the flu vaccine in the past year.

19.) Are you currently using some form of hormonal birth control?

a. Yes, I am taking the “pill”

b. Yes, I use an injected contraceptive (*DMPA*)

c. Yes, I use a contraceptive patch (*Ortho-Evra*)

d. Yes, I use the *NuvaRing*

e. Yes, I use a hormonal IUD (*Mirena*)

f. No

20.) Are you currently pregnant?

a. Yes** *Please indicate your due date in the supplemental answer section*

b. No

c. Not sure

21.) My weight has _________ within the last 6 months.

a. Increased more than 10 pounds

b. Decreased more than 10 pounds

c. Remained stable

22.) Have you had your tonsils removed?

a. Yes

b. No

c. Not sure

23.) Have you had your appendix removed?

a. Yes

b. No

c. Not sure

24.) Have you had chickenpox?

a. Yes

b. No

c. Not sure

25.) Do you currently take prescription medication for facial acne?

a. Yes

b. No

26.) Do you use over the counter products to control facial acne?

a. Yes

b. No

27.) Do you currently take over the counter or prescription medication for other conditions?

a. Yes*** Please explain in the supplemental answer section*

b. No

28.) Were you born via caesarean section (C-section)?

a. Yes

b. No

c. Not sure

29.) How were you fed as an infant?

a. Primarily breast milk

b. Primarily infant formula

c. A mixture of breast milk and formula

d. Not sure

30.) Have you been diagnosed with ADD/ADHD?

a. I do not have this condition

b. Diagnosed by a medical professional (doctor, physician assistant)

c. Diagnosed by an alternative medicine practitioner

d. Self-diagnosed

31.) Have you been diagnosed with Alzheimer’s Disease/Dementia

a. I do not have this condition

b. Diagnosed by a medical professional (doctor, physician assistant)

c. Diagnosed by an alternative medicine practitioner

d. Self-diagnosed

32.) Have you been diagnosed with Asthma, Cystic fibrosis, COPD or other lung Disease?

a. I do not have this condition

b. Diagnosed by a medical professional (doctor, physician assistant)

c. Diagnosed by an alternative medicine practitioner

d. Self-diagnosed

33.) Have you been diagnosed with Autism or Autism Spectrum Disorder?

a. I do not have this condition

b. Diagnosed by a medical professional (doctor, physician assistant)

c. Diagnosed by an alternative medicine practitioner

d. Self-diagnosed

***If you answered b, c or d, click here to volunteer more information about this condition (link to ASD questionnaire).***

34.) Have you been diagnosed with autoimmune disease such as Lupus (systemic lupus erythematosus), R.A. (rheumatoid arthritis), MS (multiple sclerosis), Hashimoto’s thyroiditis , or any other auto-immune disease?

a. I do not have this condition

b. Diagnosed by a medical professional (doctor, physician assistant)

c. Diagnosed by an alternative medicine practitioner

d. Self-diagnosed

35.) Have you ever been diagnosed with Candida or fungal overgrowth in the gut?

a. I do not have this condition

b. Diagnosed by a medical professional (doctor, physician assistant)

c. Diagnosed by an alternative medicine practitioner

d. Self-diagnosed

36.) Have you ever been diagnosed with *Clostridium difficile* (*C. diff*) infection?

a. I do not have this condition

b. Diagnosed by a medical professional (doctor, physician assistant)

c. Diagnosed by an alternative medicine practitioner

d. Self-diagnosed

37.) Have you ever been diagnosed with coronary artery disease, heart disease, heart attack, stroke?

a. I do not have this condition

b. Diagnosed by a medical professional (doctor, physician assistant)

c. Diagnosed by an alternative medicine practitioner

d. Self-diagnosed

38.) Have you ever been diagnosed with mental health illness?

a. No

b. Yes

39.) If you responded “yes” to question 37.) please select which disorder(s) from the following list:

a. Depression

b. Bipolar disorder

c. PTSD (post-traumatic stress disorder)

d. Schizophrenia

e. Anorexia nervosa

f. Bulimia nervosa

g. Substance abuse

40.) Have you ever been diagnosed with diabetes?

a. I do not have this condition

b. Diagnosed by a medical professional (doctor, physician assistant)

c. Diagnosed by an alternative medicine practitioner

d. Self-diagnosed

41.) If you responded “Yes” to question 40.), which type of diabetes:

a. Prediabetes

b. Type I diabetes

c. Type II diabetes

d. Gestational diabetes

42.) Have you ever been diagnosed with epilepsy or seizure disorder?

a. I do not have this condition

b. Diagnosed by a medical professional (doctor, physician assistant)

c. Diagnosed by an alternative medicine practitioner

d. Self-diagnosed

43.) Have you ever been diagnosed with irritable bowel syndrome (IBS)?

a. I do not have this condition

b. Diagnosed by a medical professional (doctor, physician assistant)

c. Diagnosed by an alternative medicine practitioner

d. Self-diagnosed

44.) Have you ever been diagnosed with inflammatory bowel disease (IBD)?

a. I do not have this condition

b. Diagnosed by a medical professional (doctor, physician assistant)

c. Diagnosed by an alternative medicine practitioner

d. Self-diagnosed

45.) If you answered b, c, or d to question 43.), which type of IBD do you have?

a. Ileal Crohn’s disease

b. Colonic Crohn’s disease

c. Ileal and Colonic Crohn’s disease

d. Ulcerative Colitis

e. Microcolitis

46.) Have you ever been diagnosed with migraines?

a. I do not have this condition

b. Diagnosed by a medical professional (doctor, physician assistant)

c. Diagnosed by an alternative medicine practitioner

d. Self-diagnosed

***If you answered b, c or d, click here to volunteer more information about this condition (Link to migraine questionnaire)***

47.) Have you ever been diagnosed with kidney disease?

a. I do not have this condition

b. Diagnosed by a medical professional (doctor, physician assistant)

c. Diagnosed by an alternative medicine practitioner

d. Self-diagnosed

48.) Have you ever been diagnosed with liver disease?

a. I do not have this condition

b. Diagnosed by a medical professional (doctor, physician assistant)

c. Diagnosed by an alternative medicine practitioner

d. Self-diagnosed

49.) Have you ever been diagnosed with phenylketonuria?

a. I do not have this condition

b. Diagnosed by a medical professional (doctor, physician assistant)

c. Diagnosed by an alternative medicine practitioner

d. Self-diagnosed

50.) Have you ever been diagnosed with small intestinal bacterial overgrowth (SIBO)?

a. I do not have this condition

b. Diagnosed by a medical professional (doctor, physician assistant)

c. Diagnosed by an alternative medicine practitioner

d. Self-diagnosed

51.) Have you ever been diagnosed with a skin condition?

a. I do not have this condition

b. Diagnosed by a medical professional (doctor, physician assistant)

c. Diagnosed by an alternative medicine practitioner

d. Self-diagnosed

52.) Have you ever been diagnosed with thyroid disease?

a. I do not have this condition

b. Diagnosed by a medical professional (doctor, physician assistant)

c. Diagnosed by an alternative medicine practitioner

d. Self-diagnosed

53.) Have you ever been diagnosed with any other relevant clinical condition? ** *Please enter in the supplemental answer section*

a. I do not have this condition

b. Diagnosed by a medical professional (doctor, physician assistant)

c. Diagnosed by an alternative medicine practitioner

d. Self-diagnosed

54.) Are you willing to be contacted to answer additional questions about the conditions listed above?

a. Yes

b. No

55.) Do you have seasonal allergies?

a. Yes

b. No

56.) Do you have any of the following non-food allergies? (check all that apply)

a. Drug (e.g. Penicillin)

b. Pet dander

c. Beestings

d. Poison ivy/oak

e. Sun

57.) Are you lactose intolerant?

a. Yes

b. No

58.) Are you gluten intolerant?

a. I was diagnosed with celiac disease

b. I was diagnosed with gluten allergy (anti-gluten IgG), but not celiac disease

c. I do not eat gluten because it makes me feel bad

d. No

59.) I am allergic to:__ (mark all that apply)

a. Peanuts

b. Tree nuts

c. Shellfish

d. Other

e. I have no food allergies that I know of.

60.) Do you have vivid and/or frightening dreams?

a. Never

b. Rarely (less than once/week)

c. Occasionally (1-2 times/week)

d. Regularly (3-5times/week)

e. Daily

***Detailed Dietary information***

61.) Are you an infant who receives most of their nutrition from breast milk or formula, or an adult who receives most (more than 75% of daily calories) of their nutrition from adult nutritional shakes (i.e. Ensure)?

a. Yes

b. No

c. I eat both solid food and formula/breast milk

62.) In an average week, how often do you consume meat/eggs?

a. Never

b. Rarely (less than once/week)

c. Occasionally (1-2 times/week)

d Regularly (3-5 times/week)

e. Daily

63.) In an average week, how often do you cook and consume home cooked meals? (Exclude ready-to-eat meals like boxed macaroni and cheese, ramen noodles, lean cuisine)

a. Never

b. Rarely (less than once/week)

c. Occasionally (1-2 times/week)

d. Regularly (3-5 times/week)

e. Daily

64.) In an average week, how often do you consume ready-to-eat meals (i.e macaroni and cheese, ramen noodles, lean cuisine)?

a. Never

b. Rarely (less than once/week)

c. Occasionally (1-2 times/week)

d. Regularly (3-5 times/week)

e. Daily

65.) In an average week, how often do you eat food prepared at a restaurant, including carry-out/take-out?

a. Never

b. Rarely (less than once/week)

c. Occasionally (1-2 times/week)

d. Regularly (3-5 times/week)

e. Daily

***Grains/Fiber***

66.) In an average week, how often do you eat at least 2 servings of whole grains in a day?

(1 serving = 1 slice of 100% whole grain bread; 1 cup whole grain cereal like Shredded Wheat, Wheaties, Grape Nuts, high fiber cereals, or oatmeal; 3-4 whole grain crackers; ½ cup brown rice or whole wheat pasta)

a. Never

b. Rarely (less than once/week)

c. Occasionally (1-2 times/week)

d. Regularly (3-5 times/week)

e. Daily

***Fruits and Vegetables***

67.) In an average week, how often to you consume at least 2-3 servings of fruit in a day? (1 serving = ½ cup fruit; 1 medium sized fruit; 4 oz. 100% fruit juice.)

a. Never

b. Rarely (less than once/week)

c. Occasionally (1-2 times/week)

d. Regularly (3-5 times/week)

e. Daily

68.) In an average week, how often do you consume at least 2-3 servings of vegetables, including potatoes in a day? (1 serving = ½ cup vegetables/potatoes; 1 cup leafy raw vegetables)

a. Never

b. Rarely (less than once/week

c. Occasionally (1-2 times/week)

d. Regularly (3-5 times/week)

e. Daily

69.) In an average week, how many different plants do you eat?

a. <5

b. 6-10

c. 11-20

d. 21-30

e. >30

e.g. If you consume a can of soup that contains carrots, potatoes and onion, you can count this as 3 different plants; If you consume multi-grain bread, each different grain counts as a plant. Include all fruits in the total.

***Fermented Foods***

70.) How often do you consume one or more servings of fermented vegetables or plant products a day in an average week? (1 serving = ½ cup sauerkraut, kimchi or fermented vegetable or 1 cup of kombucha)

a. Never

b. Rarely (less than once/week

c. Occasionally (1-2 times/week)

d. Regularly (3-5 times/week)

e. Daily

***If you consume fermented foods (including plant, grain, dairy, and meat-based products) more than once or twice a week, click to volunteer more information about this (Link to fermented foods questionnaire).***

***Dairy***

71.) In an average week, how often do you consume at least 2 servings of milk or cheese a day? (1 serving = 1 cup milk or yogurt; 1½ - 2 ounces cheese)

a. Never

b. Rarely (less than once/week

c. Occasionally (1-2 times/week)

d. Regularly (3-5 times/week)

e. Daily

72.) In an average week, how often do you consume milk substitutes (soy milk, lactose free milk, almond milk, etc.)?

a. Never

b. Rarely (less than once/week

c. Occasionally (1-2 times/week)

d. Regularly (3-5 times/week)

e. Daily

73.) How often do you eat frozen desserts (ice cream/gelato/milkshakes, sherbet/sorbet, frozen yogurt, etc.)?

a. Never

b. Rarely (less than once/week

c. Occasionally (1-2 times/week)

d. Regularly (3-5 times/week)

e. Daily

***Meat/Seafood***

74.)In an average week, how often do you eat red meat?

a. Never

b. Rarely (less than once/week

c. Occasionally (1-2 times/week)

d. Regularly (3-5 times/week)

e. Daily

75.)In an average week, how often do you consume higher fat red meats like prime rib, T-bone steak, hamburger, ribs, bacon, etc.?

a. Never

b. Rarely (less than once/week

c. Occasionally (1-2 times/week)

d. Regularly (3-5 times/week)

e. Daily

76.)How many days in an average week do you consume chicken or turkey at least once a day?

a. Never

b. Rarely (less than once/week

c. Occasionally (1-2 times/week)

d. Regularly (3-5 times/week)

e. Daily

77.)How many days in an average week do you consume seafood (fish, shrimp, lobster, crab, etc.)?

a. Never

b. Rarely (less than once/week

c. Occasionally (1-2 times/week)

d. Regularly (3-5 times/week)

e. Daily

78.)How many days in an average week do you consume salted snacks (potato chips, nacho chips, corn chips, popcorn with butter, French fries etc.)?

a. Never

b. Rarely (less than once/week

c. Occasionally (1-2 times/week)

d. Regularly (3-5 times/week)

e. Daily

79.)How many days in an average week do you consume sugary sweets (cake, cookies, pastries, donuts, muffins, chocolate etc.) at least once a day?

a. Never

b. Rarely (less than once/week

c. Occasionally (1-2 times/week)

d. Regularly (3-5 times/week)

e. Daily

80.) Cook with olive oil?

a. Never

b. Rarely (less than once/week

c. Occasionally (1-2 times/week)

d. Regularly (3-5 times/week)

e. Daily

81.) Consume whole eggs (does not include egg beaters or just egg whites).

a. Never

b. Rarely (less than once/week

c. Occasionally (1-2 times/week)

d. Regularly (3-5 times/week)

e. Daily

82.) Drink 16 ounces or more of sugar sweetened beverages such as non-diet soda or fruit drink/punch (however, not including 100 % fruit juice) in a day? (1 can of soda = 12 ounces)

a. Never

b. Rarely (less than once/week

c. Occasionally (1-2 times/week)

d. Regularly (3-5 times/week)

e. Daily

82.) Consume diet beverages with artificial sweeteners?

a. Never

b. Rarely (less than once/week)

c. Occasionally (1-2 times/week

d. Regularly (3-5 times/week)

e. Daily

83.) Consume at least 1L (~32 ounces) of water in a day?

a. Never

b. Rarely (less than once/week

c. Occasionally (1-2 times/week)

d. Regularly (3-5 times/week)

e. Daily

The American Gut Project has made it possible for participants to make use of a Food Frequency Questionnaire (FFQ) to provide more valuable insights into what you are eating. Please go to **www.vioscreen.com** to complete the survey.

**American Gut Project**

SUPPLEMENTAL ANSWER SECTION

***You may decline to answer any question by leaving the space blank.***

1. Dietary Supplements/Dietary Restrictions.

2. Race /ethnicity:

3. Over the counter and prescription medication:

4. Travel:

5. What is your relationship to other people in this study who have voluntarily told you of their participation (e.g. partner, children, roommates)? For children, please specify whether or not you are genetically related. Note that we will only use information that both parties provide.

Participant This person is my…

6. Pets:

Indoor/outdoor or confined:

Contact extent:

7. Antibiotic/s:

Name:

Treatment for:

8. Pregnancy due date:

9. Other conditions you suffer from that are not listed

Please write anything else about yourself that you think could affect your personal microbiome

**Supplemental references**

1. [Jiang H, Ling Z, Zhang Y, Mao H, Ma Z, Yin Y, Wang W, Tang W, Tan Z, Shi J, Li L, Ruan B. 2015. Altered fecal microbiota composition in patients with major depressive disorder. Brain Behav Immun 48:186–194.](http://paperpile.com/b/zODTm0/LB7H)

2. [Zheng P, Zeng B, Zhou C, Liu M, Fang Z, Xu X, Zeng L, Chen J, Fan S, Du X, Zhang X, Yang D, Yang Y, Meng H, Li W, Melgiri ND, Licinio J, Wei H, Xie P. 2016. Gut microbiome remodeling induces depressive-like behaviors through a pathway mediated by the host’s metabolism. Mol Psychiatry 21:786–796.](http://paperpile.com/b/zODTm0/GrVC)

3. [Evans SJ, Bassis CM, Hein R, Assari S, Flowers SA, Kelly MB, Young VB, Ellingrod VE, McInnis MG. 2017. The gut microbiome composition associates with bipolar disorder and illness severity. J Psychiatr Res 87:23–29.](http://paperpile.com/b/zODTm0/ImlOr)

4. [Falony G, Joossens M, Vieira-Silva S, Wang J, Darzi Y, Faust K, Kurilshikov A, Bonder MJ, Valles-Colomer M, Vandeputte D, Tito RY, Chaffron S, Rymenans L, Verspecht C, De Sutter L, Lima-Mendez G, D’hoe K, Jonckheere K, Homola D, Garcia R, Tigchelaar EF, Eeckhaudt L, Fu J, Henckaerts L, Zhernakova A, Wijmenga C, Raes J. 2016. Population-level analysis of gut microbiome variation. Science 352:560–564.](http://paperpile.com/b/zODTm0/4WnYn)

5. [Zhernakova A, Kurilshikov A, Bonder MJ, Tigchelaar EF, Schirmer M, Vatanen T, Mujagic Z, Vila AV, Falony G, Vieira-Silva S, Wang J, Imhann F, Brandsma E, Jankipersadsing SA, Joossens M, Cenit MC, Deelen P, Swertz MA, LifeLines cohort study, Weersma RK, Feskens EJM, Netea MG, Gevers D, Jonkers D, Franke L, Aulchenko YS, Huttenhower C, Raes J, Hofker MH, Xavier RJ, Wijmenga C, Fu J. 2016. Population-based metagenomics analysis reveals markers for gut microbiome composition and diversity. Science 352:565–569.](http://paperpile.com/b/zODTm0/T2OAI)

6. [McDonald D, Ackermann G, Khailova L, Baird C, Heyland D, Kozar R, Lemieux M, Derenski K, King J, Vis-Kampen C, Knight R, Wischmeyer PE. 2016. Extreme Dysbiosis of the Microbiome in Critical Illness. mSphere 1.](http://paperpile.com/b/zODTm0/u0COM)

7. [Chase J, Fouquier J, Zare M, Sonderegger DL, Knight R, Kelley ST, Siegel J, Caporaso JG. 2016. Geography and Location Are the Primary Drivers of Office Microbiome Composition. mSystems 1.](http://paperpile.com/b/zODTm0/fu407)

8. [Mittal R, Coopersmith CM. 2014. Redefining the gut as the motor of critical illness. Trends Mol Med 20:214–223.](http://paperpile.com/b/zODTm0/AuPCA)

9. [Shimizu K, Ogura H, Hamasaki T, Goto M, Tasaki O, Asahara T, Nomoto K, Morotomi M, Matsushima A, Kuwagata Y, Sugimoto H. 2011. Altered gut flora are associated with septic complications and death in critically ill patients with systemic inflammatory response syndrome. Dig Dis Sci 56:1171–1177.](http://paperpile.com/b/zODTm0/HJMXq)

10. [Dethlefsen L, Huse S, Sogin ML, Relman DA. 2008. The pervasive effects of an antibiotic on the human gut microbiota, as revealed by deep 16S rRNA sequencing. PLoS Biol 6:e280.](http://paperpile.com/b/zODTm0/Amt3F)

11. [Hsu YJ, Chiu CC, Li YP, Huang WC, Huang YT, Huang CC, Chuang HL. 2015. Effect of intestinal microbiota on exercise performance in mice. J Strength Cond Res 29:552–558.](http://paperpile.com/b/zODTm0/9rQfy)

12. [Barton W, Penney NC, Cronin O, Garcia-Perez I, Molloy MG, Holmes E, Shanahan F, Cotter PD, O’Sullivan O. 2017. The microbiome of professional athletes differs from that of more sedentary subjects in composition and particularly at the functional metabolic level. Gut.](http://paperpile.com/b/zODTm0/JKreG)

13. [Karl JP, Margolis LM, Madslien EH, Murphy NE, Castellani JW, Gundersen Y, Hoke AV, Levangie MW, Kumar R, Chakraborty N, Gautam A, Hammamieh R, Martini S, Montain SJ, Pasiakos SM. 2017. Changes in intestinal microbiota composition and metabolism coincide with increased intestinal permeability in young adults under prolonged physiological stress. Am J Physiol Gastrointest Liver Physiol 312:G559–G571.](http://paperpile.com/b/zODTm0/gbo21)
