# Supplementary figures and images for: American Gut: an Open Platform for Citizen Science Microbiome Research
Source: mSystems. 2018 May 15;3(3):e00031-18. doi: 10.1128/mSystems.00031-18 (PMC5954204; doi:10.1128/mSystems.00031-18)

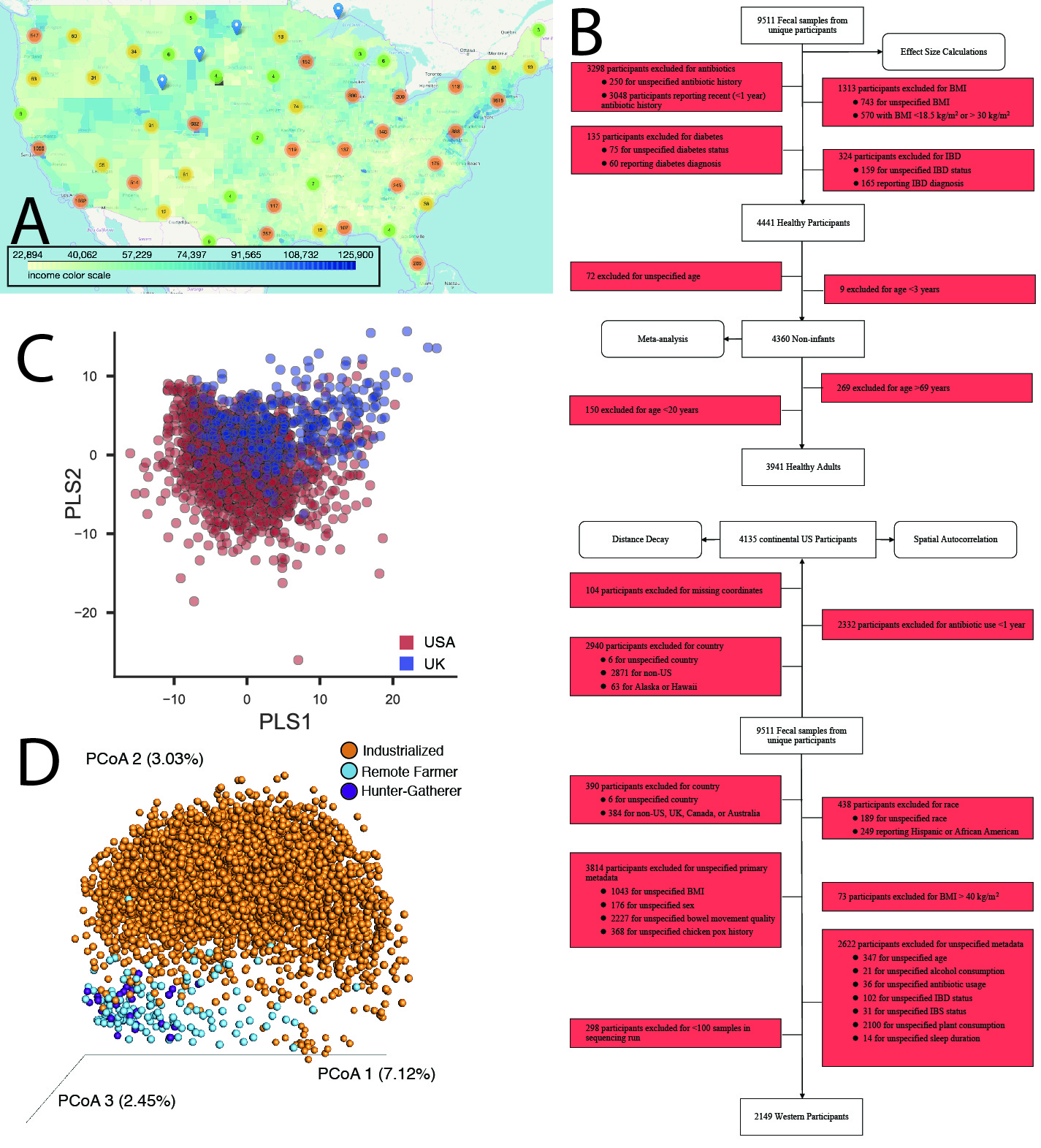

Supplement: FIG S1 [file sys003182229sf1.jpg]

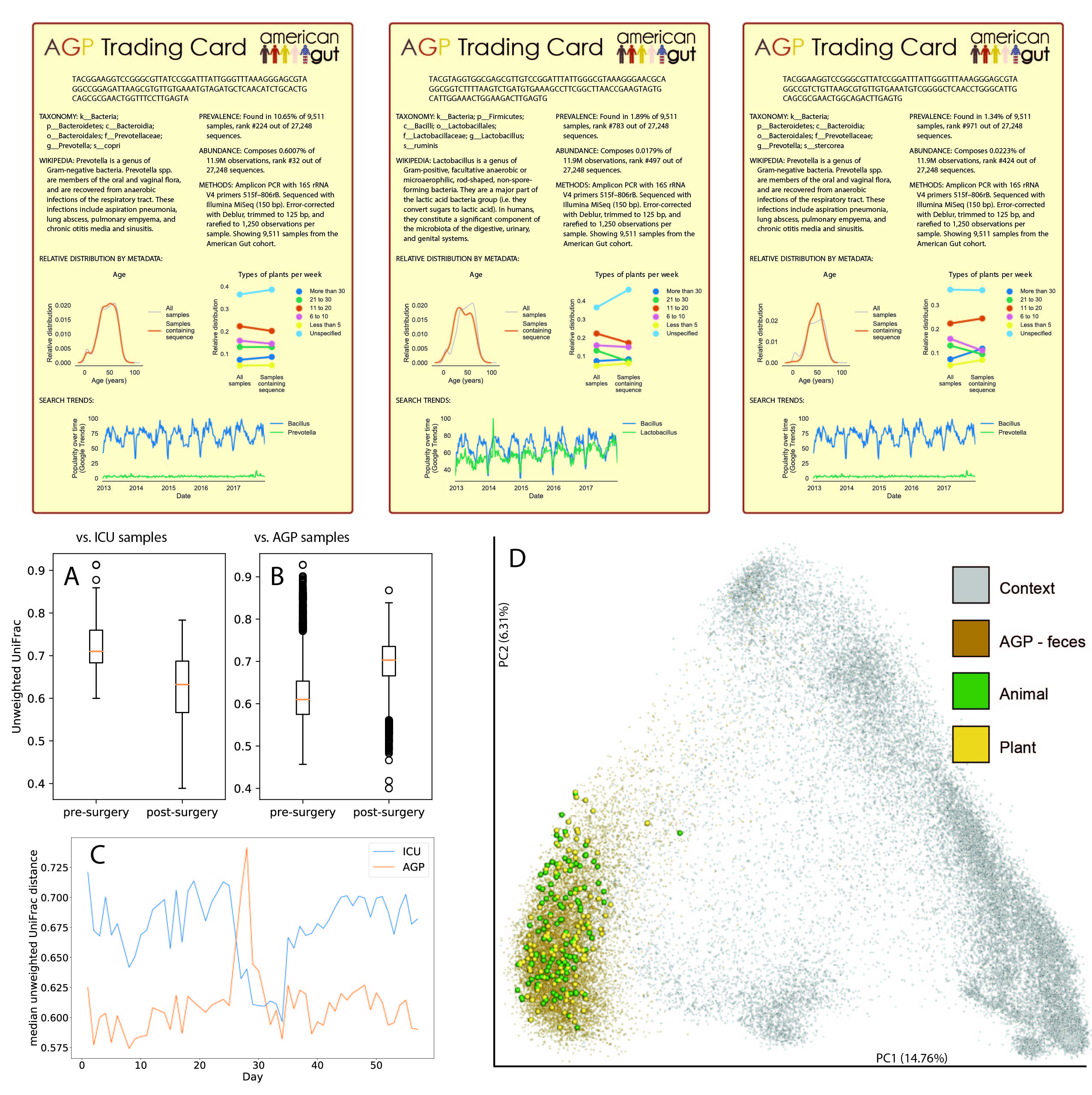

Supplement: FIG S2 [file sys003182229sf2.jpg]

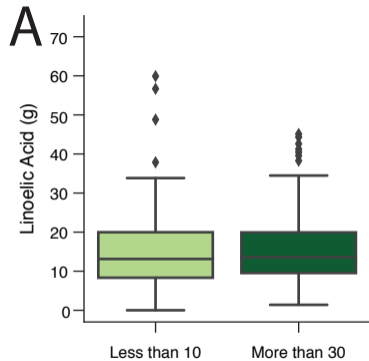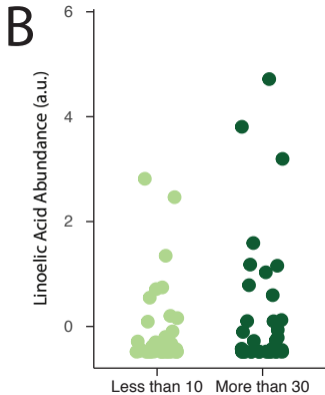

Supplement: FIG S3 [file sys003182229sf3.pdf]
