# Supplementary material for: American Gut: an Open Platform for Citizen Science Microbiome Research
Source: mSystems. 2018 May 15;3(3):e00031-18. doi: 10.1128/mSystems.00031-18 (PMC5954204; doi:10.1128/mSystems.00031-18)
Supplement: FIG S4 [file sys003182229sf4.pdf]

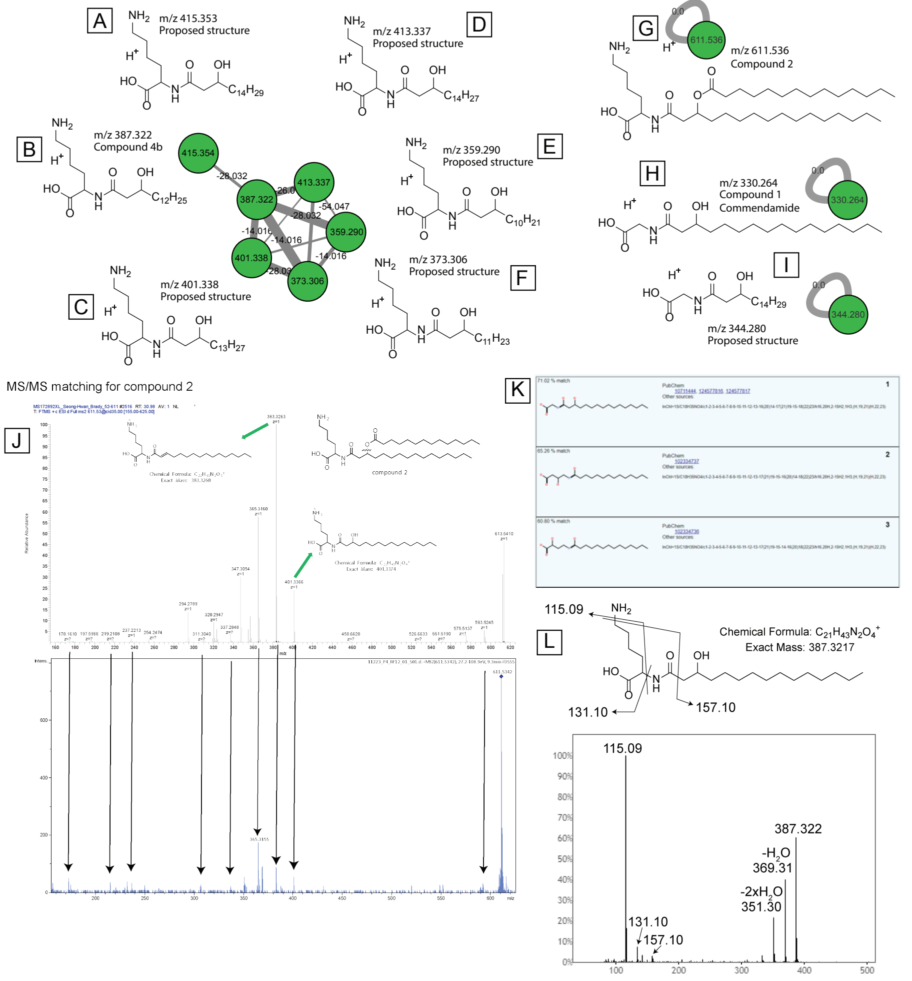

A
m/z 415.353
Proposed structure
B
m/z 387.322
Compound 4b
C
m/z 401.338
Proposed structure
D
m/z 413.337
Proposed structure
E
m/z 359.290
Proposed structure
F
m/z 373.306
Proposed structure
G
m/z 611.536
Compound 2
H
m/z 330.264
Compound 1
Commendamide
I
m/z 344.280
Proposed structure
415.354
413.337
387.322
359.290
401.338
373.306
611.536
330.264
344.280
-28.032
-54.047
-14.016
MS/MS matching for compound 2
J
compound 2
Chemical Formula: C22H43N2O3+
Exact Mass: 383.3268
Chemical Formula: C22H45N2O4+
Exact Mass: 401.3374
383.3263
365.3160
347.3054
401.3366
613.5410
611.5342
365.3155
Relative Abundance
m/z
K
71.02 % match
65.26 % match
60.80 % match
PubChem
Other sources
L
115.09
131.10
157.10
Chemical Formula: C21H43N2O4+
Exact Mass: 387.3217
387.322
-H2O
369.31
-2xH2O
351.30
